# Computational Exploration of Dirhodium Complex-Catalyzed Selective Intermolecular Amination of Tertiary vs. Benzylic C−H Bonds

**DOI:** 10.3390/molecules28041928

**Published:** 2023-02-17

**Authors:** Xing-Xing Su, Xia-He Chen, De-Bo Ding, Yuan-Bin She, Yun-Fang Yang

**Affiliations:** College of Chemical Engineering, Zhejiang University of Technology, Hangzhou 310014, China

**Keywords:** mechanism, selectivity, amination, benzylic C–H, tertiary C–H

## Abstract

The mechanism and origins of site-selectivity of Rh_2_(*S*-tfpttl)_4_-catalyzed C(*sp*^3^)–H bond aminations were studied using density functional theory (DFT) calculations. The synergistic combination of the dirhodium complex Rh_2_(*S*-tfpttl)_4_ with *tert*-butylphenol sulfamate TBPhsNH_2_ composes a pocket that can access both tertiary and benzylic C–H bonds. The nonactivated tertiary C–H bond was selectively aminated in the presence of an electronically activated benzylic C–H bond. Both singlet and triplet energy surfaces were investigated in this study. The computational results suggest that the triplet stepwise pathway is more favorable than the singlet concerted pathway. In the hydrogen atom abstraction by Rh–nitrene species, which is the rate- and site-selectivity-determining step, there is an attractive π–π stacking interaction between the phenyl group of the substrate and the phthalimido group of the ligand in the tertiary C–H activation transition structure. By contrast, such attractive interaction is absent in the benzylic C–H amination transition structure. Therefore, the DFT computational results clearly demonstrate how the synergistic combination of the dirhodium complex with sulfamate overrides the intrinsic preference for benzylic C–H amination to achieve the amination of the nonactivated tertiary C–H bond.

## 1. Introduction

In the past several decades, the transition metal rhodium has gradually gained widespread interest as an efficient catalyst due to both its versatility and wide application in C–H bond functionalization reactions [1,2]. Since the 1920s, catalytic C–H amination reactions have been a valuable synthetic strategy to functionalize C–H bonds [3,4,5,6]. Dirhodium complex-catalyzed C–H aminations with high chemoselectivity and regioselectivity have been developed [7,8,9,10,11,12,13]. A number of excellent works by Dauban and colleagues describe the intermolecular C–H amination by a chiral rhodium(II) catalyst [14,15]. The reaction results in high yields and excellent chemo- and diastereoselectivities in various benzylic and allylic substrates [14]. A similar intermolecular amination of benzylic C–H bonds was achieved using the same chiral rhodium(II) catalyst, in which the reaction of various benzylic and allylic substrates with enantiomerically pure sulfonimidamide resulted in good to excellent yields and excellent diastereoselectivities [15]. In 2016, Singh and colleagues achieved an intramolecular C–H amination reaction by using a Rh_2_(OAc)_4_ catalyst via an electrophilic substitution pathway, in which the reaction underwent selective aromatic C(*sp*^2^)–H amination over more labile *o*-C(*sp*^3^)–H bonds [16]. In 2017, Falck and colleagues introduced *N*-*^t^*Boc-protected OTs hydroxylamines as precursors to alkyl Rh-nitrenes, and reported the catalyst-controlled regio- and diastereoselectivity of aliphatic C(*sp*^3^)–H aminations [17]. Dang and colleagues investigated the reaction mechanism and origins of diastereoselectivity of dirhodium-catalyzed C(*sp*^3^)–H aminations, and their density functional theory (DFT) studies suggested that the substrate–ligand steric repulsions impact the diastereoselectivity in this reaction [18]. In 2021, Phipps and colleagues exploited a series of ion-paired chiral catalysts for dirhodium-catalyzed enantioselective intermolecular C–H amination based on the esp ligand scaffold, which improved yields compared with Rh_2_(esp)_2_ [19].

Generally, the site-selectivity of dirhodium-catalyzed undirected C–H amination reactions is dominated by the intrinsic bond dissociation energy (BDE) of C–H bonds [20,21,22,23,24,25,26]. Thus, aminated tertiary or benzylic C–H bonds are preferred compared to primary and other secondary C–H bonds [27,28,29]. In 2007, J. Du Bois and colleagues reported that the Rh_2_(esp)_2_-catalyzed intermolecular C–H amination of alkanes undergoes via a concerted asynchronous two-electron oxidation pathway [30]. The reaction is remarkably selective for benzylic C–H bonds; however, the tertiary C–H bonds afforded a very low product yield [30]. Subsequently, J. Du Bois and colleagues found that the Rh-catalyzed amination reaction (with electron-poor DfsNH_2_ as the nitrogen source) could achieve the tertiary C–H bond amination with high efficiency. The experimental results also indicated that the nitrogen source determines the site-selectivity of the product in this Rh-catalyzed intramolecular C–H amination [31]. In substrates displaying both tertiary and benzylic C–H bonds, secondary benzylic C–H bonds are normally more activated and preferred to be functionalized with high benzylic-to-tertiary site-selectivity (B:T ratio) [32]. J. Du Bois’s group reported that the combination of Rh_2_(esp)_2_ (esp = *α*,*α*,*α*′,*α*′-tetramethyl-1,3-benzenedipropanoate) with CF_3_CH_2_SO_3_NH_2_ accomplished the selective intermolecular amination of isoamylbenzene substrate with a B:T ratio of 85:1 [33]. The mechanism and site-selectivity of such intermolecular amination reactions were studied computationally by Wang and colleagues [34]. They found that intermolecular benzylic and tertiary C–H aminations proceed predominantly via a stepwise triplet pathway. The strong electron-donating substituent of the substrate can reduce the barrier of benzylic C–H amination via a *p*–π conjugation, which suggests that the different electronic properties of substituents have a remarkable influence on B/T ratios.

**Figure 1:**
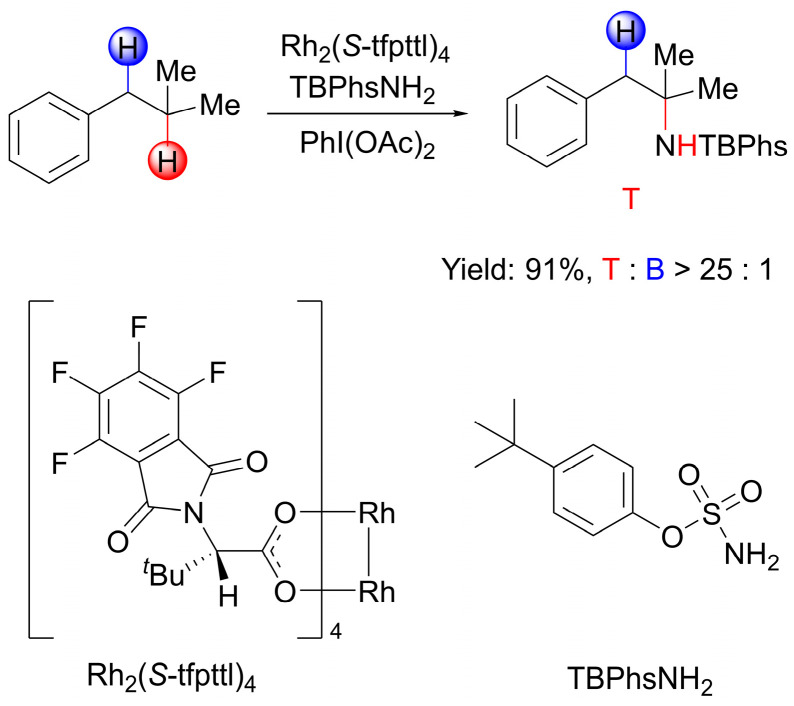
Rh_2_(*S*-tfpttl)_4_-catalyzed intermolecular C(*sp*^3^)−H amination of isobutylbenzene.

Philippe Dauban and colleagues developed a chiral rhodium(II) complex catalyzed C(*sp*^3^)−H amination reaction for the asymmetric synthesis of benzylic amines [35]. Recently, their group reported the catalytic intermolecular amination of the nonactivated tertiary C(*sp*^3^)−H bond in substrates displaying an activated benzylic C(*sp*^3^)−H bond with a T/B ratio of >25:1 (Figure 1) [36]. In this transformation, only 0.01 mol % of the dirhodium(II) tetrakis [*N*-tetrafluorophthaloyl-(*S*)-*tert*-leucinate] complex Rh_2_(*S*-tfpttl)_4_ was loaded as catalyst, and *tert*-butylphenol sulfamate TBPhsNH_2_ was selected as the aromatic sulfamate. Aliphatic sulfamates, such as trichloroethyl sulfamate (TcesNH_2_) [30] or pentafluorobenzyl sulfamate (PfbsNH_2_) [37], were not satisfactory, as the T/B ratio did not exceed 3:1. In addition, the reaction performed using the combination of TBPhsNH_2_ with the Rh_2_(esp)_2_ complex also led to a poor T/B ratio of 1:1. It is interesting that only the combination of complex Rh_2_(*S*-tfpttl)_4_ with TBPhsNH_2_ achieved a high T/B ratio [36], which motivated us to investigate the origin of this unusual site-selectivity computationally. Herein, we report a detailed computational study of the mechanism of Rh_2_(*S*-tfpttl)_4_-catalyzed intermolecular amination of tertiary vs. benzylic C–H bonds and the factors controlling site-selectivity.

**Figure 2:**
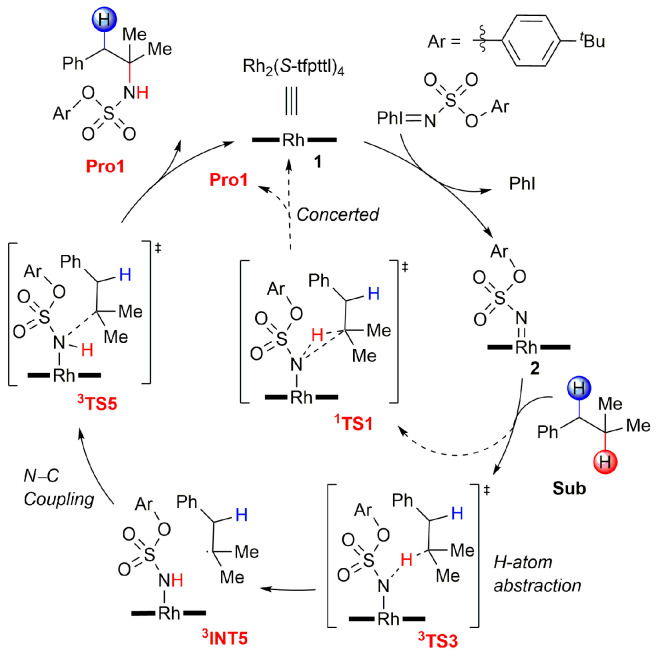
Proposed reaction mechanisms for Rh_2_(*S*-tfpttl)_4_-catalyzed intermolecular amination of tertiary C–H bonds. Ar = *p*-*tert*-butylphenyl.

Both the singlet concerted and triplet stepwise mechanisms for Rh_2_(*S*-tfpttl)_4_-catalyzed intermolecular amination of tertiary C–H bonds were proposed (Figure 2) [34,38,39,40,41,42,43]. Starting from the catalyst Rh_2_(*S*-tfpttl)_4_ **1**, TBPhsNH_2_, as nitrogen source, combines with the catalyst to generate the active Rh–nitrene intermediate **2**. Both the singlet and triplet states of this Rh–nitrene intermediate **2** may be involved in subsequent transformations. The singlet Rh–nitrene **^1^2** undergoes a concerted H-abstraction/C–N bond formation via transition state **^1^TS1** to generate aminated products. The triplet Rh–nitrene **^3^2** reacts with isobutylbenzene in a stepwise pathway, in which hydrogen atom abstraction occurs first to generate a triplet diradical intermediate **^3^INT5** via transition state **^3^TS3**, and then a C–N bond forms to generate the final amination product **Pro1** via transition state **^3^TS5**. The intermolecular amination at the benzylic site is postulated to follow the exact same mechanisms as the tertiary site.

## 2. Results

### 2.1. The Dirhodium Complex Rh_2_(S-tfpttl)_4_

**Figure 3:**
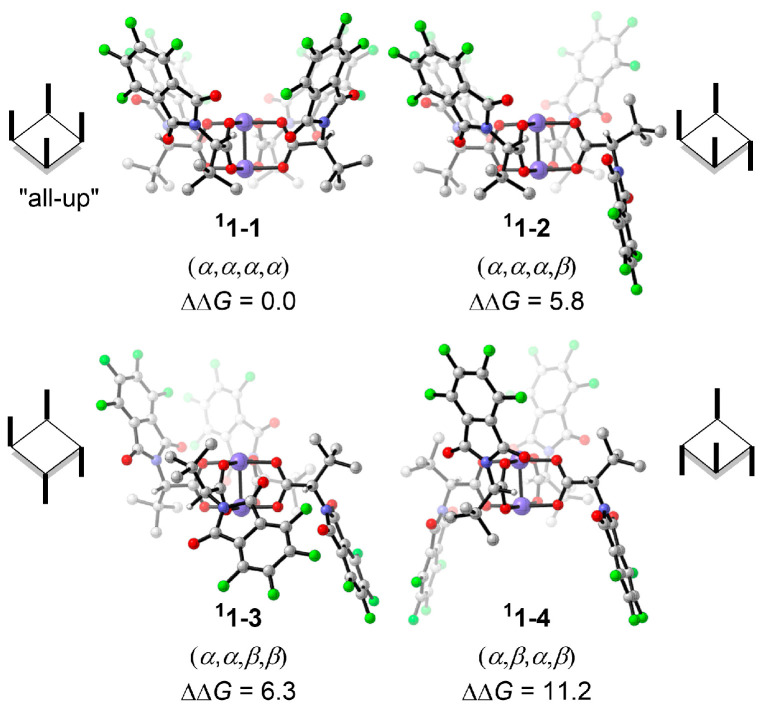
Optimized geometries and energies of dirhodium catalyst conformers. Free energy obtained at the M06/def2-TZVP//BPW91/6-31G*-LANL2DZ level. Energies are shown in kcal mol^−1^. The H atoms of *^t^*Bu groups are omitted for clarity.

The dirhodium tetracarboxylates complex Rh_2_(*S*-tfpttl)_4_, which is derived from *α*-*N*-(phthaloyl) amino acids, is known to adopt various conformers, including *α*,*α*,*α*,*α* (all-up), *α*,*α*,*α*,*β*, *α*,*α*,*β*,*β*, and *α*,*β*,*α*,*β*, depending on the orientation of substituents (Figure 3) [42,44,45,46,47]. The so-called “all-up” conformer of Rh_2_(*S*-tfpttl)_4_ (**^1^1-1**) is the most stable, which is consistent with its X-ray crystal structure [36]. Therefore, the calculations of this catalytic system are based on the “all-up” conformer. In the “all-up” conformer, the four phthalimido groups shape a wider pocket than the one formed by the *^t^*Bu groups (Figure S1); therefore, nitrene binds to the broader face of the catalyst. The triplet state **^3^1-1** is 10.1 kcal mol^−1^ higher in energy than the singlet state **^1^1-1** (Figure S2).

### 2.2. The Dirhodium–Nitrene Complex

**Figure 4:**
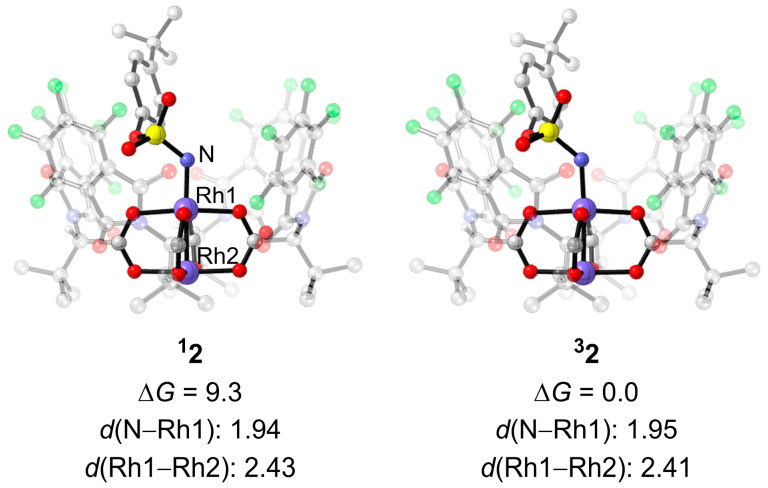
Optimized geometries and energies of the singlet and triplet states of dirhodium–nitrene. Energies are in kcal mol^−1^. Free energy obtained at the ONIOM(M06/def2-TZVP:UFF//BPW91/ 6-31G*-LANL2DZ:UFF) level. The H atoms of *^t^*Bu groups and aryl groups are omitted for clarity.

A previous study suggested that dirhodium–nitrene is the active species responsible for nitrene insertion into the C–H bond [38]. As shown in Figure S3, the dirhodium–nitrene complex is formed from dirhodium catalyst **1** and TBPhsN=IPh. The formation of the singlet dirhodium–nitrene is endothermic by 6.2 kcal mol^−1^ thermodynamically, and the formation of the triplet dirhodium–nitrene is exergonic by 13.2 kcal mol^−1^ thermodynamically. The singlet and triplet dirhodium–nitrene complexes are denoted as **^1^2** and **^3^2,** respectively. The closed-shell and the open-shell singlet dirhodium–nitrene were both calculated, and their energies are very close (Table S1). In the geometry of the singlet dirhodium–nitrene complex **^1^2**, the bond lengths of N–Rh1 and Rh1–Rh2 are 1.94 Å and 2.43 Å, respectively. In the triplet dirhodium–nitrene complex **^3^2**, the distance of N–Rh1 (1.95 Å) and Rh1–Rh2 (2.41 Å) are close to the corresponding distances in the singlet dirhodium–nitrene complex (Figure 4). It should be noted that the triplet state intermediate **^3^2** is more stable than the singlet state **^1^2**. The energy difference is calculated to be 9.3 kcal mol^−1^ (Figure 4). The spin densities on dirhodium (0.718) and the N atom (0.960) show that the two unpaired electrons are delocalized on the Rh2–Rh1–N moiety in the **^3^2** (Table S2). The one unpaired electron on the nitrene N atom suggests that the N atom has radical-type reactivity. This facilitates the hydrogen atom abstraction step in the triplet pathway, vide infra. In addition, two different combination modes of dirhodium–nitrene were investigated. The nitrene binding to the phthaloyl face is more stable than that binding to the *tert*-butyl face of the dirhodium catalyst (Figure S4) [36,48].

### 2.3. Singlet Pathway

**Figure 5:**
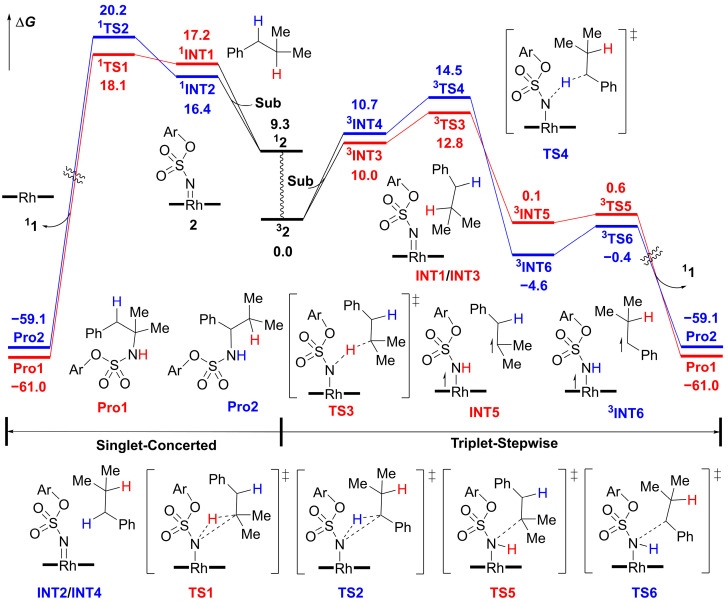
Free energy profiles of Rh_2_(*S−*tfpttl)_4_−catalyzed intermolecular C–H aminations. Free energy obtained at the ONIOM(M06/def2−TZVP:UFF//BPW91/6−31G*−LANL2DZ:UFF) level. Energies are shown in kcal mol^−1^. Ar = *p−tert−*butylphenyl.

**Figure 6:**
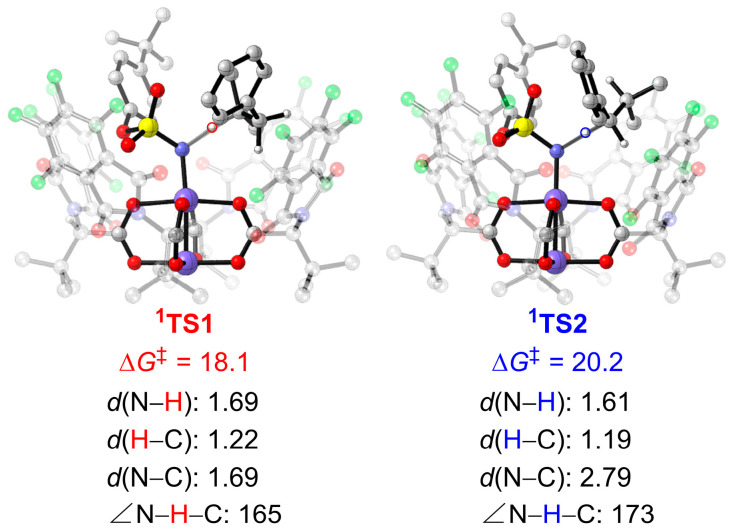
Optimized geometries and energies of **^1^TS1** and **^1^TS2**. The distances are in Å, the angles are in degrees, and energies are shown in kcal mol^−1^. Free energy obtained at the ONIOM (M06/def2−TZVP:UFF//BPW91/6−31G*−LANL2DZ:UFF) level. The H atoms of *^t^*Bu groups and aryl groups are omitted for clarity.

Figure 5 shows both the singlet and triplet free energy profiles for Rh_2_(*S*-tfpttl)_4_-catalyzed intermolecular amination of tertiary and benzylic C–H bonds. The singlet and triplet dirhodium–nitrene complexes, **^1^2** and **^3^2**, are structurally similar but undergo different C–H amination mechanisms. The singlet state **^1^2** undergoes the concerted pathway, and the triplet state **^3^2** goes through the stepwise pathway. Starting from the singlet state **^1^2**, the tertiary C–H bond amination occurs through **^1^TS1** (18.1 kcal mol^−1^) in a concerted mechanism that leads directly to the aminated product **Pro1**. In transition structure **^1^TS1**, the distance of the activated C–H bond is 1.22 Å, the distance of the forming N–H bond is 1.69 Å, and the N–H–C angle is found to be 165° (Figure 6). Similarly, the benzylic C–H bond amination proceeds via a concerted transition structure **^1^TS2**, which requires a barrier of 20.2 kcal mol^−1^, to generate the aminated product **Pro2**. However, the tertiary C–H bond amination is slightly favored over the benzylic C–H bond amination in the singlet pathway.

**Figure 7:**
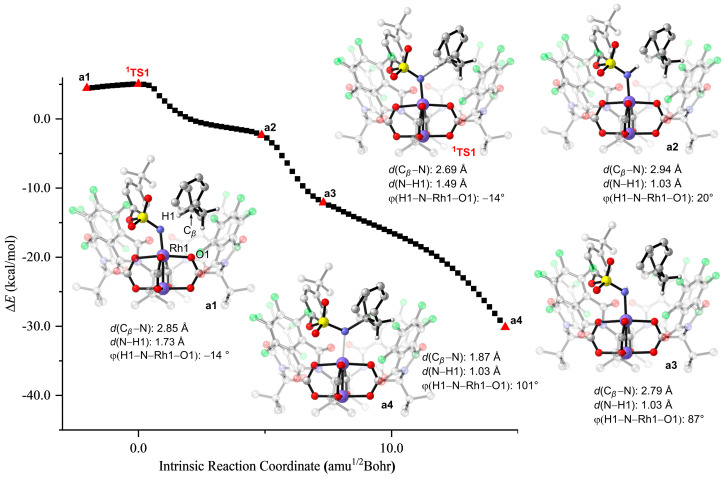
The IRC path for **^1^TS1**. The H atoms of *^t^*Bu groups and aryl groups are omitted for clarity.

Several attempts to optimize the open-shell singlet radical intermediate failed, but it eventually converged to the closed-shell singlet amination product. Similar to the rhodium(II)-catalyzed C–H aminations using *N*-mesyloxycarbamates reported by Hélène Lebel and colleagues [46], the singlet dirhodium-nitrene species undergoes concerted C–H amination and the triplet dirhodium–nitrene species goes through a stepwise radical pathway. To further verify that the transition state **^1^TS1** connects the intermediate **^1^INT1** and the amination product, the intrinsic reaction coordinate (IRC) was calculated (Figure 7). Corresponding geometric information of selected points on the IRC pathway is also given in Figure 7. The IRC connects the intermediate **^1^INT1** at **a1**. In the geometry of **a2**, which is on the shoulder of the energy surface, the N–H bond is forming (*d*(N–H) = 1.03 Å) but the N–C bond is still unformed (*d*(C*_β_*–N) = 2.94 Å). From the geometry of **a2** to **a3**, there are only subtle changes to bond lengths and angles. Interestingly, the dihedral angle of H1–N–Rh1–O1 is 20° in **a2** but changes to 87° in **a3**. This dramatic change clearly demonstrates that the H1 atom rotates around the Rh1−N axis to provide space for the subsequent N–C*_β_* formation. In the geometry of **a4**, the N–C*_β_* bond distance is shortened to 1.87 Å. The IRC calculation results clearly illustrate that **^1^TS1** is a concerted and highly asynchronous transition structure [34,40,41,49]. The potential energy surface along the IRC calculation for the benzylic C–H amination transition structure **^1^TS2**, which is shown in Figure S5, is similar to that of **^1^TS1**.

### 2.4. Triplet Pathway

**Figure 8:**
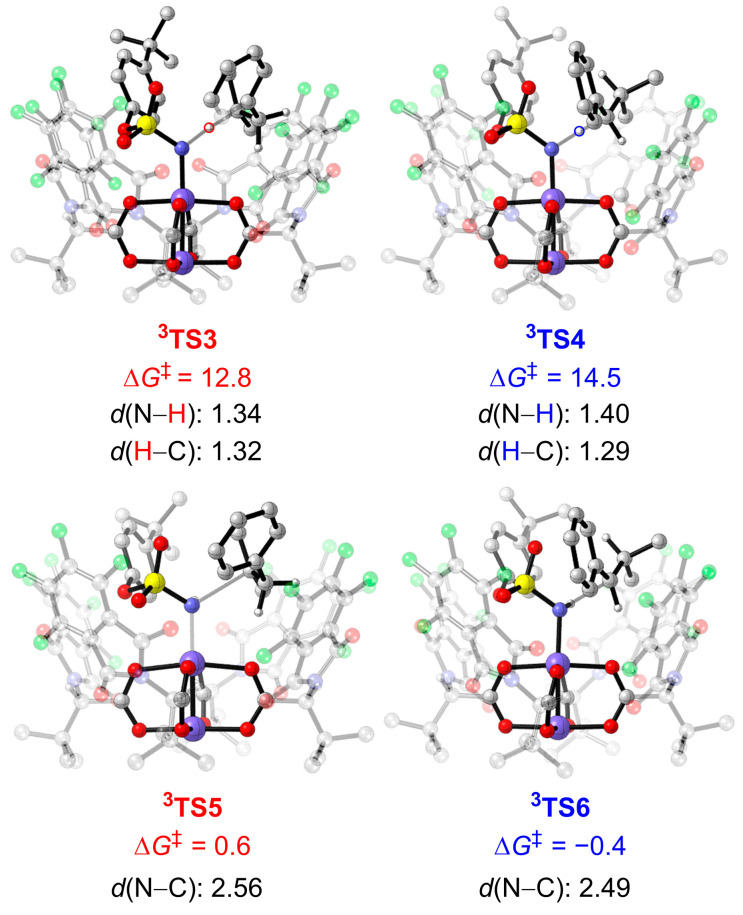
Optimized geometries and energies of **^3^TS3**, **^3^TS4**, **^3^TS5** and **^3^TS6**. The distances are in Å, and energies are shown in kcal mol^−1^. Free energy obtained at the ONIOM(M06/def2−TZVP:UFF//BPW91/6−31G*−LANL2DZ:UFF) level. The H atoms of *^t^*Bu groups and aryl groups are omitted for clarity.

The free energy profiles for the triplet pathway are shown in Figure 5 and Figure S6. From the dirhodium–nitrene complex **^3^2**, the H-atom abstraction from the tertiary C–H bond of the substrate occurs via **^3^TS3** (Figure 8), which requires a barrier of 12.8 kcal mol^−1^, leading to intermediate **^3^INT5**. The bond lengths of the forming N−H bond and cleaving C−H bond in the transition state **^3^TS3** are 1.34 Å and 1.32 Å, respectively. The total spin density on the Rh2–Rh1–N moiety of the **^3^INT5** is 0.946, and the spin density on the C*_β_* atom of the **^3^INT5** is 0.827, which indicates that **^3^INT5** is a triplet diradical intermediate (Table S3). Subsequently, the substrate radical rebounds to the nitrogen atom of the nitrene moiety, forming the C–N bond. This radical rebound step requires a barrier of 0.5 kcal mol^−1^ via **^3^TS5** with respect to the preceding intermediate **^3^INT5**. The total spin density on the dirhodium moiety varies significantly from 0.596 in **^3^INT5** to 1.412 in **^3^TS5**, which shows that the unpaired electrons transfer partly to the Rh1–Rh2 moiety (Table S3). Finally, the tertiary C−H bond amination product **Pro1** dissociates from the dirhodium complex to regenerate the catalyst **1**.

For cleavage of the benzylic C−H bond of the substrate, the hydrogen atom abstraction via **^3^TS4** is the rate-determining step, which requires a barrier of 14.5 kcal mol^−1^. The bond lengths of the forming N−H and cleaving C−H bonds in the transition state **^3^TS4** are 1.40 Å and 1.29 Å, respectively. The hydrogen atom transfer from the substrate to the dirhodium–nitrene complex **^3^2** produces the diradical intermediate **^3^INT6**. The total spin density on the Rh2–Rh1–N moiety of the **^3^INT6** is 0.925, and the spin density on the C*_β_* atom of the intermediate **^3^INT6** is 0.678 (Table S3). The diradical intermediate **^3^INT6** is 4.7 kcal mol^−1^ lower in energy than the corresponding intermediate **^3^INT5** in the tertiary C−H bond amination process. This is mainly caused by a favorable *p*–π conjugation interaction between the unpaired electrons of the C*_α_* atom and the adjacent phenyl group in **^3^INT6** (Table S3). The rebound step of forming the C*_α_*−N bond via **^3^TS6** requires a barrier of 4.2 kcal mol^−1^ with respect to the preceding intermediate **^3^INT6**.

The hydrogen atom abstraction step is the rate-limiting step in this reaction. DFT computational results suggest that the triplet stepwise mechanism is more favorable than the singlet concerted mechanism for either tertiary or benzylic C–H bond amination. In the triplet stepwise mechanism, when compared with the benzylic C–H bond amination via **^3^TS4** the tertiary C–H bond amination via **^3^TS3** is favored by 1.7 kcal mol^−1^, corresponding to a computational T:B ratio of 18:1, which is slightly lower than the experimental T:B ratio of 25:1. In addition, we optimized these two key transition-state structures using the full quantum mechanics method (Figure S7). The energy difference between **^3^TS3′** and **^3^TS4′** is 2.1 kcal mol^−1^, which is expected to lead to a T:B ratio of 35:1. Therefore, applying different computational methods results in only subtle energy changes, and they are all consistent with experimental site-selectivity. The computational results show that the intrinsic preference for benzylic C−H bond amination is overridden by the nonactivated tertiary C–H bond amination in this catalytic system.

### 2.5. Origins of Site-Selectivity

**Figure 9:**
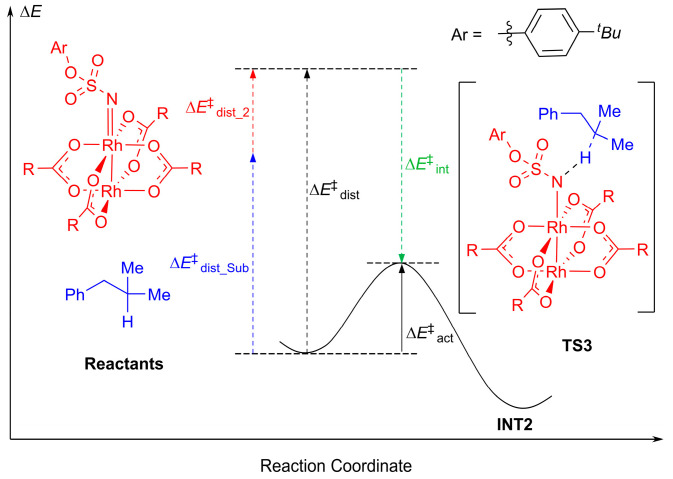
Distortion/interaction model.

**Figure 10:**
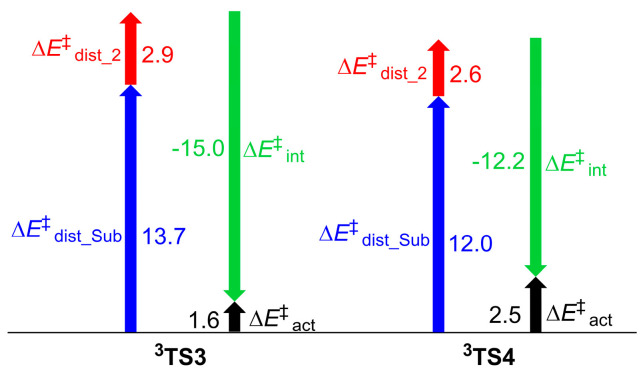
Distortion/interaction analysis of the two transition states in the hydrogen atom abstraction step. Energies are in kcal mol^−1^, and were obtained at the ONIOM(M06/def2−TZVP:UFF //BPW91/6−31G*−LANL2DZ:UFF) level.

To gain insight into the origins of site-selectivity of Rh_2_(*S*-tfpttl)_4_-catalyzed intermolecular C–H aminations, we performed a distortion/interaction analysis of the molecular fragments participating in the two transition states, **^3^TS3** and **^3^TS4**. The distortion/interaction model has been widely used to understand the origins of reactivities and selectivities [50,51,52,53,54,55]. This model links activation energy with the distortion energy required for the geometrical deformation of reactants achieving their transition-state geometry, as well as with the interaction energy generated by the interactions between the two distorted reactants in the transition state structure [56,57]. Figure 9 shows the distortion/interaction model of the tertiary C–H bond amination. The distortion energy ∆*E*^‡^_dist_ is composed of the distortion energies of dirhodium–nitrene (∆*E*^‡^_dist_2_) and the substrate (∆*E*^‡^_dist_Sub_). The activation energy ∆*E*^‡^_act_ of the reaction is the sum of the distortion energy ∆*E*^‡^_dist_ and the interaction energy ∆*E*^‡^_int_ between the two distorted species. Figure 10 shows that the activation energy ∆*E*^‡^_act_ of **^3^TS3** is lower than that of **^3^TS4** by 0.9 kcal mol^−1^. The interaction energy ∆*E*^‡^_int_ of **^3^TS3** is more favorable than that of **^3^TS4** by 2.8 kcal mol^−1^. The distortion energies of the dirhodium–nitrene part are similar in the two transition states, **^3^TS3** and **^3^TS4**. The benzylic C–H bond is more sterically accessible compared to the tertiary C–H bond. The distortion energy of the substrate in **^3^TS3** is higher than that in **^3^TS4** by 1.7 kcal mol^−1^, which is compensated for by stronger interactions between dirhodium–nitrene and substrate fragments in **^3^TS3**. This suggests that a favorable interaction between dirhodium–nitrene and the substrate in the transition state structure **^3^TS3** is the primary factor that controls site-selectivity.

**Figure 11:**
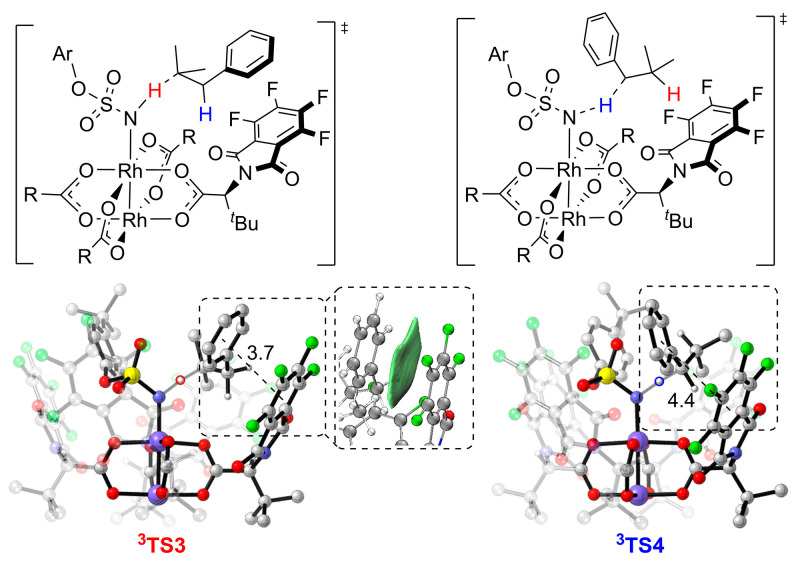
IGM analysis of **^3^TS3** and **^3^TS4**. Ar = *p*−*tert*−butylphenyl. The distances are in Å. The H atoms of *^t^*Bu groups and aryl groups are omitted for clarity.

Next, independent gradient model (IGM) analysis was performed to clearly show the favorable π−π stacking interaction between the two aromatic groups of the substrate and the ligand in **^3^TS3**. As shown in Figure 11, the phenyl group on the substrate is approximately parallel to the phthalimido group on the ligand of **^3^TS3**, and the distance between the two aromatic rings is about 3.7 Å, leading to a favorable attractive π–π stacking interaction (Figure S8) [58]. In **^3^TS4**, the corresponding distance between the two aromatic rings is 4.4 Å, and the phenyl group on the substrate is tilted away from the phthalimido group on the ligand. Thus, the abovementioned π–π stacking interaction is absent in **^3^TS4**.

Therefore, the favorable interaction energy in **^3^TS3** is mainly due to an attractive π–π stacking interaction, which offsets its unfavorable steric effect. In other words, such an attractive π–π stacking interaction between substrate and catalyst overrides the intrinsic electronic effects to render the nonactivated tertiary C–H bond of the substrate selectively aminated. In a truncated model study, the tertiary and benzylic C–H bond aminations by the dirhodium catalyst Rh_2_(O_2_CH)_4_ without the phthalimido group were also computed. The computational results show that the benzylic C–H bond amination is slightly favored by 1.0 kcal mol^−1^ compared with the tertiary C–H bond amination (Figure S9a). In addition, the “*α*,*α*,*α*,*β*” catalyst conformer without the above-mentioned π−π stacking interaction was also studied, and the benzylic C–H bond amination is more favorable than the tertiary C–H bond amination by 0.7 kcal mol^−1^ (Figure S9b). These results further support the idea that site-selectivity arises from the stabilizing π−π stacking interaction between the phenyl group on the substrate and the phthalimido group on the ligand in tertiary C–H bond amination transition structures.

## 3. Computational Method

All calculations were carried out with the Gaussian 16 package [59]. For computational efficiency, the whole system was divided into two layers by employing the ONIOM [60,61,62,63,64] approach: a “high-level (HL) layer”, treated at the DFT level, and a “low-level (LL) layer”, treated at the classical MM level (see Figure S10 in Supporting Information for the detailed ONIOM layers). Geometry optimization and energy calculations were performed with BPW91 functional in the high layer [34,65,66]. The LANL2DZ basis set [67,68] with ECP was used for Rh and I atoms, and the 6-31G* basis set [69,70,71] was used for other atoms. The low layer was treated with the universal force field (UFF) method, which is less computationally expensive [72,73]. Frequency analysis was conducted at the same level of theory, both to verify the stationary points as real minima or saddle points and to obtain thermodynamic energy corrections. A stability test was carried out with the Gaussian keyword “*stable* = *opt*” to ensure that the correct unrestricted wave functions were obtained. For the open-shell singlet state, we used keywords “*guess* = *mix*” and “*stable* = *opt*” to obtain the correct wave function at the initial geometry, and then performed geometry optimization for the structures using the optimized wave function as an initial guess with the keyword “*guess* = *read*”. In order to ensure that the optimized geometries had the correct wave-function, the same procedure was repeated on the optimized geometries. The single-point energies were calculated at the ONIOM (M06 [74]/def2-TZVP [75,76]:UFF) level. Computed structures were illustrated using CYLview [77]. Independent gradient model (IGM) [78,79] analysis was performed on the Multiwfn [80] software package, and the visualization of IGM analysis results was performed with VMD [81] visualization software. The conformers for the complex structures involved in this study are shown in Supplementary Materials (Figures S11–S18). To further justify the reliability of the BPW91 functional to describe this reaction, we performed calculations on the key transition states using M06, M06L-D3 [82], MN15 [83] and *w*B97xD [84] functionals as shown in the Supplementary Materials (Table S4 and Figure S19), which displayed the same trend as those found in the BPW91 functional. The energies and free energies of the calculated structures are shown in Table S5, and the cartesian coordinates of the structures are shown in the Supporting Materials.

## 4. Conclusions

In summary, we have clarified the mechanism and the origins of site-selectivity of Rh_2_(*S*-tfpttl)_4_-catalyzed C(*sp*^3^)–H bond amination reactions. According to computational results, the “all-up” conformer of Rh_2_(*S*-tfpttl)_4_ is the most stable conformer of the four possible conformers. In this conformer, the binding pocket shaped by four phthalimido groups is wider than the one formed by the *^t^*Bu groups. The nitrene binding to the phthaloyl face is more stable than that binding to the *tert*-butyl face of the dirhodium catalyst.

Both singlet and triplet energy surfaces were investigated in this study. DFT results showed that the singlet dirhodium-nitrene complex undergoes concerted and highly asynchronous C–H amination, while the triplet dirhodium–nitrene complex goes through a stepwise pathway. In the concerted pathway, the dirhodium–nitrene complex **^1^2** abstracts a hydrogen atom from the substrate to form the final aminated product. In the stepwise pathway, the dirhodium–nitrene complex **^3^2** abstracts a hydrogen atom from the substrate to generate a diradical intermediate, followed by radical rebound to generate the final aminated product. The dirhodium-nitrene complex abstracts a hydrogen atom from the tertiary C−H bond via singlet **^1^TS1**, but is disfavored by 5.3 kcal mol^−1^ compared to the corresponding triplet **^3^TS3**. Similarly, the dirhodium-nitrene complex abstracts a hydrogen atom from benzylic C−H bond via **^1^TS2** that is disfavored by 5.7 mol^−1^ compared to the corresponding triplet **^3^TS4**. Thus, the reaction proceeds via a stepwise hydrogen atom abstraction/radical rebound pathway in either the tertiary or the benzylic C–H bond amination, and the hydrogen atom abstraction step is the rate- and site-selectivity-determining step. The hydrogen atom abstraction transition state **^3^TS3** for the tertiary C–H amination is more favorable than **^3^TS4** for the benzylic C–H amination by 1.7 kcal mol^−1^, which is consistent with experimental results demonstrating that the tertiary C–H amination product **Pro1** is the major product.

To understand the origins of reactivities and selectivities, distortion/interaction analysis was performed. The interaction energy ∆*E*^‡^_int_ of **^3^TS3** for the tertiary C–H amination is stronger than that of **^3^TS4** for the benzylic C–H amination by 2.8 kcal mol^−1^. The distortion/interaction analysis of the transition states in the hydrogen atom abstraction step shows that site-selectivity is dominated by interaction energy. The IGM analysis indicates that the attractive π–π stacking interaction between the phenyl group on the substrate and the phthalimido group of the ligand in the tertiary C–H amination transition structure contributes to the formation of the tertiary C–H amination product.

## Data Availability

Not applicable.

## Supplementary Materials

The following supporting information can be downloaded at: https://www.mdpi.com/article/10.3390/molecules28041928/s1, Figure S1: The phthaloyl face and *tert*-butyl face of Rh_2_(*S*-tfpttl)_4_ **1**; Figure S2: Optimized geometries and energies of conformers for the triplet state dirhodium catalyst; Figure S3: The dirhodium–nitrene formation process; Table S1: The energies and the <S^2^> value of the Rh–nitrene intermediate **2**; Table S2: Calculated spin densities of selected atoms in the optimized structures for Rh–nitrene **2**; Figure S4: The binding of nitrene to the *tert*-butyl face; Figure S5: The IRC pathway for **^1^TS2**; Figure S6: Free energy profiles of Rh_2_(*S*-tfpttl)_4_-catalyzed intermolecular C–H aminations; Table S3: The calculated spin densities for selected atoms of the species in tertiary and benzylic C–H aminations; Figure S7: Optimized geometries of **^3^TS3′** and **^3^TS4′**; Figure S8: The interaction energy for the C_6_H_6_-phthalimido complex with the eclipsed face-face orientation; Figure S9: Optimized geometries of **^3^TS7**, **^3^TS8**, **^3^TS3-*C1*** and **^3^TS4-*C1***; Figure S10: The two-layer ONIOM approach; Figure S11: Optimized geometries and energies of conformers for **2**; Figure S12: Optimized geometries and energies of conformers for **Sub**; Figure S13: The schematic of the dirhodium–nitrene: substrate adducts displaying the substrate fitting in the catalytic pocket; Figure S14: Optimized geometries and energies of conformers for **^1^TS1**; Figure S15: Optimized geometries and energies of conformers for **^1^TS2**; Figure S16: Optimized geometries and energies of conformers for **^3^TS3**; Figure S17: Optimized geometries and energies of conformers for **^3^TS4**; Figure S18: Optimized geometries and energies of conformers for **^3^TS7** and **^3^TS8**; Table S4: The energies of the key transition states using different functionals; Figure S19: Optimized geometries of **^3^TS3** and **^3^TS4** with different functionals; Table S5: Energies and free energies of the calculated structures; Cartesian coordinates of the structures.

## Sample Availability

Samples of the compounds are not available from the authors.
