# Supplementary material for: Computational Exploration of Dirhodium Complex-Catalyzed Selective Intermolecular Amination of Tertiary vs. Benzylic C−H Bonds
Source: Molecules. 2023 Feb 17;28(4):1928. doi: 10.3390/molecules28041928 (PMC9959850; doi:10.3390/molecules28041928)
Supplement: Supplementary file 1 [file molecules-28-01928-s001.zip › molecules-2180798-SI.pdf]

Supplementary Materials

Computational Exploration of Dirhodium Complex-Catalyzed Selective Intermolecular Amination of Tertiary *vs.* Benzylic C−H Bonds

Xingxing Su, Xiahe Chen, Debo Ding, Yuan-Bin She* and Yun-Fang Yang*

College of Chemical Engineering, Zhejiang University of Technology, Hangzhou, Zhejiang 310014, China

*E-mail: yangyf@zjut.edu.cn

*E-mail: sheyb@zjut.edu.cn

Table of Contents

The phthaloyl face and *tert*-butyl face of Rh_2(*S*-tfpttl)$_4$ **1**. S2

Optimized geometries and energies of conformers for triplet state dirhodium catalyst. S2

The dirhodium–nitrene formation process. S3

The energies and the <S^2> value of the Rh–nitrene intermediate **2**. S4

Calculated spin densities of selected atoms in the optimized structures for Rh–nitrene **2**. S5

The binding of the nitrene to the *tert*-butyl face. S5

The IRC pathway for the ^{1}**TS2**. S6

Free energy profiles of Rh_2(*S*-tfpttl)$_4$-catalyzed intermolecular C–H aminations. S7

The calculated spin densities for selected atoms of the species in tertiary and benzylic C–H aminations. S7

Optimized geometries of ^{3}**TS3’** and ^{3}**TS4’**. S8

The interaction energy for the C_6H_6-phthalimido complex with the eclipsed face-face orientation. S9

Optimized geometries of ^{3}**TS7**, ^{3}**TS8**, ^{3}**TS3-*C1*** and ^{3}**TS4-*C1***. S9

The two-layer ONIOM approach. S10

Optimized geometries and energies of conformers for **2**. S10

Optimized geometries and energies of conformers for **Sub**. S11

The schematic of the dirhodium–nitrene: substrate adducts displaying the substrate fitting in the catalytic pocket. S11

Optimized geometries and energies of conformers for ^{1}**TS1**. S12

Optimized geometries and energies of conformers for ^{1}**TS2**. S13

Optimized geometries and energies of conformers for ^{3}**TS3**. S14

Optimized geometries and energies of conformers for ^{3}**TS4**. S15

Optimized geometries and energies of conformers for ^{3}**TS7** and ^{3}**TS8**. S16

The energies of the key transition states using different functionals. S17

Optimized geometries of ^{3}**TS3** and ^{3}**TS4** with different functionals. S17

Energies and free energies of the calculated structures. S18

Cartesian coordinates of the structures. S19

1. Results

1.1 The dirhodium complex Rh_2(S-tfpttl)$_4$

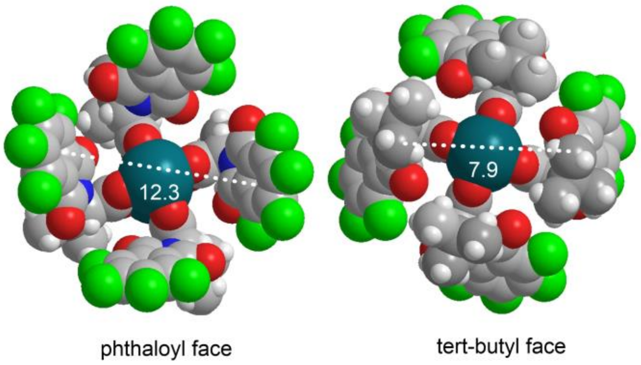

Figure S1. The phthaloyl face and *tert*-butyl face of Rh_2(*S*-tfpttl)$_4$ **1**. The pocket diameters are labeled. The distances are shown in Å.

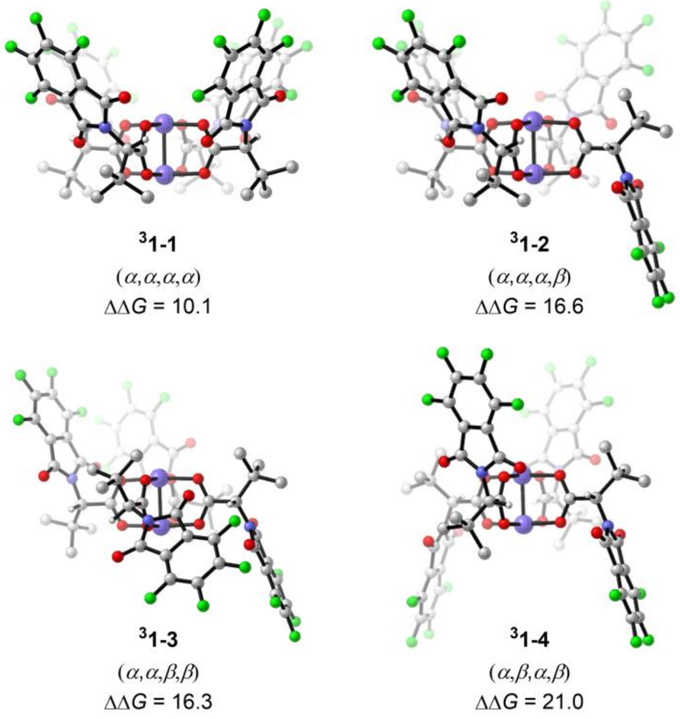

Figure S2. Optimized geometries and energies of triplet state dirhodium catalyst conformers. Free energy obtained at the M06/def2-TZVP//BPW91/6-31G*-LANL2DZ level. Energies are shown in kcal mol^{-1}. The H atoms of ^{t}Bu groups and aryl groups were omitted for clarity.

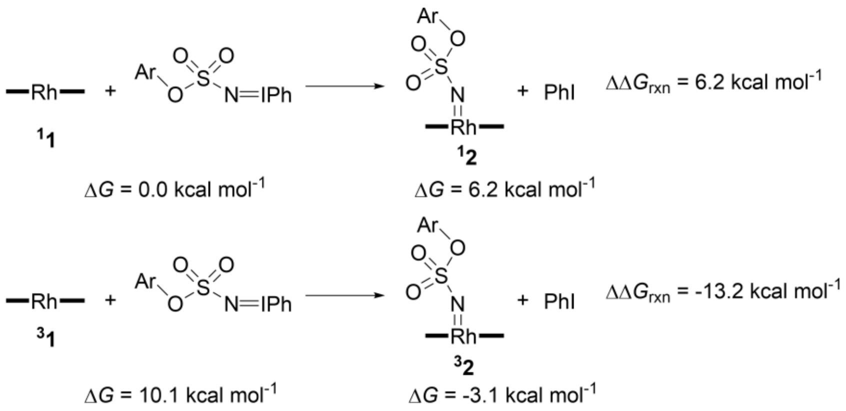

Figure S3. The energetics for the formation of dirhodium–nitrene.

1.2 The Dirhodium–nitrene complex

The singlet ^{1}**2** was first optimized by the restricted DFT method. Further analysis performed with the *stable=opt* keyword suggested the instability of the restricted wave function. The reoptimization was conducted with the Gaussian keyword *guess=read* to achieve an unrestricted and symmetry-broken solution accordingly. The resulting structure ^{1}**2** was obtained with $\langle S^2 \rangle$ of 0.2728 before annihilation of the spin contamination, and $\langle S^2 \rangle$ of 0.0019 after annihilation. The structure ^{1}**2** can be described as closed shell with small spin polarization due to effects of the unrestricted approach. In addition, we have done additional calculations for the high-level (HL) layer of structure ^{1}**2** using other functionals (Table S2). The energy ordering of the closed-shell singlet, open-shell singlet and triplet Rh–nitrene intermediate **2** obtained from these different functionals (BPW91, M06, MN15 and *w*B97xD) are the same, albeit the numbers of each structure differ. The result at the level of ONIOM(M06:UFF//BPW91:UFF) indicates that the energies of the open-shell and closed-shell are close to a degeneracy. The value of $<S^2>$ in the open-shell singlet state is affected by different functionals.

Table S1. The energies and the $<S^2>$ value of the Rh–nitrene intermediate **2** at different levels of theory. The energies are shown in kcal mol^{-1} ($\Delta\Delta E$).

Computational Level	Triplet	Closed-shell singlet	Open-shell singlet
	$\Delta\Delta E$<br>$<S^2>$	$\Delta\Delta E$<br>$<S^2>$	$\Delta\Delta E$<br>$<S^2>$
ONIOM(M06/def2-TZVP:UFF<br>//BPW91/6-31G*-LANL2DZ:UFF)	0.0<br><2.0001>	9.9<br><0.0000>	9.6<br><0.0019>
ONIOM(M06/def2-TZVP:UFF<br>//M06/6-31G*-LANL2DZ:UFF)	0.0<br><2.0005>	7.0<br><0.0000>	5.2<br><0.2308>
ONIOM(M06/def2-TZVP:UFF<br>//MN15/6-31G*-LANL2DZ:UFF)	0.0<br><2.0002>	6.8<br><0.0000>	5.1<br><0.0431>
ONIOM(M06/def2-TZVP:UFF<br>//*w*B97xD/6-31G*-LANL2DZ:UFF)	0.0<br><2.0007>	6.5<br><0.0000>	6.0<br><0.6409>

Table S2. Calculated spin densities of selected atoms in Rh–nitrene **2** at the level of ONIOM(M06:UFF//BPW91:UFF).

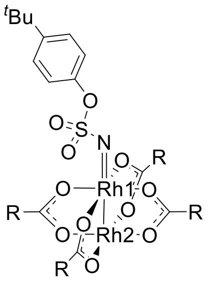

Structure	Rh1–Rh2	N
**^{1}2**	0.040	-0.072
**^{3}2**	0.718	0.960

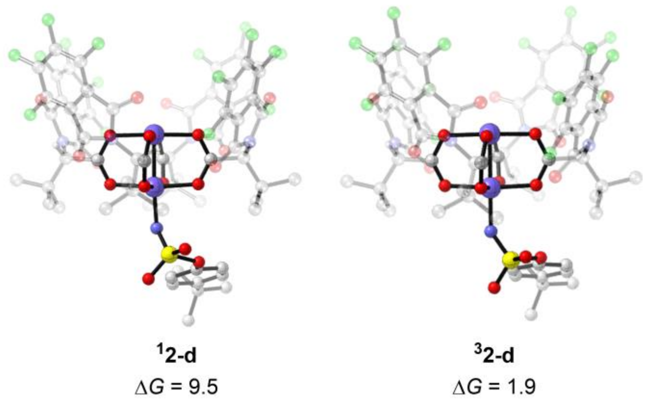

Figure S4. The binding of the nitrene to the *tert*-butyl face of the catalyst. Free energy obtained at the ONIOM(M06/def2-TZVP:UFF//BPW91/LANL2DZ-6-31G*:UFF) level. Energies are shown in kcal mol^{-1}. The H atoms of ^{t}Bu groups and aryl groups were omitted for clarity.

1.3 Singlet pathway

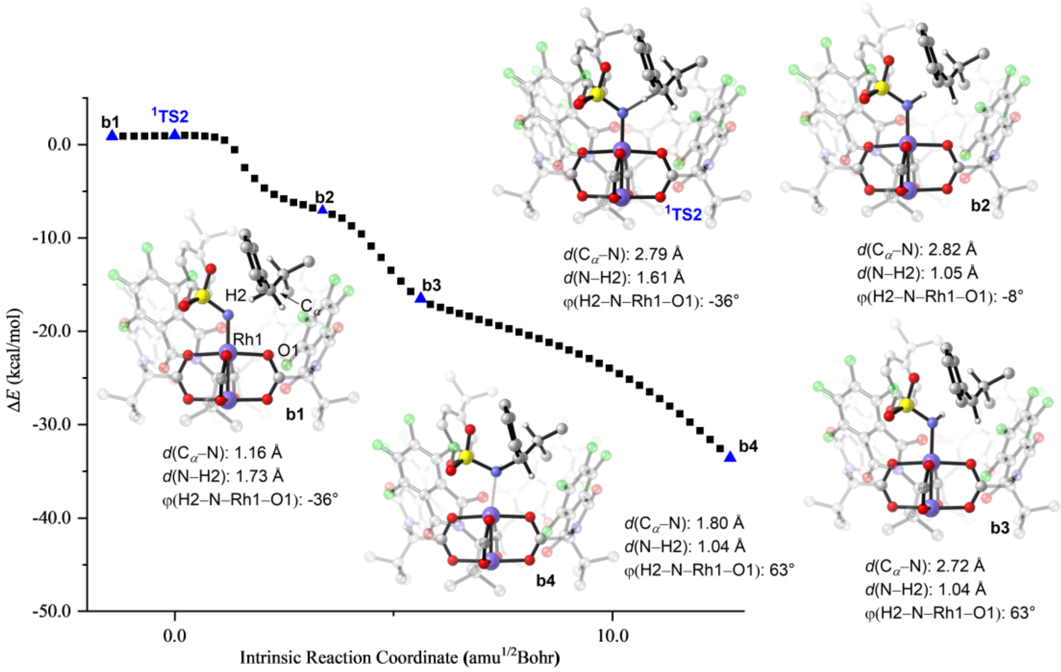

Figure S5. The IRC path for **^{1}TS2**. The H atoms of ^{t}Bu groups and aryl groups were omitted for clarity.

1.4 Triplet pathway

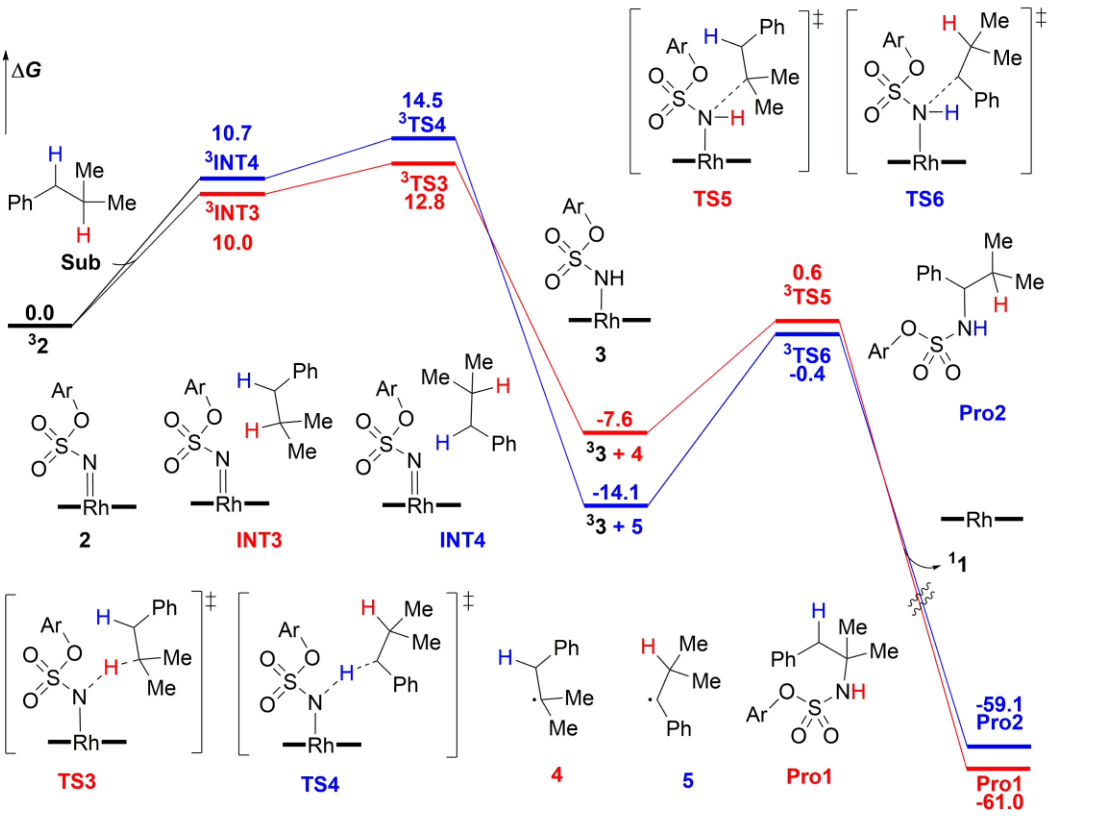

Figure S6. Free energy profiles of the triplet pathway. Free energy obtained at the ONIOM(M06/def2-TZVP:UFF//BPW91/LANL2DZ-6-31G*:UFF) level. Energies are shown in kcal mol^{-1}. Ar = *p-tert*-butylphenyl.

Table S3. The calculated spin densities on selected atoms in tertiary and benzylic C–H aminations at the level of ONIOM(M06:UFF//BPW91:UFF).

Structures		The Spin Density Distribution					
		N	Rh1–Rh2	C_α	C_β	$PhCH_2$	Ph
Tertiary	^{3}**INT3**	0.924	0.708	0.001	0.002	0.020	0.019
	^{3}**TS3**	0.702	0.575	-0.016	0.452	0.036	0.008
	^{3}**INT5**	0.350	0.596	-0.061	**0.827**	0.023	**0.007**
	^{3}**TS5**	0.011	1.412	-0.031	0.389	0.002	0.000
Benzylic	^{3}**INT4**	0.899	0.693	0.028	0.004	0.083	0.059
	^{3}**TS4**	0.714	0.563	0.320	-0.009	-	0.188
	^{3}**INT6**	0.366	0.559	**0.678**	-0.047	-	**0.287**
	^{3}**TS6**	0.052	1.372	0.274	-0.019	-	0.113

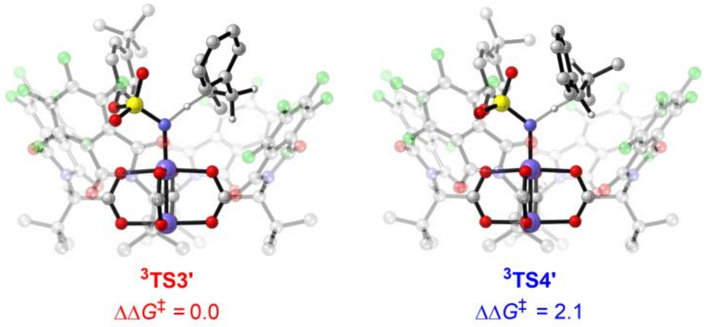

Figure S7. Optimized geometries of selective transition state **^{3}TS3'** and **^{3}TS4'**. Free energy obtained at the M06/def2-TZVP//BPW91/LANL2DZ-6-31G* level. Energies are shown in kcal mol^{-1}. The H atoms of ^{t}Bu groups and aryl groups were omitted for clarity.

1.5 Origins of site-selectivity

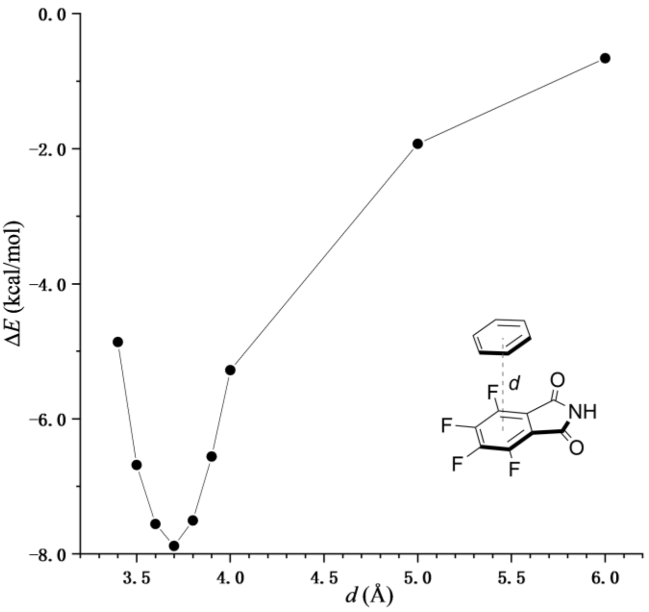

Figure S8. The interaction energy for the C_6H_6-phthalimido complex with the eclipsed face-face orientation at different distances between the centers of mass.

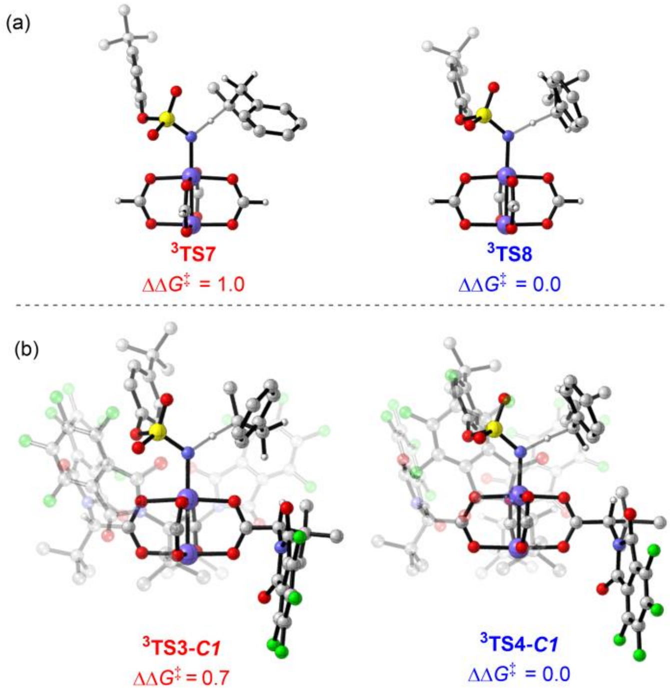

Figure S9. (a) The triplet transition states for $Rh_2(O_2CH)_4$-catalyzed intermolecular amination of tertiary and benzylic C–H bonds. Free energy obtained at the M06/def2-TZVP//BPW91/LANL2DZ-6-31G* level. (b) The triplet transition states for the amination of tertiary and benzylic C–H bonds with the dirhodium catalyst moiety adopting the *α*,*α*,*α*,*β* conformer. Free energy obtained at the ONIOM(M06/def2-TZVP:UFF//BPW91/LANL2DZ-6-31G*:UFF) level. The H atoms of ^{t}Bu groups and aryl groups were omitted for clarity. The energies are shown in kcal mol^{-1}.

2. Computational detail

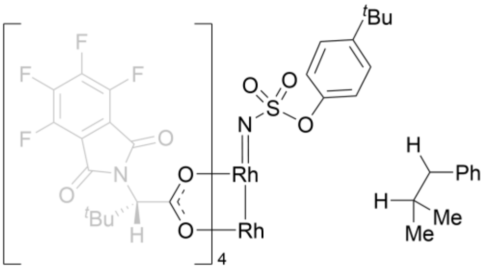

Figure S10. The two-layer ONIOM approach. The ligand part (gray) was calculated by the force field (UFF), and the other parts (black) were calculated by BPW91 or M06 method.

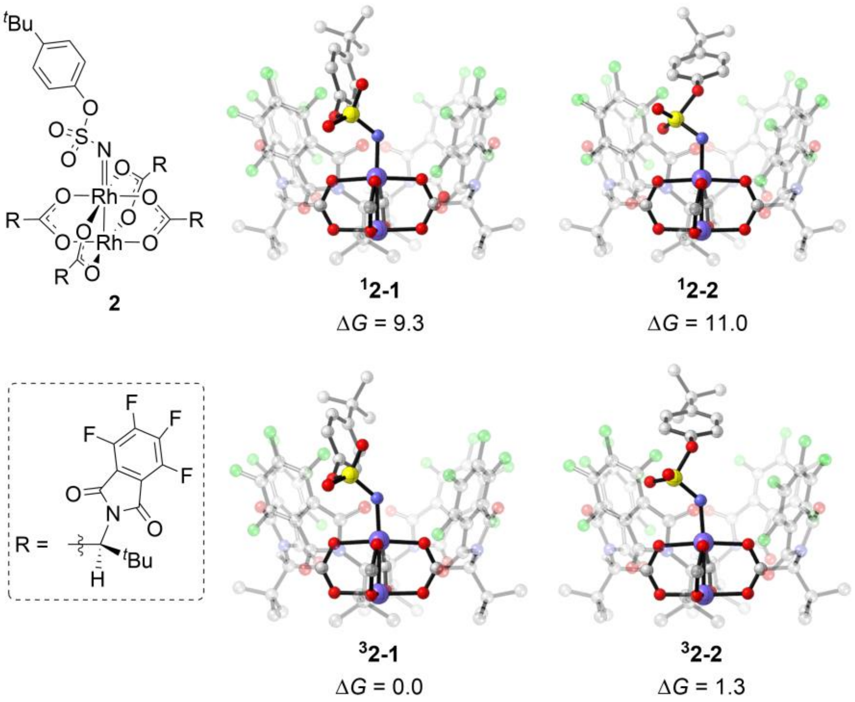

Figure S11. Optimized geometries and energies of conformers for Rh_2(*S*-tfpttl)$_4$–nitrene **2**. Free energy obtained at the ONIOM(M06/def2-TZVP:UFF//BPW91/LANL2DZ-6-31G*:UFF) level. The energies are shown in kcal mol^{-1}. The H atoms of ^{t}Bu groups and aryl groups were omitted for clarity.

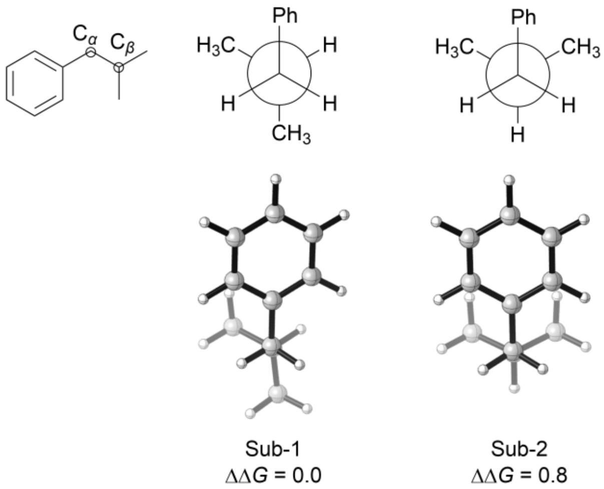

Figure S12. Newman projection along the C −C_β bond of the substrate isobutylbenzene. Optimized geometries and energies of conformers for the substrate. The energies are shown in kcal mol^{-1}. Free energy obtained at the M06/def2-TZVP//BPW91/LANL2DZ-6-31G* level.

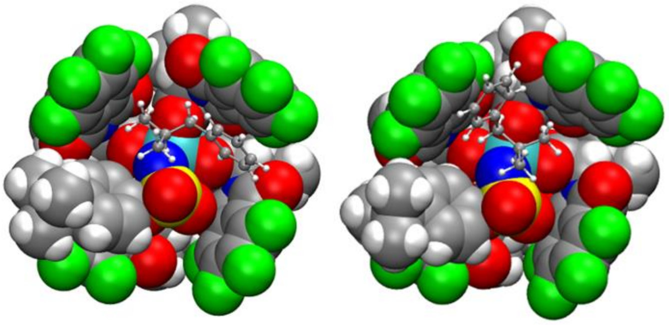

Figure S13. The schematic of the dirhodium−nitrene with substrate adducts displaying the substrate fitting in the catalytic pocket. The dirhodium−nitrene is represented with Van der Waals spheres mimicking the real volume of the molecule.

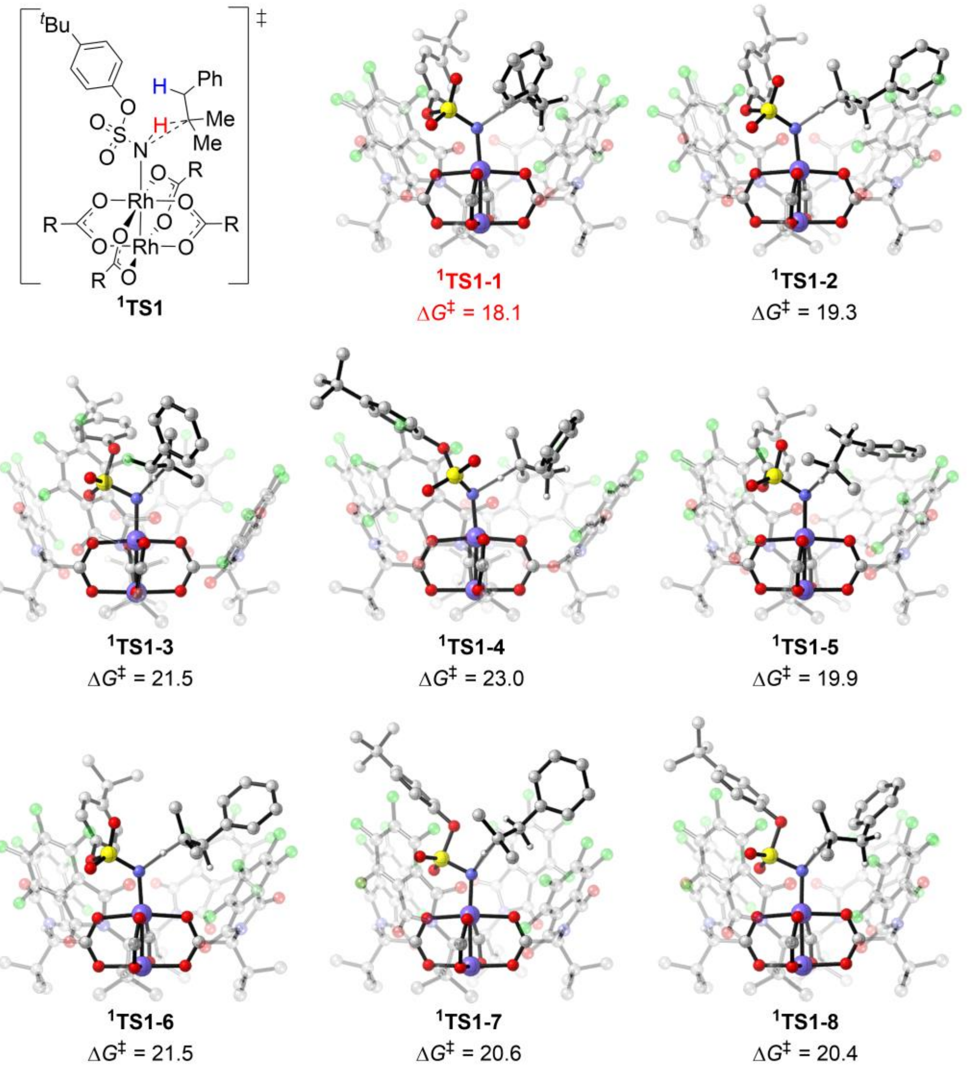

Figure S14. Optimized geometries and energies of conformers for **^{1}TS1**. Free energy obtained at the ONIOM(M06/def2-TZVP:UFF//BPW91/LANL2DZ-6-31G*:UFF) level. The energies are shown in kcal mol^{-1}. The H atoms of ^{t}Bu groups and aryl groups were omitted for clarity.

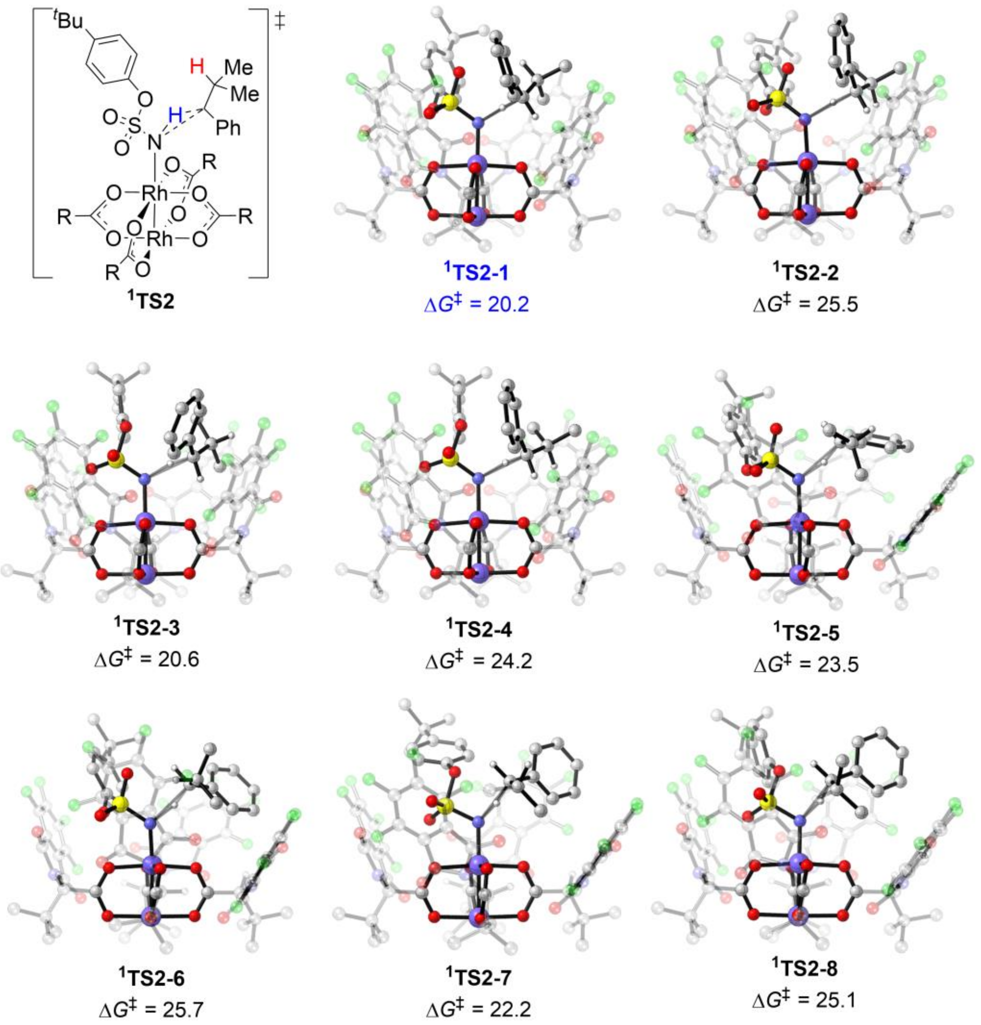

Figure S15. Optimized geometries and energies of conformers for **^{1}TS2**. Free energy obtained at the ONIOM(M06/def2-TZVP:UFF//BPW91/LANL2DZ-6-31G*:UFF) level. Energies are shown in kcal mol^{-1}. The H atoms of ^{t}Bu groups and aryl groups were omitted for clarity.

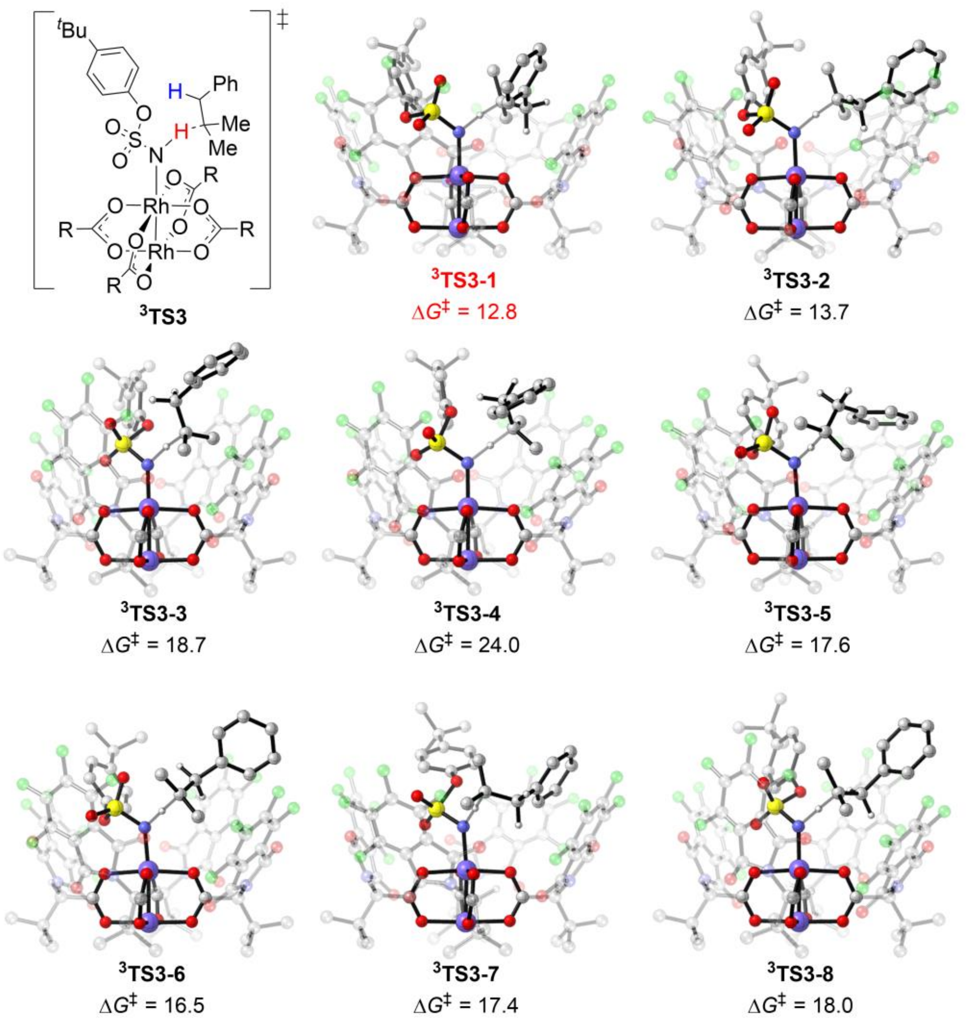

Figure S16. Optimized geometries and energies of conformers for ^{3}**TS3**. Free energy obtained at the ONIOM(M06/def2-TZVP:UFF//BPW91/LANL2DZ-6-31G*:UFF) level. Energies are shown in kcal mol^{-1}. The H atoms of ^{t}Bu groups and aryl groups were omitted for clarity.

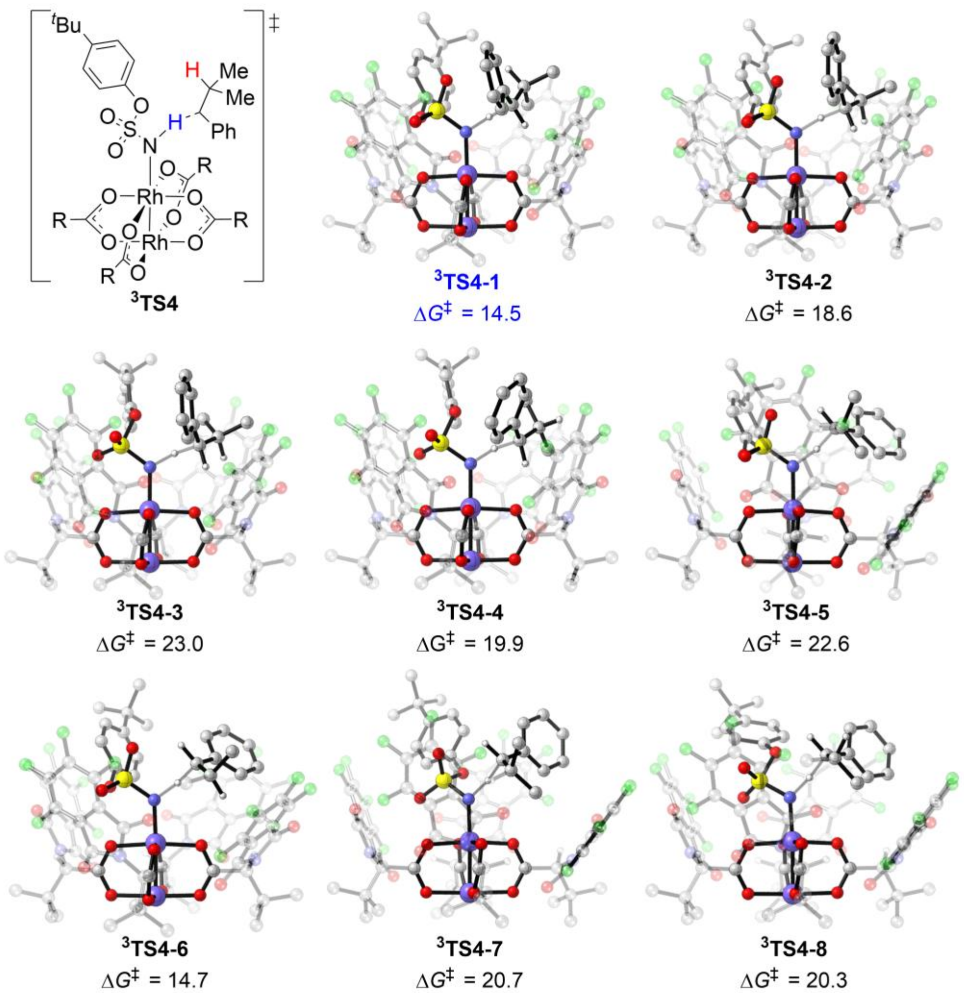

Figure S17. Optimized geometries and energies of conformers for **^{3}TS4**. Free energy obtained at the ONIOM(M06/def2-TZVP:UFF//BPW91/LANL2DZ-6-31G*:UFF) level. Energies are shown in kcal mol^{-1}. The H atoms of ^{t}Bu groups and aryl groups were omitted for clarity.

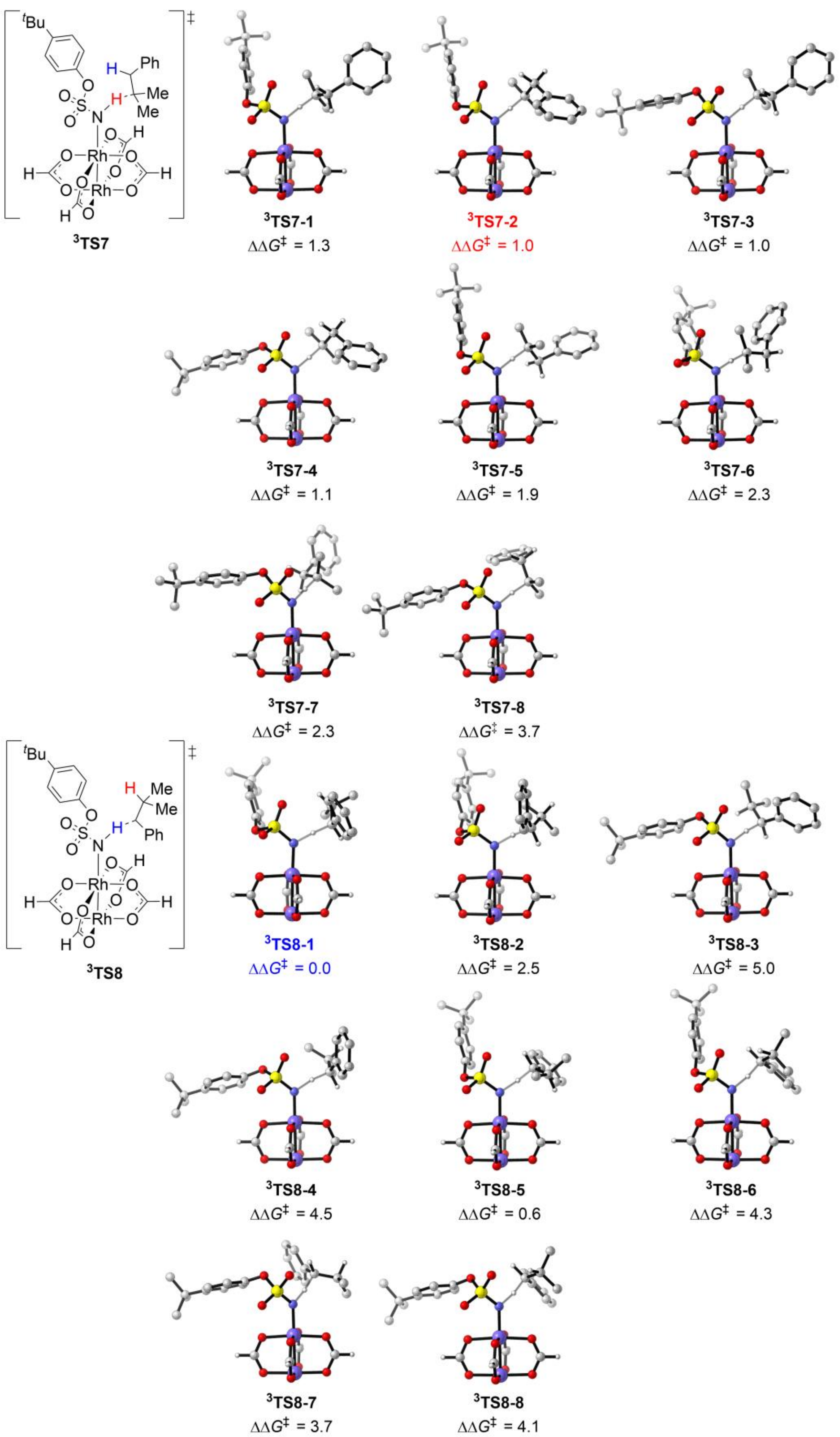

Figure S18. Optimized geometries and energies of conformers for **^{3}TS7** and **^{3}TS8**. Free energy obtained at the M06/def2-TZVP//BPW91/6-31G*-LANL2DZ level. Energies are shown in kcal mol^{-1}. The H atoms of ^{t}Bu groups and aryl groups were omitted for clarity.

Table S4. The energies of the key transition states using different functionals. The energies are shown in kcal mol^{-1} ($\Delta\Delta G^{\ddagger}$).

Methods	$\Delta\Delta G^{\ddagger a}$
ONIOM(M06/def2-TZVP:UFF //BPW91/6-31G*-LANL2DZ:UFF)	1.7
ONIOM(M06/def2-TZVP:UFF //M06/6-31G*-LANL2DZ:UFF)	4.5
ONIOM(M06/def2-TZVP:UFF //M06L-D3/6-31G*-LANL2DZ:UFF)	3.3
ONIOM(M06/def2-TZVP:UFF //MN15/6-31G*-LANL2DZ:UFF)	2.1
ONIOM(M06/def2-TZVP:UFF //*w*B97xD/6-31G*-LANL2DZ:UFF)	2.3

[a] $\Delta\Delta G^{\ddagger} = \Delta G^{\ddagger}_{\mathbf{TS4}} - \Delta G^{\ddagger}_{\mathbf{TS3}}$

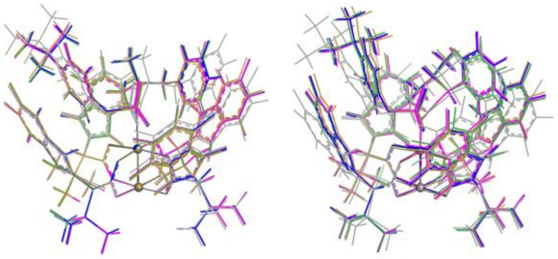

^{3}**TS3** ^{3}**TS4**

Figure S19. Optimized geometries of ^{3}**TS3** and ^{3}**TS4**. The geometry optimization of the HL layer was performed using the different functional: BPW91 (in gray), M06 (in green), M06L-D3 (in blue), MN15 (in yellow), and (in pink).

3. Gibbs free energies of calculated structures

Table S5. Electronic energies and free energies (in Hartree) of the structures. Calculated at the ONIOM(M06/def2-TZVP:UFF)//ONIOM(BPW91/LANL2DZ-6-31G*:UFF) Level of Theory.

Structures	ZPVE	TCE	TCH	TCG	E_{gas}	$G_{gas}(E_{gas}$+TCG)	Imaginary Frequency	G_{gas} (kcal/mol)
**^{1}1-1**[a]	0.894214	0.986519	0.987464	0.75623	-5398.797569	-5398.041339	—	—
**^{1}1-2**[a]	0.894181	0.98649	0.987434	0.754601	-5398.786705	-5398.032104	—	—
**^{1}1-3**[a]	0.894187	0.986531	0.987476	0.75545	-5398.786705	-5398.031255	—	—
**^{1}1-4**[a]	0.893878	0.986333	0.987277	0.75197	-5398.775497	-5398.023527	—	—
**^{1}1**	1.002308	1.079837	1.080782	0.888513	-977.8683429	-976.9798299	—	—
**sub**[a]	0.208972	0.219148	0.220092	0.173041	-389.3207502	-389.1477092	—	—
**^{1}2**	1.208686	1.305487	1.306431	1.068665	-2045.071219	-2044.002554	—	9.3
**^{3}2**	1.209174	1.305688	1.306632	1.069031	-2045.086477	-2044.017446	—	0.0
**^{1}INT1**	1.41776	1.52718	1.52812	1.26272	-2434.400419	-2433.137699	—	17.2
**^{1}TS1**	1.41513	1.522833	1.523777	1.265236	-2434.401473	-2433.136237	219.34*i*	18.1
**^{1}INT2**	1.418077	1.527185	1.528129	1.265146	-2434.404217	-2433.139071	—	16.4
**^{3}TS2**	1.416598	1.524228	1.525172	1.267428	-2434.400364	-2433.132936	75.61*i*	20.2
**^{3}INT3**	1.418362	1.527529	1.528473	1.262134	-2434.411415	-2433.149281	—	10.0
**^{3}TS3**	1.413047	1.521227	1.522171	1.261212	-2434.406012	-2433.1448	1161.60*i*	12.8
**^{3}INT4**	1.418103	1.527187	1.528132	1.263066	-2434.41118	-2433.148114		10.7
**^{3}TS4**	1.41387	1.521866	1.52281	1.262522	-2434.40457	-2433.142048	997.54*i*	14.5
**^{3}INT5**	1.416554	1.525723	1.526667	1.262432	-2434.427453	-2433.165021	—	0.1
**^{3}TS5**	1.416999	1.525384	1.526328	1.265082	-2434.429218	-2433.164136	206.02*i*	0.6
**^{3}INT6**	1.417638	1.526616	1.52756	1.262959	-2434.435401	-2433.172442	—	-4.6
**^{3}TS6**	1.417834	1.526425	1.527369	1.265186	-2434.430965	-2433.165779	240.32*i*	-0.4
**Pro1**[a]	0.428276	0.454255	0.455200	0.370183	-1456.632071	-1456.261888	—	-59.1
**Pro2**[a]	0.428359	0.454582	0.455526	0.369437	-1456.62817	-1456.258733	—	-61.0

ZPVE = zero-point vibrational energy; TCE = thermal correction to energy; TCH = thermal correction to enthalpy; TCG = thermal correction to Gibbs free energy.

[a]Calculated at the M06/def2-TZVP//BPW91/LANL2DZ-6-31G* Level of Theory.

4. Cartesian coordinates of the structures

M06/def2-TZVP//BPW91/LANL2DZ-6-31G*

^{1}1-1

C	5.63807800	2.54911400	1.50234200
C	-4.30307300	-4.71383900	2.78349300
C	-4.71722900	4.29756400	2.78482700
C	2.55483400	-5.63483700	1.50739200
C	2.12776100	4.55764400	-2.80629900
C	-4.55879700	2.12756300	-2.80565600
C	-2.12839000	-4.55874400	-2.80523600
C	4.55790800	-2.12719100	-2.80659200
C	-4.79135500	0.84431700	-3.64049700
C	2.70242600	5.94251300	-2.41962000
C	5.94272200	-2.70209600	-2.42006600
C	-3.17273500	-3.79829800	-3.64984000
C	3.17307500	3.79758800	-3.65003800
C	3.79740800	-3.17247900	-3.64996400
C	-3.79827100	3.17232900	-3.64965600
C	-2.70435600	-5.94295400	-2.41818800
C	-5.94324200	2.70307100	-2.41870400
C	4.78967500	-0.84401300	-3.64179000
C	-0.84473900	-4.79182000	-3.63931800
C	0.84434200	4.78917700	-3.64115300
C	-1.13502700	-2.39122200	-1.56115400
C	2.39069800	-1.13437400	-1.56159400
C	1.13412300	2.39057900	-1.56166200
C	-2.39144800	1.13407200	-1.56133200
C	-1.73034500	-3.81878400	-1.47820400
C	3.81872100	-1.72856400	-1.47930400
C	1.72956200	3.81808800	-1.47913400
C	-3.81918200	1.72893400	-1.47861800
C	-3.80644200	-2.84104400	-0.34849400
C	4.62997000	-2.70152700	0.70248100
C	2.70530900	4.62702300	0.70236200
C	-2.84218600	3.80403800	-0.34634200
C	-4.52231800	-3.14707600	0.92789100
C	4.22450900	-3.85579700	1.56149700
C	3.86029500	4.22008900	1.55975200
C	-3.14919400	4.51869400	0.93051000
C	-3.86114900	-4.22017100	1.56074400

C	3.15132500	-4.51853100	0.93037800
C	4.52136500	3.14673300	0.92723700
C	-4.22268400	3.85685100	1.56199200
C	-2.70616500	-4.62732500	0.70345000
C	2.84357900	-3.80437600	-0.34661400
C	3.80534100	2.84022700	-0.34896200
C	-4.62907300	2.70261400	0.70335500
C	-5.63907800	-2.54936600	1.50279800
C	4.71948500	-4.29569900	2.78444000
C	4.30228500	4.71414700	2.78232100
C	-2.55188200	5.63485800	1.50697500
C	-6.09505800	-3.04618700	2.74403200
C	4.12015100	-5.42816500	3.37822300
C	5.43511100	4.11364800	3.37422500
C	-3.04959200	6.08962000	2.74828600
C	-5.43593600	-4.11322900	3.37522000
C	3.05301300	-6.08880300	2.74881600
C	6.09412600	3.04635100	2.74338500
C	-4.11705400	5.42986500	3.37809000
F	-6.29727800	-1.53366300	0.93436700
F	5.73433600	-3.69665700	3.42078600
F	3.70489400	5.72912800	3.42000500
F	-1.53575900	6.29362500	0.93995200
F	-7.16838000	-2.49986200	3.33100000
F	4.57024700	-5.88309000	4.55620600
F	5.89200700	4.56281000	4.55180200
F	-2.50366600	7.16235900	3.33669100
F	-5.89275200	-4.56201500	4.55297200
F	2.50781800	-7.16167900	3.33766600
F	7.16743000	2.50015800	3.33051000
F	-4.56667700	5.88552800	4.55597400
F	-3.70560500	-5.72855500	3.42151500
F	1.53901900	-6.29450100	0.94084400
F	6.29623100	1.53321900	0.93423700
F	-5.73236800	3.69942000	3.42155800
H	6.52648800	-2.89664700	-3.33484700
H	-3.38858900	-4.37558700	-4.56517400
H	-5.40787100	1.08899900	-4.52169500
H	2.89686600	6.52643300	-3.33433000
H	5.85422200	-3.65674200	-1.87611100
H	3.65712000	5.85411200	-1.87572900
H	-2.80739100	-2.80576000	-3.94766100
H	-5.32495900	0.07508000	-3.05792900
H	6.51723000	-2.00170800	-1.79195600

H	2.00198500	6.51682400	-1.79139200
H	-4.12122000	-3.66409800	-3.10804900
H	-3.84572500	0.41126700	-3.99356400
H	4.37417000	-3.38861800	-4.56555900
H	3.38891900	4.37461900	-4.56553700
H	-4.37520100	3.38820300	-4.56521400
H	-2.89885900	-6.52710500	-3.33273700
H	2.80451700	-2.80772900	-3.94738800
H	2.80857600	2.80464700	-3.94756500
H	-2.80551100	2.80728200	-3.94713300
H	-3.65922000	-5.85353300	-1.87476400
H	3.66392000	-4.12063000	-3.10744900
H	4.12138700	3.66431500	-3.10772100
H	-3.66453400	4.12071300	-3.10760200
H	-2.00467300	-6.51754200	-1.78938100
H	1.08856700	5.40563200	-4.52252200
H	5.40560100	-1.08869900	-4.52340200
H	-6.52724000	2.89785400	-3.33329200
H	-1.08902400	-5.40861500	-4.52043100
H	0.07478300	5.32244000	-3.05869300
H	5.32363200	-0.07465300	-3.05968800
H	-0.07594200	-5.32542600	-3.05614100
H	-5.85416700	3.65770800	-1.87482600
H	0.41189400	3.84318100	-3.99398100
H	-6.51783900	2.00296500	-1.79035800
H	-0.41129700	-3.84641900	-3.99250800
H	3.84373700	-0.41114000	-3.99423100
H	-0.92560600	-4.42016600	-1.01493900
H	4.41990800	-0.92288100	-1.01738500
H	0.92483500	4.41969900	-1.01613300
H	-4.42051900	0.92364000	-1.01623900
N	-2.78026200	-3.80370500	-0.44806700
N	3.80543000	-2.77756900	-0.44821500
N	2.77940700	3.80314000	-0.44893900
N	-3.80488100	2.77803300	-0.44765100
O	-0.91064800	-1.85065200	-0.41877400
O	1.85020000	-0.91041400	-0.41908900
O	0.91010500	1.85017500	-0.41911300
O	-1.85084300	0.90992800	-0.41888600
O	-0.86169000	-1.88573400	-2.70337100
O	1.88491800	-0.86098600	-2.70367300
O	0.86048700	1.88486900	-2.70370700
O	-1.88595500	0.86054500	-2.70347900
O	-4.02513300	-1.94342900	-1.15327300

O	5.47765500	-1.84489600	0.91043500
O	1.84977800	5.47547700	0.91165400
O	-1.94376600	4.02337600	-1.15003800
O	-1.85054300	-5.47559800	0.91302100
O	1.94519700	-4.02449600	-1.15011000
O	4.02361600	1.94204800	-1.15321900
O	-5.47738400	1.84667300	0.91154600
Rh	-0.00033200	-0.00022800	-0.37039100
Rh	-0.00058900	-0.00037400	-2.75622500

[1]1-2

C	1.46716400	6.28129500	1.17167800
C	4.94605000	-5.11363500	1.95302100
C	-8.11723600	-0.26409400	0.07246100
C	6.41056900	-0.37175000	-1.54746300
C	-3.75676100	3.99703300	-0.37772300
C	-3.46779300	-3.21454100	2.61281300
C	1.07394400	-4.53265000	-2.57075100
C	1.99916800	2.57735000	-4.05385200
C	-2.35125500	-2.95491200	3.65474300
C	-4.33602400	5.25061500	0.32242600
C	3.14123100	3.53501600	-4.47425000
C	-0.29908700	-5.11476500	-2.17271100
C	-3.22597100	4.39796700	-1.77026000
C	2.03566100	1.32583100	-4.95685900
C	-3.10695600	-4.47409900	1.79847100
C	2.09432100	-5.68859500	-2.71022000
C	-4.79501200	-3.45052400	3.37339000
C	0.65675100	3.32487400	-4.24635700
C	0.97041600	-3.82808700	-3.94645700
C	-4.89949500	2.96263700	-0.52735700
C	0.74364300	-2.31624600	-1.10108900
C	1.17255900	1.31303800	-1.82954500
C	-1.89815200	2.11384800	0.11093700
C	-2.46891300	-1.41524500	0.88096200
C	1.64322100	-3.50453700	-1.51989500
C	2.17858500	2.26337900	-2.52387100
C	-2.65801400	3.38720300	0.56468100
C	-3.65779600	-1.91107300	1.73834200
C	1.48806000	-4.49983700	0.83721200
C	4.41843000	2.72390400	-1.46045100
C	-1.68811100	4.82786800	2.39167800
C	-5.25824900	-2.58432700	-0.20574400
C	2.51585700	-4.94917700	1.82854900

C	5.64459600	1.91585800	-1.18339400
C	-0.45802300	5.66285500	2.53855200
C	-6.57425300	-2.01385000	-0.64295500
C	3.79124900	-4.78092500	1.25266200
C	5.42490500	0.60437500	-1.65448400
C	0.26513100	5.59891700	1.32883700
C	-6.93291100	-0.97547000	0.23810400
C	3.61176800	-4.21538400	-0.11921200
C	4.04291400	0.53568400	-2.22474300
C	-0.48155200	4.71748800	0.38031700
C	-5.85101600	-0.83933400	1.25447400
C	2.37103700	-5.45404200	3.11627400
C	6.84809000	2.27258000	-0.58481200
C	0.01060000	6.40651100	3.61635400
C	-7.39705500	-2.36486100	-1.70822400
C	3.53777500	-5.79558800	3.83458000
C	7.85123500	1.28617900	-0.46436800
C	1.23151500	7.10057600	3.46690800
C	-8.60155100	-1.64930700	-1.88499000
C	4.80818200	-5.62789200	3.26063000
C	7.63635400	-0.01657900	-0.94102700
C	1.94940400	7.03951700	2.26182200
C	-8.95773600	-0.61359600	-1.00600200
F	1.18030300	-5.62941500	3.70370700
F	7.09755500	3.50443900	-0.12187200
F	-0.64113900	6.49411300	4.78238800
F	-7.09947000	-3.34330900	-2.57239400
F	3.43952500	-6.28546100	5.07841600
F	9.02541600	1.59143400	0.10624500
F	1.71549300	7.82886400	4.48263000
F	-9.42002500	-1.95631000	-2.90131000
F	5.89711700	-5.96065500	3.96818300
F	8.61137100	-0.92775800	-0.81931100
F	3.10238500	7.71250100	2.15133800
F	-10.10817400	0.04669300	-1.20143700
F	6.17679500	-4.96791100	1.44525900
F	6.25540200	-1.61965200	-1.99799700
F	2.17898200	6.25454500	0.04074900
F	-8.49232400	0.73126900	0.88635600
H	2.98353900	3.85635200	-5.51699800
H	-0.63209300	-5.82509100	-2.94847000
H	-2.32300100	-3.79903300	4.36404900
H	-5.17929400	5.64348600	-0.26909300
H	4.12776000	3.04557500	-4.42649600

H	-3.58825600	6.05600100	0.40575100
H	-1.06389000	-4.33043600	-2.08115100
H	-2.53310600	-2.03322000	4.23329300
H	3.17468400	4.43699700	-3.84142200
H	-4.70755800	5.02183300	1.33470300
H	-0.25297700	-5.65781000	-1.21699500
H	-1.36197000	-2.87797700	3.18275000
H	1.88626500	1.63291200	-6.00624400
H	-4.05649100	4.80546500	-2.37174000
H	-2.96244100	-5.31881300	2.49405400
H	1.77263200	-6.36121600	-3.52223800
H	1.24385200	0.61164800	-4.69190000
H	-2.80288100	3.53708300	-2.30597700
H	-2.16860000	-4.34404300	1.24197600
H	2.16243500	-6.29254400	-1.79077400
H	3.00141400	0.80187100	-4.89696000
H	-2.44882200	5.17425500	-1.70466400
H	-3.89436400	-4.74893500	1.08446300
H	3.10320400	-5.31823600	-2.95542000
H	-5.73148300	3.41693100	-1.09095800
H	0.56860300	3.65062400	-5.29641400
H	-4.67107500	-4.29549300	4.07044600
H	0.65895200	-4.56151200	-4.70899800
H	-5.29461100	2.64915000	0.45420600
H	0.59923900	4.22167000	-3.60784700
H	1.94368300	-3.41253800	-4.25909300
H	-5.62237500	-3.70429700	2.69129300
H	-4.56689400	2.06795100	-1.07172500
H	-5.09317100	-2.56694700	3.96312700
H	0.23459600	-3.01319800	-3.93418300
H	-0.20376200	2.68305700	-4.01391200
H	2.52039500	-3.02413500	-1.99563700
H	2.04565400	3.22420800	-1.99188900
H	-3.18282100	3.08128300	1.48997800
H	-3.80105800	-1.09618800	2.47202400
N	2.21155900	-4.11870800	-0.31214100
N	3.53599400	1.84962100	-2.14243200
N	-1.66482500	4.35258500	1.05480700
N	-4.90264800	-1.85150600	0.95799200
O	1.08167600	-1.71727400	-0.01838000
O	1.41915100	1.09685800	-0.58770800
O	-0.99361300	1.72270500	0.93245900
O	-1.40863100	-1.08257100	1.52014300
O	-0.20233400	-1.96798900	-1.88972800

O	0.16604000	0.87673800	-2.48595200
O	-2.24631100	1.52147500	-0.96826900
O	-2.66790800	-1.26818600	-0.37632500
O	0.27395400	-4.44810300	0.97166000
O	4.18235500	3.89032300	-1.17824100
O	-2.55584300	4.57401800	3.21475100
O	-4.63080100	-3.48978400	-0.73063000
O	4.46380900	-3.88733100	-0.93281700
O	3.43979200	-0.43529300	-2.66482700
O	-0.16237500	4.35485400	-0.74583800
O	-5.75328000	-0.02580800	2.16601400
Rh	0.05630900	-0.00882100	0.50369500
Rh	-1.25878900	-0.23480100	-1.47419300

[1]1-3

C	-6.87710000	1.95180900	-0.69164000
C	7.20049000	-2.84193200	0.80893400
C	-6.95266200	-3.00861700	0.76526000
C	6.60358400	2.68791300	-1.31055600
C	-3.21288600	1.44012600	3.89848400
C	-1.18956400	-4.91081500	0.38911100
C	3.20035300	-1.70409200	-3.56744000
C	1.21325800	4.94644500	-1.00767800
C	-0.09780500	-5.12405800	1.46541500
C	-4.49442200	2.14962600	4.39449100
C	1.94369900	6.28488400	-0.73833700
C	2.20171800	-2.85986200	-3.78776000
C	-3.41627800	-0.08865800	3.94779400
C	1.34024100	4.60183300	-2.50718000
C	-0.52904300	-4.89325000	-1.00405800
C	4.59909900	-2.12800500	-4.07713600
C	-2.20336600	-6.07393000	0.48072800
C	-0.27766600	5.12743500	-0.63205200
C	2.75084100	-0.46847200	-4.38769600
C	-2.05055100	1.83691200	4.84308800
C	2.07420100	-0.93692100	-1.24916500
C	1.20742200	2.44298800	-0.04326400
C	-1.83791300	1.18800800	1.62246100
C	-1.15413600	-2.27223400	0.47065900
C	3.34930300	-1.27123800	-2.05900500
C	1.82565500	3.86555700	-0.04745100
C	-2.87416800	1.98211400	2.44929200
C	-1.95002400	-3.56654600	0.73722700
C	3.86446800	-3.36825800	-0.66526200

C	4.12661400	4.28561700	0.89573200
C	-4.26205300	3.66709800	1.23755900
C	-3.79331400	-3.37061700	-1.10968300
C	5.03405000	-3.76851200	0.17872900
C	5.51781200	3.91617600	0.49770900
C	-5.42694500	3.63481900	0.30637200
C	-5.27515300	-3.17674600	-0.99722900
C	6.00098400	-2.74565200	0.11139100
C	5.45042400	3.14903300	-0.68465800
C	-5.81044300	2.28949800	0.13377400
C	-5.63074800	-3.16980900	0.36440500
C	5.47148400	-1.66505600	-0.77259300
C	4.01196600	2.97507800	-1.05082800
C	-4.90640500	1.42550900	0.95979300
C	-4.38774600	-3.33884900	1.16615900
C	5.24887900	-4.90923200	0.94481100
C	6.74041200	4.21890400	1.08752500
C	-6.09818700	4.66565100	-0.34346600
C	-6.23868900	-3.02882300	-1.98931900
C	6.46456900	-5.01666500	1.65537600
C	7.91606100	3.74268500	0.46658500
C	-7.18097700	4.32962700	-1.18484400
C	-7.58337100	-2.86328800	-1.59345500
C	7.42759000	-3.99692700	1.58830800
C	7.84893600	2.99095200	-0.71708400
C	-7.56484300	2.98997300	-1.35701100
C	-7.93594700	-2.85457100	-0.23451800
F	4.35962600	-5.90567900	1.03900900
F	6.85180300	4.94081500	2.20970900
F	-5.76495800	5.95475300	-0.20629800
F	-5.95026300	-3.02880800	-3.29769300
F	6.70890200	-6.10081100	2.40430100
F	9.11341800	4.01464300	1.00437500
F	-7.85317800	5.29302500	-1.82932100
F	-8.54127600	-2.71209500	-2.52019400
F	8.57132800	-4.12805500	2.27468000
F	8.98365200	2.56642300	-1.28855800
F	-8.59610500	2.70116100	-2.16222100
F	-9.22293400	-2.69435000	0.10848000
F	8.14488400	-1.89250900	0.77396000
F	6.58709200	1.99227700	-2.45084900
F	-7.28054300	0.68817900	-0.88643600
F	-7.33139300	-2.99368800	2.04969400
H	1.46970400	7.08617000	-1.32872500

H	2.14582000	-3.09046500	-4.86530400
H	0.43568800	-6.06726600	1.26304700
H	-4.66281100	1.89405000	5.45351900
H	3.00408600	6.24029400	-1.03608400
H	-5.38769400	1.83212400	3.83346500
H	1.19088800	-2.58889800	-3.44927100
H	-0.53321100	-5.18941300	2.47757200
H	1.89914400	6.57326300	0.32464100
H	-4.40988500	3.24745900	4.32409500
H	2.50478800	-3.77453600	-3.25862400
H	0.64406100	-4.31274900	1.45987600
H	0.87208100	5.40441800	-3.10248900
H	-3.67252600	-0.39060900	4.97719400
H	-0.04549900	-5.86703700	-1.19020200
H	4.55022800	-2.31554000	-5.16232700
H	0.84296500	3.65352300	-2.75054100
H	-2.49885800	-0.62554300	3.66382200
H	0.25907400	-4.12829900	-1.06582600
H	4.95138800	-3.05557100	-3.59749800
H	2.39200900	4.52606200	-2.82262900
H	-4.22577100	-0.41847500	3.28273800
H	-1.26104000	-4.70354700	-1.79996300
H	5.35276400	-1.34136800	-3.90441200
H	-2.27476600	1.49350200	5.86668200
H	-0.70814700	5.95440800	-1.22045500
H	-1.66567700	-7.03205700	0.38884300
H	2.73299800	-0.73069900	-5.45887600
H	-1.91748400	2.93243200	4.87968800
H	-0.40126500	5.38437500	0.43418700
H	3.44472100	0.37830700	-4.25655600
H	-2.94942500	-6.03579100	-0.32867200
H	-1.10046200	1.38223200	4.52994600
H	-2.73627900	-6.07858400	1.44688400
H	1.74510000	-0.13395000	-4.10004100
H	-0.86487600	4.22223300	-0.84123800
H	3.89829500	-0.30910700	-2.07603800
H	1.65962700	4.23671800	0.98054200
H	-2.40103400	2.97136900	2.60365300
H	-2.10589000	-3.58264800	1.82942500
N	4.21984200	-2.12918300	-1.24280000
N	3.28843700	3.74181700	-0.11405700
N	-4.02686800	2.31970800	1.60929200
N	-3.32422900	-3.44537900	0.22739100
O	2.21710700	-0.95269500	0.02732400

O	1.58675100	1.71156000	0.94229000
O	-0.82740600	0.72705500	2.25928900
O	-0.26370700	-1.97675000	1.34919300
O	1.02366800	-0.57663900	-1.88325700
O	0.35872000	2.11824500	-0.94209400
O	-2.02552500	1.12804900	0.35538800
O	-1.45540200	-1.57776500	-0.56103200
O	2.81205500	-3.96812700	-0.83194700
O	3.73874200	4.91767300	1.86783200
O	-3.61123900	4.62815100	1.62769900
O	-3.13899900	-3.47122000	-2.13624400
O	5.97873700	-0.58658200	-1.05214500
O	3.52172400	2.29945100	-1.94831000
O	-4.92109200	0.20754800	1.06952400
O	-4.25929900	-3.39450700	2.38250500
Rh	0.70965300	-0.15380100	1.17907000
Rh	-0.54748800	0.25685800	-0.80830300

^{1}1-4

C	2.54649000	4.81578500	4.72452300
C	-5.82498000	4.11684700	-2.81530800
C	5.82808000	-3.90896100	-3.08301900
C	-2.52382500	-5.11325300	4.41178900
C	3.45015400	-0.93411300	3.72728600
C	3.20156400	1.51157800	-3.79670300
C	-3.23140300	-1.26282700	-3.86589500
C	-3.45016000	0.68468200	3.78428300
C	3.41422100	2.86701100	-3.07661100
C	4.60743500	-0.48535700	4.65071600
C	-4.60712500	0.17591500	4.67662500
C	-1.94609600	-1.27680300	-4.71905300
C	2.20604600	-1.26253000	4.57811400
C	-2.20694900	0.95994300	4.65527100
C	1.90801100	1.57070900	-4.63512600
C	-4.45136900	-0.96417100	-4.76898900
C	4.41381900	1.27256300	-4.72749800
C	-3.91330500	2.00117800	3.11087900
C	-3.44337300	-2.65804000	-3.22597700
C	3.91126100	-2.20530600	2.97053400
C	-1.93486600	-0.10545800	-1.82989800
C	-1.93102100	-0.23768600	1.77996200
C	1.93102300	0.11826400	1.78841300
C	1.92644400	0.22811400	-1.82467100
C	-3.20435500	-0.18653700	-2.70768700

C	-3.19868800	-0.38877800	2.65184400
C	3.20055500	0.21016400	2.66579400
C	3.18951000	0.36799600	-2.70437900
C	-2.89443300	2.11707300	-3.82864500
C	-4.40154900	-2.57496000	2.52812800
C	4.40749100	2.39718700	2.67438300
C	2.88377700	-1.86570500	-3.96102500
C	-3.71801000	3.36813500	-3.79241100
C	-4.17664800	-3.95722600	3.04365400
C	4.18867000	3.74475700	3.27684400
C	3.71281500	-3.11300100	-4.00322700
C	-4.87193900	3.12435500	-3.02069600
C	-2.99261300	-3.95119100	3.80830400
C	3.00921800	3.69288100	4.04677000
C	4.86970000	-2.91039600	-3.22412900
C	-4.81250600	1.71573000	-2.53224600
C	-2.43416500	-2.56101000	3.81266500
C	2.44772300	2.30613900	3.96742600
C	4.80669900	-1.53377900	-2.65210200
C	-3.49610500	4.61106200	-4.37572300
C	-4.91401000	-5.12351600	2.86595900
C	4.92739600	4.91860000	3.16777600
C	3.49318800	-4.31996600	-4.65850600
C	-4.45753300	5.62523400	-4.17262100
C	-4.44461400	-6.30746700	3.47500000
C	4.46404800	6.06301500	3.85231100
C	4.45995100	-5.34005100	-4.52090800
C	-5.60679000	5.38146700	-3.40359300
C	-3.26511800	-6.30233800	4.23681900
C	3.28913800	6.01224700	4.61951700
C	5.61221100	-5.13727300	-3.74451100
F	-2.41537800	4.88982700	-5.11556600
F	-6.04263200	-5.17252200	2.14742200
F	6.05177100	5.01044500	2.44678600
F	2.40979400	-4.55918000	-5.40823200
F	-4.27786500	6.83696900	-4.71690600
F	-5.12568400	-7.45261900	3.32772500
F	5.14645500	7.21405300	3.77251700
F	4.28257200	-6.51806000	-5.13547400
F	-6.50070700	6.36506600	-3.22948900
F	-2.84426200	-7.44307900	4.80116100
F	2.87393800	7.11625900	5.25644800
F	6.51120600	-6.12541800	-3.63376000
F	-6.93358000	3.92673900	-2.08914100

F	-1.40452900	-5.15650200	5.14555900
F	1.43171700	4.81511100	5.46640500
F	6.93968400	-3.75735500	-2.35239400
H	-4.89155100	0.96815800	5.38849700
H	-2.04118300	-2.04124300	-5.50878000
H	3.47413000	3.67396300	-3.82582100
H	4.89095200	-1.32241100	5.30969900
H	-4.31710200	-0.70842400	5.26605400
H	4.31800700	0.35909300	5.29623400
H	-1.05957300	-1.52886500	-4.11918100
H	4.35586400	2.87448300	-2.50011500
H	-5.50151900	-0.07991300	4.08340800
H	5.50222200	-0.19248000	4.07549300
H	-1.76462900	-0.30533600	-5.19941700
H	2.58623000	3.09668100	-2.39195400
H	-2.45895800	1.71154400	5.42256000
H	2.45726100	-2.05934000	5.29863500
H	1.99339100	2.37933300	-5.38070600
H	-4.57070000	-1.78158600	-5.49900500
H	-1.37144800	1.35830300	4.06172100
H	1.37155900	-1.62438500	3.96029900
H	1.02690600	1.78565800	-4.01328800
H	-4.32617100	-0.02944500	-5.33832500
H	-1.85546400	0.05208100	5.16492500
H	1.85354900	-0.38724400	5.14115700
H	1.72413900	0.62766600	-5.16823300
H	-5.38609900	-0.89453400	-4.18694700
H	4.14238800	-3.00073000	3.69853200
H	-4.14765800	2.74697200	3.88867500
H	4.52346700	2.13296400	-5.40795400
H	-3.51532500	-3.41835400	-4.02147700
H	4.82540400	-2.01491600	2.38128000
H	-4.82606500	1.84730100	2.50897700
H	-4.37902200	-2.69582600	-2.64097500
H	4.28610700	0.37342600	-5.35099500
H	3.13203000	-2.57845700	2.29185700
H	5.35434800	1.17155000	-4.15955600
H	-2.60953800	-2.93219900	-2.56523000
H	-3.13406400	2.41969300	2.45929000
H	-3.99970500	-0.49134200	-1.99877400
H	-4.03287500	-0.26441900	1.93282600
H	4.03315400	0.12926200	1.93879000
H	3.98867800	0.63445500	-1.98453200
N	-3.62883300	1.16510800	-3.08444000

N	-3.34849000	-1.78905100	3.05934400
N	3.35544200	1.58144300	3.16089400
N	3.61759000	-0.95661400	-3.16424700
O	-1.61940200	1.05421800	-1.38264500
O	-1.55730500	0.95167600	1.48498300
O	1.41126000	1.23077000	1.41832400
O	1.35102500	1.31015900	-1.45076000
O	-1.35692900	-1.20982400	-1.53304700
O	-1.41324700	-1.32260600	1.33324800
O	1.55734100	-1.04855400	1.41390500
O	1.61281700	-0.95884100	-1.45452400
O	-1.82105300	1.93710500	-4.38461200
O	-5.28909000	-2.15910000	1.79462000
O	5.29022400	2.02642000	1.91148700
O	1.80731900	-1.65802600	-4.50114200
O	-5.59314100	1.11752500	-1.80315300
O	-1.41367500	-2.15924500	4.35182200
O	1.43001200	1.87347800	4.48764600
O	5.58872200	-0.97660700	-1.89263800
Rh	-0.10752800	1.18810600	0.01942100
Rh	0.10439200	-1.18630300	-0.06134900

Sub

C	-0.38664000	-0.12955900	-0.47716800
C	-1.12513700	1.06232500	-0.61374400
C	-2.46861200	1.13306200	-0.21446200
C	-3.10122200	0.00650000	0.33058900
C	-2.38035900	-1.18993700	0.47042400
C	-1.03902800	-1.25455200	0.06910500
H	-0.64060800	1.94506100	-1.04395100
H	-3.02174400	2.06960900	-0.33341000
H	-4.14918200	0.05779200	0.63975200
H	-2.86618000	-2.07717900	0.88764100
H	-0.48809000	-2.19549600	0.17410100
C	1.07091300	-0.21031600	-0.89170000
H	1.31255200	0.63048700	-1.56810000
H	1.23069400	-1.13520500	-1.47659500
C	2.08200400	-0.20358500	0.28977100
H	1.79463800	-1.02605400	0.97339300
C	3.50563000	-0.48630400	-0.22305000
H	3.83766700	0.30149100	-0.92250300
H	4.22928700	-0.51807900	0.60889800
H	3.56078400	-1.45120200	-0.75555800
C	2.03110100	1.11199900	1.08525600

H	2.32472000	1.96725600	0.45025000
H	1.01952500	1.31211000	1.47298300
H	2.72403700	1.08159800	1.94296700

Pro1

N	-2.33485600	-0.86122900	-0.61481100
S	-1.37519500	-2.23152600	-0.47579900
O	-2.19670000	-3.34491800	0.00796500
O	-0.30176000	-1.95670800	0.84136200
O	-0.56808500	-2.28750500	-1.70223700
C	-1.22543700	3.29359900	-1.50442000
C	-2.19289200	2.27896000	-1.43614200
C	-2.90187700	2.02646100	-0.24109300
C	-2.62603500	2.84017900	0.87851900
C	-1.66404400	3.85785700	0.81215900
C	-0.95609300	4.08462100	-0.37870000
H	-0.69133400	3.47068500	-2.44218900
H	-2.41858400	1.68701300	-2.32988300
H	-3.18371800	2.68339800	1.80692100
H	-1.47276700	4.48090200	1.69073800
H	-0.20822500	4.88073800	-0.43113200
C	-3.93436500	0.91649100	-0.17810800
H	-4.75874100	1.21127000	0.49333300
H	-4.37315800	0.77046600	-1.17972400
C	-3.44137200	-0.48708700	0.32541000
H	-1.73530900	-0.06466700	-0.86572600
C	-2.98985600	-0.43214300	1.79785100
H	-2.17084400	0.28755100	1.94609500
H	-3.83453000	-0.13134800	2.44022500
H	-2.64279000	-1.42005900	2.13447900
C	-4.61209000	-1.47618900	0.15796000
H	-4.90328000	-1.55471600	-0.90119000
H	-4.34110400	-2.47813900	0.51606000
H	-5.48088100	-1.12018800	0.73597600
C	0.93865700	-1.36304600	0.57250300
C	1.15348400	-0.03055900	0.94354500
C	1.98509500	-2.11946400	0.02218600
C	2.42228500	0.54328600	0.76675200
H	0.33814200	0.54893900	1.38223000
C	3.23964900	-1.52807600	-0.15109400
H	1.80732900	-3.15736300	-0.26444700
C	3.49351200	-0.18593200	0.21489100
H	2.56293600	1.58226700	1.07070400
H	4.04108700	-2.13510200	-0.58153500

C	4.89829900	0.41643500	0.00618800
C	4.97998700	1.88941700	0.46289800
C	5.93518600	-0.40101900	0.82132900
C	5.26523800	0.35606000	-1.50033300
H	4.28434300	2.53378800	-0.10043500
H	4.76050300	1.99989200	1.53808700
H	5.99903600	2.27445200	0.29235200
H	5.95842400	-1.45854500	0.51302600
H	6.94834600	0.01223000	0.67674000
H	5.70340600	-0.37046100	1.89883900
H	6.27223300	0.77616300	-1.66663900
H	5.26691600	-0.67750900	-1.88186100
H	4.54806300	0.93532300	-2.10511700

Pro2

N	2.09312300	-0.69572500	-0.56578800
S	1.56574600	-2.24745400	-0.16488700
O	2.45296500	-3.23597600	-0.79223000
O	0.16363600	-2.41205500	-1.08537200
O	1.20516400	-2.26586600	1.26175800
C	-0.98335600	-1.69885600	-0.69136600
C	-1.76919000	-2.15062400	0.37841800
C	-1.37950500	-0.59308800	-1.44900600
C	-2.95554200	-1.47509700	0.68176700
H	-1.44844300	-3.01760100	0.95793900
C	-2.57755500	0.06520900	-1.13251800
H	-0.75761500	-0.26125700	-2.28221800
C	-3.39155200	-0.35443700	-0.06186600
H	-3.55731000	-1.84006100	1.51882300
H	-2.86827700	0.92377300	-1.74045500
C	-4.71151800	0.35107300	0.31264700
C	-4.61687300	0.87712100	1.76941800
H	-3.79466800	1.60419800	1.87376100
H	-5.55703500	1.37845900	2.05740200
H	-4.43809100	0.06255900	2.48951300
C	-5.88414500	-0.65945700	0.20524000
H	-6.83609200	-0.17191700	0.47770000
H	-5.97954200	-1.04606000	-0.82291100
H	-5.74642500	-1.52211600	0.87669000
C	-5.02114400	1.54826700	-0.61243600
H	-5.96999000	2.01834000	-0.30497300
H	-4.23662700	2.32164800	-0.56082100
H	-5.13227000	1.23733200	-1.66475100
C	1.83855600	1.67766500	0.13961400

C	1.56592300	2.08551100	-1.17835300
C	1.08748500	3.37757100	-1.44565700
C	0.87043100	4.28252500	-0.39703300
C	1.13116300	3.88411300	0.92307100
C	1.61036200	2.59373400	1.18582100
H	1.71039500	1.38206400	-2.00230200
H	0.88150000	3.67473200	-2.47823000
H	0.49487500	5.28835700	-0.60518900
H	0.95361500	4.57597200	1.75157500
H	1.80133800	2.28909400	2.22049500
C	2.41156400	0.30107800	0.48250900
H	2.77990900	-0.77341800	-1.31999100
H	1.90397900	-0.05860600	1.39178900
C	3.94390500	0.37146000	0.80248800
C	4.46486600	-0.93778600	1.42117500
H	4.43027100	-1.77041500	0.69903900
H	5.51480500	-0.81964300	1.73660800
H	3.87471500	-1.23548500	2.30270100
C	4.79516200	0.79362200	-0.40931300
H	4.80940700	0.01703500	-1.19610200
H	4.43648500	1.73365500	-0.85776500
H	5.84328300	0.94253300	-0.10311000
H	4.03567700	1.16972300	1.56252500

ONIOM(M06/def2-TZVP:UFF)//ONIOM(BPW91/LANL2DZ-6-31G*:UFF)

[1]1

C-C_R	0	-4.68140100	-3.58664100	1.16740600	L
C-C_R	0	3.04019700	5.04339800	2.81057400	L
C-C_R	0	5.05221500	-3.03178900	2.81362300	L
C-C_R	0	-3.58709300	4.67536500	1.17401500	L
C-C_3	0	-0.38804000	-4.96640900	-2.68556100	L
C-C_3	0	4.96425800	-0.38348100	-2.68925600	L
C-C_3	0	0.37984900	4.96998700	-2.68084000	L
C-C_3	0	-4.97207500	0.38450800	-2.67775100	L
C-C_3	0	4.72828400	0.91655500	-3.49481400	L
C-C_3	0	-0.45663000	-6.47862900	-2.36309100	L
C-C_3	0	-6.48373300	0.45323700	-2.35269700	L
C-C_3	0	1.59762700	4.61859500	-3.56684300	L
C-C_3	0	-1.60913400	-4.61409600	-3.56661500	L
C-C_3	0	-4.62154800	1.60445700	-3.56110400	L

C-C_3	0	4.61077400	-1.60228200	-3.57303000	L
C-C_3	0	0.45006500	6.48170900	-2.35642300	L
C-C_3	0	6.47653800	-0.45386500	-2.36747400	L
C-C_3	0	-4.73663000	-0.91460600	-3.48496700	L
C-C_3	0	-0.92114800	4.73494000	-3.48512300	L
C-C_3	0	0.90999400	-4.72985200	-3.49419400	L
C-C_R	0	0.18359000	2.64259100	-1.40671300	H
C-C_R	0	-2.64417500	0.18665500	-1.40490000	H
C-C_R	0	-0.18840300	-2.64068900	-1.40895600	H
C-C_R	0	2.63918200	-0.18508100	-1.41116900	H
C-C_3	0	0.35114600	4.16412200	-1.32740400	L
C-C_3	0	-4.16575200	0.35331400	-1.32466700	L
C-C_3	0	-0.35483200	-4.16244800	-1.33108700	L
C-C_3	0	4.16084600	-0.35268800	-1.33440900	L
C-C_R	0	2.81402400	3.86848700	-0.53715000	L
C-C_R	0	-5.07715100	1.45877300	0.82297100	L
C-C_R	0	-1.45756600	-5.07913500	0.81551700	L
C-C_R	0	3.86712100	-2.81443800	-0.54060800	L
C-C_R	0	3.48541700	4.04852900	0.74599900	L
C-C_R	0	-4.76370600	2.68482500	1.55307000	L
C-C_R	0	-2.68281200	-4.76852000	1.54786000	L
C-C_R	0	4.04995600	-3.48196300	0.74332000	L
C-C_R	0	2.68848500	4.75839000	1.54377000	L
C-C_R	0	-4.05298300	3.48277500	0.75637300	L
C-C_R	0	-3.48402000	-4.05428400	0.75419500	L
C-C_R	0	4.76054400	-2.68597600	1.54139200	L
C-C_R	0	1.46106800	5.07437000	0.81844000	L
C-C_R	0	-3.87086200	2.81401100	-0.52784900	L
C-C_R	0	-2.81428200	-3.86877800	-0.52974900	L
C-C_R	0	5.07423700	-1.45957500	0.81189500	L
C-C_R	0	4.68069100	3.58115100	1.16570600	L
C-C_R	0	-5.05739200	3.02585200	2.82237700	L
C-C_R	0	-3.02088800	-5.06796300	2.81519400	L
C-C_R	0	3.58752400	-4.67110000	1.16972500	L
C-C_R	0	5.08437700	3.85003400	2.43416400	L
C-C_R	0	-4.59966500	4.27139400	3.30489100	L
C-C_R	0	-4.26496700	-4.61035400	3.29080400	L
C-C_R	0	3.88838100	-5.05732000	2.49418600	L
C-C_R	0	4.29074600	4.57270500	3.26851100	L
C-C_R	0	-3.87634500	5.08232100	2.49431000	L
C-C_R	0	-5.09292700	-3.85562900	2.42919300	L
C-C_R	0	4.61553800	-4.22733400	3.28831400	L
F-F_	0	5.46945100	2.85356100	0.33526500	L
F-F_	0	-5.77194900	2.19543800	3.62237400	L

F-F_	0	-2.19182300	-5.78472000	3.61452900	L
F-F_	0	2.85571800	-5.47508600	0.35849300	L
F-F_	0	6.28329100	3.39039100	2.87240200	L
F-F_	0	-4.87850400	4.65065000	4.57695200	L
F-F_	0	-4.65919500	-4.88386300	4.55908400	L
F-F_	0	3.44757000	-6.24802200	2.97131300	L
F-F_	0	4.69280600	4.83596200	4.53688400	L
F-F_	0	-3.42653200	6.27808100	2.94990000	L
F-F_	0	-6.29219600	-3.39836800	2.86785100	L
F-F_	0	4.90141500	-4.59229300	4.56337500	L
F-F_	0	2.22236200	5.75528400	3.62562100	L
F-F_	0	-2.85773900	5.46941300	0.35075000	L
F-F_	0	-5.46494600	-2.85764100	0.33324600	L
F-F_	0	5.76851100	-2.19847300	3.60963000	L
H-H_	0	-7.09149100	0.42394400	-3.28321700	L
H-H_	0	1.54954900	5.17695800	-4.52677800	L
H-H_	0	5.36869200	0.93603500	-4.40325600	L
H-H_	0	-0.42866700	-7.08474900	-3.29472200	L
H-H_	0	-6.73652100	1.39286200	-1.81636400	L
H-H_	0	-1.39560100	-6.73220900	-1.82599200	L
H-H_	0	1.63999500	3.54058400	-3.81482900	L
H-H_	0	4.98343300	1.80764200	-2.88289300	L
H-H_	0	-6.78873900	-0.40394800	-1.71389400	L
H-H_	0	0.40132800	-6.78492100	-1.72594700	L
H-H_	0	2.54768500	4.90614300	-3.07159800	L
H-H_	0	3.67923000	1.01327800	-3.83649000	L
H-H_	0	-5.18055100	1.55852900	-4.52077100	L
H-H_	0	-1.56449900	-5.17119900	-4.52744700	L
H-H_	0	5.16748500	-1.55572600	-4.53399800	L
H-H_	0	0.41913400	7.08917900	-3.28707900	L
H-H_	0	-3.54371100	1.64764400	-3.80971500	L
H-H_	0	-1.65261300	-3.53576100	-3.81301500	L
H-H_	0	3.53231600	-1.64450800	-3.81908500	L
H-H_	0	1.39094700	6.73415500	-1.82213700	L
H-H_	0	-4.90889500	2.55327300	-3.06335800	L
H-H_	0	-2.55729700	-4.90242700	-3.06819000	L
H-H_	0	4.89874300	-2.55182200	-3.07704500	L
H-H_	0	-0.40560600	6.78738400	-1.71591500	L
H-H_	0	0.92801500	-5.37090700	-4.40220900	L
H-H_	0	-5.37928400	-0.93406900	-4.39182100	L
H-H_	0	7.08234300	-0.42429700	-3.29925900	L
H-H_	0	-0.94215400	5.37709800	-4.39229400	L
H-H_	0	1.80267500	-4.98373900	-2.88407800	L
H-H_	0	-4.98918000	-1.80649000	-2.87312800	L

H-H_	0	-1.81154300	4.98844700	-2.87151600	L
H-H_	0	6.72960500	-1.39418700	-1.83249600	L
H-H_	0	1.00504100	-3.68099600	-3.83692400	L
H-H_	0	6.78367700	0.40247500	-1.72855800	L
H-H_	0	-1.01780500	3.68650100	-3.82870900	L
H-H_	0	-3.68834200	-1.00985700	-3.82939000	L
H-H_	0	-0.55952000	4.51480000	-0.79317000	L
H-H_	0	-4.51573100	-0.55860300	-0.79221000	L
H-H_	0	0.55792500	-4.51336500	-0.80052300	L
H-H_	0	4.51257800	0.55867000	-0.80210700	L
N-N_R	0	1.51853300	4.48494600	-0.48679300	L
N-N_R	0	-4.48699600	1.51873500	-0.48152500	L
N-N_R	0	-1.51908100	-4.48518100	-0.48685200	L
N-N_R	0	4.48307200	-1.51887200	-0.49269300	L
O-O_R	0	0.03005900	2.05848000	-0.27349500	H
O-O_R	0	-2.05947400	0.03126300	-0.27223400	H
O-O_R	0	-0.03223300	-2.05780700	-0.27546700	H
O-O_R	0	2.05706300	-0.03070100	-0.27701200	H
O-O_R	0	0.22280300	2.05561700	-2.54082200	H
O-O_R	0	-2.05778200	0.22834600	-2.53921400	H
O-O_R	0	-0.23062000	-2.05240400	-2.54227700	H
O-O_R	0	2.05023300	-0.22519400	-2.54422100	H
O-O_R	0	3.12414000	2.97628400	-1.36924000	L
O-O_R	0	-5.43328100	0.38218800	1.37290800	L
O-O_R	0	-0.38042600	-5.43747000	1.36293100	L
O-O_R	0	2.97179600	-3.12539300	-1.36901000	L
O-O_R	0	0.38515300	5.42481700	1.37325600	L
O-O_R	0	-2.97874400	3.12689900	-1.35903500	L
O-O_R	0	-3.12886900	-2.97666400	-1.36032400	L
O-O_R	0	5.42913400	-0.38310700	1.36280900	L
Rh-	0	-0.00112900	0.00032600	-0.22198800	H
Rh-	0	-0.00389700	0.00162900	-2.59930900	H

^{1}2

C-C_R	0	5.52092500	1.65877700	2.60338500	L
C-C_R	0	-4.58103700	-3.79027400	0.61338200	L
C-C_R	0	-4.11437000	4.48406900	0.99372700	L
C-C_R	0	1.69643600	-5.54411000	0.99690800	L
C-C_3	0	3.49918900	4.70953100	-2.07835000	L
C-C_3	0	-2.64129900	2.35041600	-4.52449900	L
C-C_3	0	-0.49972900	-4.23391400	-3.87739400	L
C-C_3	0	5.76228600	-2.00003000	-1.46059300	L
C-C_3	0	-2.50311000	1.10223700	-5.42969000	L
C-C_3	0	3.93306000	6.08883300	-1.52690100	L

C-C_3	0	6.94092100	-2.63874000	-0.68743700	L
C-C_3	0	-1.20765100	-3.40939200	-4.97744100	L
C-C_3	0	4.76452900	3.98592100	-2.59421800	L
C-C_3	0	5.35472300	-2.97220400	-2.59174400	L
C-C_3	0	-1.63580200	3.43202200	-4.98259700	L
C-C_3	0	-1.16475600	-5.63047800	-3.82700000	L
C-C_3	0	-4.06938800	2.91039700	-4.73075900	L
C-C_3	0	6.28320900	-0.68339000	-2.08472800	L
C-C_3	0	0.98154900	-4.44679000	-4.27332800	L
C-C_3	0	2.54043800	4.96974800	-3.26506400	L
C-C_R	0	0.06505500	-2.20992000	-2.25002800	H
C-C_R	0	3.35566400	-0.97667400	-0.97501900	H
C-C_R	0	2.13842700	2.56595800	-1.29186600	H
C-C_R	0	-1.14957500	1.28584000	-2.60273800	H
C-C_3	0	-0.60306600	-3.57284300	-2.45145900	L
C-C_3	0	4.59325000	-1.70750700	-0.44624900	L
C-C_3	0	2.76210500	3.91859300	-0.93357800	L
C-C_3	0	-2.46410200	1.96380100	-3.00927500	L
C-C_R	0	-2.98252100	-2.51162500	-2.29535400	L
C-C_R	0	4.19061200	-3.09718800	1.68777400	L
C-C_R	0	3.31427300	4.26400400	1.56179300	L
C-C_R	0	-1.74762000	4.05343900	-1.63322700	L
C-C_R	0	-4.05301800	-2.56531400	-1.30317100	L
C-C_R	0	3.22511200	-4.12601800	2.06493600	L
C-C_R	0	4.03027100	3.47837600	2.56358000	L
C-C_R	0	-2.22306800	4.63279700	-0.38196500	L
C-C_R	0	-3.77725600	-3.53841600	-0.43529200	L
C-C_R	0	2.66245000	-4.60649300	0.95656700	L
C-C_R	0	4.77088600	2.56218200	1.93658100	L
C-C_R	0	-3.43450000	4.14357100	-0.12160600	L
C-C_R	0	-2.51831000	-4.16833000	-0.82148500	L
C-C_R	0	3.22916900	-3.90894700	-0.19333600	L
C-C_R	0	4.56725900	2.71093800	0.49858900	L
C-C_R	0	-3.79620100	3.21863000	-1.19256000	L
C-C_R	0	-5.15173100	-1.79025500	-1.17774800	L
C-C_R	0	2.85654300	-4.54732600	3.28985500	L
C-C_R	0	3.98384500	3.55781900	3.90586400	L
C-C_R	0	-1.61517900	5.48683600	0.46078700	L
C-C_R	0	-6.00311500	-2.01634600	-0.14347300	L
C-C_R	0	1.85176200	-5.53457800	3.38506900	L
C-C_R	0	4.75552300	2.64220700	4.64707900	L
C-C_R	0	-2.30911100	5.85925300	1.63308400	L
C-C_R	0	-5.74163300	-2.99933800	0.75836900	L
C-C_R	0	1.28165300	-6.02505700	2.25732400	L

C-C_R	0	5.53079100	1.68279500	3.95728700	L
C-C_R	0	-3.54630300	5.34908000	1.87344100	L
F-F_	0	-5.40204400	-0.79670700	-2.06738600	L
F-F_	0	3.42796000	-4.03345800	4.40767200	L
F-F_	0	3.21644500	4.48586600	4.53023700	L
F-F_	0	-0.37667200	5.97410200	0.19886100	L
F-F_	0	-7.11191500	-1.24753400	-0.00120000	L
F-F_	0	1.45492500	-5.98422600	4.60160800	L
F-F_	0	4.74902600	2.67629600	6.00263800	L
F-F_	0	-1.74065800	6.71891400	2.51486300	L
F-F_	0	-6.58478800	-3.21661300	1.79784800	L
F-F_	0	0.31076300	-6.96875800	2.33769600	L
F-F_	0	6.27663700	0.78654200	4.65024300	L
F-F_	0	-4.21540900	5.70260100	2.99950800	L
F-F_	0	-4.28870200	-4.76867700	1.50607000	L
F-F_	0	1.13202500	-6.01725300	-0.14205000	L
F-F_	0	6.25712400	0.73882400	1.93058700	L
F-F_	0	-5.34798200	3.97487700	1.23358400	L
H-H_	0	7.80770800	-2.81555500	-1.36086800	L
H-H_	0	-1.05220300	-3.88053100	-5.97211600	L
H-H_	0	-2.75887400	1.35164200	-6.48234700	L
H-H_	0	4.41744400	6.69884000	-2.32020900	L
H-H_	0	6.64971500	-3.61770800	-0.25002600	L
H-H_	0	4.66258600	5.97679600	-0.69639600	L
H-H_	0	-0.82261200	-2.37310900	-5.03843500	L
H-H_	0	-3.19000300	0.29629900	-5.09353700	L
H-H_	0	7.27562500	-1.97520300	0.13934400	L
H-H_	0	3.05564100	6.65415700	-1.14435100	L
H-H_	0	-2.30269800	-3.37069600	-4.80534100	L
H-H_	0	-1.47082600	0.70141300	-5.43787400	L
H-H_	0	6.19215600	-3.09844800	-3.31169500	L
H-H_	0	5.22344300	4.55999700	-3.42812300	L
H-H_	0	-1.78448300	3.66514700	-6.05918700	L
H-H_	0	-1.06567800	-6.15266000	-4.80350500	L
H-H_	0	4.48013200	-2.60729500	-3.16402300	L
H-H_	0	4.54143200	2.97129100	-2.97666400	L
H-H_	0	-0.58473800	3.10706300	-4.85765500	L
H-H_	0	-2.24908000	-5.55080100	-3.59828200	L
H-H_	0	5.12150100	-3.97881200	-2.18827800	L
H-H_	0	5.53041500	3.90386100	-1.79578200	L
H-H_	0	-1.78179000	4.37560900	-4.41705600	L
H-H_	0	-0.69072300	-6.26418100	-3.04623700	L
H-H_	0	3.02432300	5.62201100	-4.02411000	L
H-H_	0	7.17008500	-0.87764100	-2.72620000	L

H-H_	0	-4.25170000	3.14875800	-5.80125500	L
H-H_	0	1.05486300	-5.01750400	-5.22447900	L
H-H_	0	1.61693300	5.48035100	-2.91799400	L
H-H_	0	6.59017500	0.03107800	-1.29161900	L
H-H_	0	1.51784600	-5.02377400	-3.49008500	L
H-H_	0	-4.22225400	3.84482300	-4.14955600	L
H-H_	0	2.25042400	4.03327800	-3.78083300	L
H-H_	0	-4.83296600	2.17018500	-4.40697000	L
H-H_	0	1.51503900	-3.48921300	-4.43093600	L
H-H_	0	5.52167700	-0.19412200	-2.72326600	L
H-H_	0	-0.05066000	-4.25477000	-1.76860700	L
H-H_	0	5.02493300	-1.00900600	0.30422200	L
H-H_	0	1.89525700	4.55519100	-0.64738400	L
H-H_	0	-3.24579400	1.19965400	-2.79975100	L
N-N_R	0	-1.99919200	-3.50937900	-1.98318600	L
N-N_R	0	4.17297500	-2.92859500	0.26443100	L
N-N_R	0	3.62046300	3.76232500	0.25473300	L
N-N_R	0	-2.71877400	3.12178300	-2.13294500	L
O-O_R	0	0.01842200	-1.77643500	-1.04406400	H
O-O_R	0	2.51747200	-0.62941900	-0.06468400	H
O-O_R	0	1.40559400	2.05902800	-0.36847700	H
O-O_R	0	-1.10954700	0.88128900	-1.38511300	H
O-O_R	0	0.60240100	-1.60262600	-3.23412400	H
O-O_R	0	3.21476900	-0.76990100	-2.22361300	H
O-O_R	0	2.38572100	2.02730900	-2.42124600	H
O-O_R	0	-0.20199600	1.17708400	-3.44710900	H
O-O_R	0	-2.74278000	-1.50402700	-3.01132500	L
O-O_R	0	4.63244000	-2.22376600	2.48172800	L
O-O_R	0	2.29940100	4.96777300	1.81278200	L
O-O_R	0	-0.55197100	4.09503000	-2.02527100	L
O-O_R	0	-1.80862400	-4.85897400	-0.04220700	L
O-O_R	0	2.66652700	-3.85283400	-1.31828400	L
O-O_R	0	4.83366400	1.81138700	-0.34101000	L
O-O_R	0	-4.74543000	2.39222100	-1.13190200	L
Rh-	0	0.67792800	0.12443800	-0.60114400	H
Rh-	0	1.52010700	0.21230200	-2.87384800	H
N-N_R	0	-0.06593000	0.16663100	1.18648200	H
S-S_R	0	-0.31152900	-1.17796900	2.11474900	H
O-O_R	0	0.38140100	-2.42891500	1.76768600	H
O-O_R	0	-1.93325900	-1.39082800	1.40194900	H
O-O_R	0	-0.48028600	-0.78240600	3.52890400	H
C-C_R	0	-3.02956100	-0.87496700	2.03641900	H
C-C_R	0	-3.79932100	0.08260000	1.34810400	H
C-C_R	0	-3.45024500	-1.35337900	3.29686200	H

C-C_R	0	-4.99694500	0.53245900	1.90454900	H
H-H_	0	-3.42898400	0.45639600	0.39177900	H
C-C_R	0	-4.65638000	-0.89579700	3.82652300	H
H-H_	0	-2.84883400	-2.09779100	3.81999200	H
C-C_R	0	-5.46422100	0.04987900	3.14993900	H
H-H_	0	-5.58097300	1.27004400	1.35292500	H
H-H_	0	-4.98156200	-1.29552700	4.79054100	H
C-C_3	0	-6.79479800	0.50811900	3.77288700	H
C-C_3	0	-7.72989400	-0.72065200	3.93763300	H
H-H_	0	-7.27479200	-1.50597700	4.56174000	H
H-H_	0	-8.67524100	-0.41509400	4.41757500	H
H-H_	0	-7.97298500	-1.16506300	2.95871100	H
C-C_3	0	-7.52611100	1.55410800	2.90398200	H
H-H_	0	-8.47630500	1.83506000	3.38698100	H
H-H_	0	-6.93318400	2.47530600	2.78391400	H
H-H_	0	-7.76890700	1.16224900	1.90217300	H
C-C_3	0	-6.51938400	1.13902600	5.16440600	H
H-H_	0	-7.46592900	1.47176900	5.62344400	H
H-H_	0	-6.04570200	0.42442300	5.85602800	H
H-H_	0	-5.85435800	2.01372800	5.07664500	H

^{3}2

C-C_R	0	5.65362000	1.52203900	2.51009400	L
C-C_R	0	-4.79723600	-3.69906800	0.55910800	L
C-C_R	0	-3.93669100	4.69342700	1.11775000	L
C-C_R	0	1.61875200	-5.56228600	1.00997500	L
C-C_3	0	3.59367200	4.61706500	-2.12186600	L
C-C_3	0	-2.67955200	2.43864700	-4.39803400	L
C-C_3	0	-0.65420100	-4.21838400	-3.83539200	L
C-C_3	0	5.69439700	-2.12914800	-1.58695800	L
C-C_3	0	-2.61663000	1.18137100	-5.29866200	L
C-C_3	0	4.08493400	5.98199400	-1.58296300	L
C-C_3	0	6.88055400	-2.79208800	-0.84633800	L
C-C_3	0	-1.34086400	-3.38428300	-4.94197500	L
C-C_3	0	4.82175200	3.85380400	-2.66969000	L
C-C_3	0	5.22895400	-3.09758600	-2.69893200	L
C-C_3	0	-1.64827800	3.47856900	-4.89425000	L
C-C_3	0	-1.35676500	-5.59611600	-3.77421000	L
C-C_3	0	-4.09094400	3.05144400	-4.56611400	L
C-C_3	0	6.22739000	-0.82882100	-2.23471000	L
C-C_3	0	0.82044100	-4.47465100	-4.22957700	L
C-C_3	0	2.61359100	4.90749600	-3.28386000	L
C-C_R	0	-0.02858800	-2.20001500	-2.22461200	H
C-C_R	0	3.33028300	-1.04274700	-1.03363100	H

C-C_R	0	2.18868800	2.51684600	-1.29930100	H
C-C_R	0	-1.16991000	1.33514400	-2.51135800	H
C-C_3	0	-0.74070000	-3.54282300	-2.41492200	L
C-C_3	0	4.56440900	-1.80346400	-0.53903400	L
C-C_3	0	2.86135600	3.84978400	-0.95799200	L
C-C_3	0	-2.47182800	2.05466100	-2.88546700	L
C-C_R	0	-3.07619800	-2.38815200	-2.26263400	L
C-C_R	0	4.20243000	-3.18463700	1.60878800	L
C-C_R	0	3.48622700	4.18085000	1.52254500	L
C-C_R	0	-1.64746900	4.13430200	-1.55337700	L
C-C_R	0	-4.17093900	-2.42943700	-1.29770900	L
C-C_R	0	3.22536300	-4.18900800	2.02098100	L
C-C_R	0	4.20939800	3.37919200	2.50638300	L
C-C_R	0	-2.07419200	4.75282900	-0.30329700	L
C-C_R	0	-3.95511100	-3.44162300	-0.45758400	L
C-C_R	0	2.60742800	-4.65078200	0.93425300	L
C-C_R	0	4.90842900	2.44270400	1.86173800	L
C-C_R	0	-3.29416700	4.30807900	-0.00497300	L
C-C_R	0	-2.70870900	-4.10452300	-0.83187800	L
C-C_R	0	3.14817100	-3.96529300	-0.23510300	L
C-C_R	0	4.66875700	2.59348400	0.42951700	L
C-C_R	0	-3.71063700	3.37453000	-1.04824800	L
C-C_R	0	-5.23958100	-1.61399500	-1.17062600	L
C-C_R	0	2.89429800	-4.60528300	3.25826400	L
C-C_R	0	4.20289800	3.46315100	3.84917200	L
C-C_R	0	-1.42009100	5.60424100	0.50685600	L
C-C_R	0	-6.12521900	-1.84053600	-0.16598100	L
C-C_R	0	1.86851500	-5.56641300	3.39018800	L
C-C_R	0	4.97148200	2.52987100	4.57133000	L
C-C_R	0	-2.07443000	6.02311100	1.68621600	L
C-C_R	0	-5.92925700	-2.86727100	0.70329600	L
C-C_R	0	1.24148500	-6.03717100	2.28443100	L
C-C_R	0	5.70221600	1.54916400	3.86310300	L
C-C_R	0	-3.32124300	5.55808600	1.96542400	L
F-F_	0	-5.42691200	-0.58065400	-2.02990500	L
F-F_	0	3.52263900	-4.11096400	4.35413100	L
F-F_	0	3.47758300	4.41228700	4.49188300	L
F-F_	0	-0.17341600	6.04631700	0.20624500	L
F-F_	0	-7.20464700	-1.03131800	-0.02316400	L
F-F_	0	1.50818200	-6.01025300	4.62014500	L
F-F_	0	5.00414700	2.56754900	5.92641600	L
F-F_	0	-1.45882500	6.88252500	2.53599900	L
F-F_	0	-6.80936200	-3.08873300	1.71081400	L
F-F_	0	0.24953100	-6.95489500	2.40044100	L

F-F_	0	6.44418800	0.63585100	4.53770000	L
F-F_	0	-3.95255300	5.95672300	3.09821800	L
F-F_	0	-4.56773000	-4.71937300	1.42275400	L
F-F_	0	0.99558000	-6.01375600	-0.10699000	L
F-F_	0	6.34691200	0.58192000	1.81981500	L
F-F_	0	-5.17988900	4.22918700	1.39609700	L
H-H_	0	7.72180300	-2.99334600	-1.54482500	L
H-H_	0	-1.21110600	-3.87522500	-5.93074200	L
H-H_	0	-2.87979200	1.43798100	-6.34776300	L
H-H_	0	4.56743500	6.57647300	-2.38910400	L
H-H_	0	6.57951100	-3.76143600	-0.39441300	L
H-H_	0	4.83196800	5.84704000	-0.77165400	L
H-H_	0	-0.91881700	-2.36403400	-5.02245200	L
H-H_	0	-3.33561600	0.41125000	-4.94659900	L
H-H_	0	7.25634200	-2.13250500	-0.03417700	L
H-H_	0	3.23598300	6.57459800	-1.17810500	L
H-H_	0	-2.43214600	-3.30208100	-4.76107500	L
H-H_	0	-1.60508100	0.73148300	-5.32289000	L
H-H_	0	6.04196100	-3.25112200	-3.44123000	L
H-H_	0	5.27557100	4.41195700	-3.51708200	L
H-H_	0	-1.82140900	3.71129500	-5.96727000	L
H-H_	0	-1.26877900	-6.12999700	-4.74544100	L
H-H_	0	4.34865500	-2.71328400	-3.24927500	L
H-H_	0	4.55721800	2.84584800	-3.04330000	L
H-H_	0	-0.60685600	3.11476900	-4.80009800	L
H-H_	0	-2.43929500	-5.48522800	-3.55016100	L
H-H_	0	4.98001000	-4.09469500	-2.28145100	L
H-H_	0	5.60612900	3.74972400	-1.89208000	L
H-H_	0	-1.74081100	4.43039600	-4.33138200	L
H-H_	0	-0.90241300	-6.23515200	-2.98614000	L
H-H_	0	3.09908400	5.54205200	-4.05676400	L
H-H_	0	7.09004600	-1.04779600	-2.90077300	L
H-H_	0	-4.29705100	3.28719700	-5.63287200	L
H-H_	0	0.87785800	-5.04743900	-5.18056500	L
H-H_	0	1.71721700	5.44954100	-2.91415300	L
H-H_	0	6.57425200	-0.11712900	-1.45567100	L
H-H_	0	1.33791500	-5.06760300	-3.44570800	L
H-H_	0	-4.19001100	3.99619700	-3.98997600	L
H-H_	0	2.27896000	3.98045200	-3.78955000	L
H-H_	0	-4.87192700	2.34412700	-4.21171700	L
H-H_	0	1.38251000	-3.53339000	-4.38611000	L
H-H_	0	5.45916100	-0.32529900	-2.85381300	L
H-H_	0	-0.21521000	-4.23673200	-1.72261200	L
H-H_	0	5.03612700	-1.11447000	0.19594000	L

H-H_	0	2.02154000	4.51253700	-0.65140100	L
H-H_	0	-3.27304900	1.31971300	-2.64760600	L
N-N_R	0	-2.13705500	-3.42882000	-1.95835600	L
N-N_R	0	4.13480100	-3.01188900	0.18752000	L
N-N_R	0	3.74333300	3.66857500	0.20912200	L
N-N_R	0	-2.66047500	3.22667200	-2.01174300	L
O-O_R	0	-0.03910100	-1.76128900	-1.02035500	H
O-O_R	0	2.53183000	-0.66818100	-0.10051900	H
O-O_R	0	1.46082000	2.02985200	-0.35965500	H
O-O_R	0	-1.10450100	0.93984700	-1.28980200	H
O-O_R	0	0.50577000	-1.60881100	-3.22131600	H
O-O_R	0	3.15699200	-0.84434000	-2.28114300	H
O-O_R	0	2.38687000	1.96950500	-2.43339500	H
O-O_R	0	-0.25335300	1.18803500	-3.38301200	H
O-O_R	0	-2.79402900	-1.38191800	-2.96438700	L
O-O_R	0	4.69850500	-2.32601700	2.38665300	L
O-O_R	0	2.49704600	4.91105400	1.79928200	L
O-O_R	0	-0.46077700	4.12808300	-1.97352800	L
O-O_R	0	-2.04483500	-4.84664000	-0.05957000	L
O-O_R	0	2.54691100	-3.89347500	-1.33887500	L
O-O_R	0	4.88904800	1.68608700	-0.41490400	L
O-O_R	0	-4.68381800	2.58039900	-0.95021100	L
Rh-	0	0.68031400	0.12958700	-0.58160000	H
Rh-	0	1.46401300	0.17719700	-2.86445700	H
N-N_3	0	0.08553000	-0.05469600	1.26764000	H
S-S_3+6	0	-0.43978300	-1.32554900	2.16761500	H
O-O_2	0	0.29380900	-2.56877400	1.87656100	H
O-O_R	0	-1.98573800	-1.58373100	1.45175200	H
O-O_2	0	-0.63925900	-0.86399500	3.55431000	H
C-C_R	0	-3.09473100	-0.99391300	2.05038200	H
C-C_R	0	-3.70353300	0.10080000	1.42141800	H
C-C_R	0	-3.65651500	-1.55739400	3.20880200	H
C-C_R	0	-4.89240300	0.61859600	1.95057700	H
H-H_	0	-3.24136500	0.52590400	0.52802200	H
C-C_R	0	-4.84614000	-1.02914600	3.71344200	H
H-H_	0	-3.17056000	-2.41138700	3.68380400	H
C-C_R	0	-5.49759100	0.06595400	3.09949100	H
H-H_	0	-5.35655100	1.46764600	1.44764600	H
H-H_	0	-5.27868500	-1.48884300	4.60609500	H
C-C_3	0	-6.81445200	0.60435100	3.69177600	H
C-C_3	0	-7.88105800	-0.52293400	3.68627100	H
H-H_	0	-7.55021800	-1.40780200	4.25293000	H
H-H_	0	-8.81794400	-0.16214400	4.14427200	H
H-H_	0	-8.10821300	-0.84691200	2.65756200	H

C-C_3	0	-7.37607000	1.79942900	2.89202700	H
H-H_	0	-8.32559100	2.13242400	3.34250400	H
H-H_	0	-6.68701300	2.65938200	2.90362000	H
H-H_	0	-7.58389500	1.53288600	1.84222100	H
C-C_3	0	-6.56550200	1.07165900	5.15075500	H
H-H_	0	-7.49957800	1.46071400	5.59115500	H
H-H_	0	-6.21066600	0.24905000	5.79193300	H
H-H_	0	-5.80995300	1.87368600	5.18470900	H

[1]INT1

C-C_R	0	5.84008900	-1.35992700	0.42745800	L
C-C_R	0	-5.82528100	3.56256800	-1.72768000	L
C-C_R	0	-2.88299700	-4.45519900	-3.40543300	L
C-C_R	0	1.11272000	5.78236200	0.73599300	L
C-C_3	0	1.98509600	-4.95104900	3.07632600	L
C-C_3	0	-4.75547400	-3.48794800	2.34515900	L
C-C_3	0	-3.34806400	3.39739300	3.70101200	L
C-C_3	0	3.36619900	1.81009800	4.46560900	L
C-C_3	0	-5.28565900	-2.43880600	3.35212300	L
C-C_3	0	2.79824900	-6.19988300	2.65883800	L
C-C_3	0	4.70353900	2.58794600	4.50963900	L
C-C_3	0	-4.34210000	2.39828600	4.33826000	L
C-C_3	0	2.75243500	-4.24614900	4.21925500	L
C-C_3	0	2.32809700	2.60625400	5.29094700	L
C-C_3	0	-3.93162000	-4.55368200	3.10399900	L
C-C_3	0	-4.07941200	4.75232400	3.54159200	L
C-C_3	0	-5.98717200	-4.19067200	1.72516600	L
C-C_3	0	3.61420100	0.43869700	5.13885300	L
C-C_3	0	-2.16213800	3.63186000	4.66703900	L
C-C_3	0	0.62046800	-5.45005700	3.60979300	L
C-C_R	0	-1.96378100	1.66015900	2.24966000	H
C-C_R	0	1.67394500	0.79682200	2.68772700	H
C-C_R	0	0.91176100	-2.78523000	1.97514500	H
C-C_R	0	-2.71695400	-1.95682100	1.59253600	H
C-C_3	0	-2.83714000	2.92206900	2.28784600	L
C-C_3	0	2.93564400	1.62363000	2.96152100	L
C-C_3	0	1.78406600	-4.03325900	1.81189600	L
C-C_3	0	-3.93879100	-2.79109600	1.19351100	L
C-C_R	0	-4.62873900	1.57317800	0.96023100	L
C-C_R	0	3.69272300	3.37820000	1.22589300	L
C-C_R	0	3.53702100	-4.03873500	-0.09097400	L
C-C_R	0	-2.36882800	-4.56604000	0.11948000	L
C-C_R	0	-5.41541300	1.80516200	-0.24634600	L
C-C_R	0	3.09333300	4.54008900	0.57499300	L

C-C_R	0	4.64554800	-3.17162900	-0.48208900	L
C-C_R	0	-2.03533100	-4.88799700	-1.26371400	L
C-C_R	0	-5.22730800	3.06792000	-0.62945400	L
C-C_R	0	1.91013900	4.77713300	1.14317700	L
C-C_R	0	4.88319100	-2.30890900	0.50925400	L
C-C_R	0	-2.96047000	-4.35628300	-2.06105800	L
C-C_R	0	-4.32550900	3.71389700	0.32136900	L
C-C_R	0	1.70374000	3.79991800	2.20775300	L
C-C_R	0	3.93829900	-2.57706300	1.59028000	L
C-C_R	0	-3.94327000	-3.66685300	-1.22973900	L
C-C_R	0	-6.20778400	0.96041600	-0.93933000	L
C-C_R	0	3.56021300	5.29431700	-0.43747100	L
C-C_R	0	5.34774400	-3.14745100	-1.62906100	L
C-C_R	0	-0.97715400	-5.55207900	-1.76255100	L
C-C_R	0	-6.83399300	1.41307600	-2.05619100	L
C-C_R	0	2.76195300	6.36332600	-0.89958400	L
C-C_R	0	6.36624300	-2.18401300	-1.75776800	L
C-C_R	0	-0.87935400	-5.67462300	-3.16584600	L
C-C_R	0	-6.66387400	2.69823100	-2.46580900	L
C-C_R	0	1.55907300	6.60394300	-0.32197200	L
C-C_R	0	6.59682700	-1.28334400	-0.69329400	L
C-C_R	0	-1.83627900	-5.12189700	-3.95769400	L
F-F_	0	-6.37441900	-0.32285800	-0.53211400	L
F-F_	0	4.76298700	5.03362200	-1.00681600	L
F-F_	0	5.08578600	-4.02044800	-2.63350800	L
F-F_	0	-0.02982700	-6.08150700	-0.94894500	L
F-F_	0	-7.63014900	0.57733200	-2.76897100	L
F-F_	0	3.19670800	7.14274900	-1.92085300	L
F-F_	0	7.10840800	-2.11748300	-2.89021100	L
F-F_	0	0.16931300	-6.33362000	-3.71869800	L
F-F_	0	-7.28896000	3.14820900	-3.58218700	L
F-F_	0	0.78270900	7.62534100	-0.76187100	L
F-F_	0	7.56413900	-0.33867900	-0.79991600	L
F-F_	0	-1.74213900	-5.23147200	-5.30666800	L
F-F_	0	-5.63188000	4.84730400	-2.11699100	L
F-F_	0	-0.08934400	6.00408500	1.32463700	L
F-F_	0	6.04174900	-0.49118600	1.45022800	L
F-F_	0	-3.83292800	-3.89564900	-4.19624000	L
H-H_	0	5.06952200	2.68962300	5.55451300	L
H-H_	0	-4.69115800	2.77694900	5.32332200	L
H-H_	0	-5.95963300	-2.91590800	4.09636900	L
H-H_	0	2.91706300	-6.90022000	3.51410100	L
H-H_	0	4.58619600	3.61240100	4.09615500	L
H-H_	0	3.81557400	-5.91851800	2.31201900	L

H-H_	0	-3.88740600	1.40447700	4.51315400	L
H-H_	0	-5.86470200	-1.64935500	2.82726100	L
H-H_	0	5.48505200	2.06108700	3.92022400	L
H-H_	0	2.28849600	-6.74460700	1.83468000	L
H-H_	0	-5.23867200	2.26754500	3.69718900	L
H-H_	0	-4.46901900	-1.95468400	3.92227700	L
H-H_	0	2.66051100	2.69175900	6.34816200	L
H-H_	0	2.82431800	-4.91484600	5.10425100	L
H-H_	0	-4.53066800	-4.98378000	3.93566500	L
H-H_	0	-4.39182400	5.15364100	4.53025000	L
H-H_	0	1.33334100	2.12076900	5.29766000	L
H-H_	0	2.25312700	-3.31515200	4.54975000	L
H-H_	0	-3.00745900	-4.13265700	3.54531400	L
H-H_	0	-4.99583600	4.64438800	2.92349000	L
H-H_	0	2.21353000	3.63790800	4.89947100	L
H-H_	0	3.79000700	-4.00157100	3.91267400	L
H-H_	0	-3.65337400	-5.39335400	2.43440300	L
H-H_	0	-3.41742700	5.50365300	3.05898900	L
H-H_	0	0.76571500	-6.18343900	4.43247000	L
H-H_	0	4.02090000	0.57393700	6.16454900	L
H-H_	0	-6.61886300	-4.65376500	2.51419100	L
H-H_	0	-2.52100800	4.04853200	5.63311600	L
H-H_	0	0.04538700	-5.95420500	2.80422100	L
H-H_	0	4.35152900	-0.15469100	4.55746900	L
H-H_	0	-1.44280300	4.35742600	4.23177800	L
H-H_	0	-5.67947400	-4.99743300	1.02595700	L
H-H_	0	0.00350400	-4.62551700	4.01755100	L
H-H_	0	-6.61335400	-3.46405100	1.16321500	L
H-H_	0	-1.61849900	2.69620900	4.90224700	L
H-H_	0	2.68516400	-0.15571300	5.23754900	L
H-H_	0	-2.17508600	3.73441400	1.91115400	L
H-H_	0	3.75887100	1.05111100	2.47714400	L
H-H_	0	1.23726000	-4.66150100	1.07408700	L
H-H_	0	-4.63715000	-2.05537000	0.73690100	L
N-N_R	0	-3.96058600	2.78139900	1.34527600	L
N-N_R	0	2.83352100	2.92063200	2.27319300	L
N-N_R	0	3.07392100	-3.65563900	1.20857800	L
N-N_R	0	-3.55449600	-3.75634200	0.14718500	L
O-O_R	0	-1.38515500	1.43149800	1.12584200	H
O-O_R	0	1.42922800	0.57526100	1.44572100	H
O-O_R	0	0.65864300	-2.17267500	0.87712100	H
O-O_R	0	-2.15064400	-1.34353800	0.61763400	H
O-O_R	0	-1.88085900	0.92205300	3.28599100	H
O-O_R	0	0.95291900	0.39629800	3.65723200	H

O-O_R	0	0.51090300	-2.43011600	3.13240900	H
O-O_R	0	-2.33073300	-1.92111200	2.80670300	H
O-O_R	0	-4.72208200	0.55786800	1.69846600	L
O-O_R	0	4.90186600	3.04017000	1.12255700	L
O-O_R	0	2.84573500	-4.71089300	-0.90223600	L
O-O_R	0	-1.54208400	-4.61686100	1.06744300	L
O-O_R	0	-3.70959100	4.79194500	0.10645200	L
O-O_R	0	0.58830200	3.54421600	2.73167400	L
O-O_R	0	3.65597500	-1.75107600	2.49739800	L
O-O_R	0	-4.76853500	-2.81969600	-1.66410100	L
Rh-	0	-0.34255300	-0.36859500	0.89905200	H
Rh-	0	-0.69666400	-0.76104200	3.26182800	H
N-N_R	0	-0.15914500	-0.10096200	-1.02594900	H
S-S_R	0	0.57977700	-1.08088000	-2.10634100	H
O-O_R	0	0.63232300	-0.41820500	-3.42603800	H
O-O_R	0	2.22498500	-1.02664900	-1.45155100	H
O-O_R	0	0.19185400	-2.50032200	-2.00711900	H
C-C_R	0	3.17987600	-0.29940000	-2.11298900	H
C-C_R	0	3.83519200	0.72142800	-1.39749600	H
C-C_R	0	3.57966800	-0.60645400	-3.43265700	H
C-C_R	0	4.88584500	1.42013700	-1.99456000	H
H-H_	0	3.49458300	0.94305300	-0.38502600	H
C-C_R	0	4.63524800	0.10112300	-4.00524700	H
H-H_	0	3.07759200	-1.40569400	-3.97879100	H
C-C_R	0	5.31671000	1.13022500	-3.30997500	H
H-H_	0	5.37834700	2.20823900	-1.42395700	H
H-H_	0	4.94082700	-0.16045400	-5.02162900	H
C-C_3	0	6.46935000	1.88732000	-3.99273100	H
C-C_3	0	5.92968000	2.58711500	-5.26967500	H
H-H_	0	5.51378900	1.86533600	-5.99047800	H
H-H_	0	6.74370100	3.13483800	-5.77450300	H
H-H_	0	5.13456300	3.30817600	-5.01891200	H
C-C_3	0	7.09328500	2.96281200	-3.07722100	H
H-H_	0	7.92020800	3.46381900	-3.60662900	H
H-H_	0	7.50856300	2.52719000	-2.15303300	H
H-H_	0	6.36292400	3.73982100	-2.79833900	H
C-C_3	0	7.58585100	0.88387600	-4.38845800	H
H-H_	0	8.39813400	1.41194600	-4.91627900	H
H-H_	0	7.21320800	0.09089200	-5.05596400	H
H-H_	0	8.01742700	0.40070300	-3.49693600	H
C-C_R	0	-3.15760700	2.14525500	-3.43470100	H
C-C_R	0	-3.92485300	2.63573000	-4.51089900	H
C-C_R	0	-4.71910500	1.77728200	-5.28499400	H
C-C_R	0	-4.76205200	0.40505900	-4.99439500	H

C-C_R	0	-4.00546200	-0.09787700	-3.92495100	H
C-C_R	0	-3.21705800	0.76417500	-3.15134800	H
H-H_	0	-3.90942900	3.70766900	-4.73365600	H
H-H_	0	-5.31162000	2.18239300	-6.11103000	H
H-H_	0	-5.38546700	-0.26603800	-5.59247900	H
H-H_	0	-4.03166300	-1.16509700	-3.68996800	H
H-H_	0	-2.64023700	0.36791600	-2.31014700	H
C-C_3	0	-2.29140600	3.06197800	-2.59469500	H
H-H_	0	-2.72828400	4.07881600	-2.58080000	H
H-H_	0	-2.29346500	2.69593200	-1.55313300	H
C-C_3	0	-0.80722800	3.16824400	-3.04611300	H
H-H_	0	-0.40694400	2.14056400	-3.09832200	H
C-C_3	0	-0.00245500	3.94196400	-1.98800000	H
H-H_	0	-0.38610300	4.97276000	-1.87851800	H
H-H_	0	1.06288800	4.01031300	-2.26726300	H
H-H_	0	-0.06354000	3.45051600	-1.00279500	H
C-C_3	0	-0.66024900	3.81349500	-4.43349400	H
H-H_	0	-1.03917800	4.85225100	-4.43062200	H
H-H_	0	-1.21496300	3.25268600	-5.20265000	H
H-H_	0	0.39915900	3.84664800	-4.73995300	H

[1]TS1

C-C_R	0	-5.93656300	-1.12559500	-0.58944000	L
C-C_R	0	5.62824700	3.27286000	2.38811500	L
C-C_R	0	2.97638900	-5.22542800	2.67314200	L
C-C_R	0	-0.89074200	5.94065400	0.12878400	L
C-C_3	0	-2.04901200	-4.22339600	-3.76018400	L
C-C_3	0	4.68787100	-2.97252800	-2.69381900	L
C-C_3	0	3.43104600	3.99752300	-3.17298400	L
C-C_3	0	-3.35014800	2.65651600	-4.15668800	L
C-C_3	0	5.23881600	-1.76972600	-3.49661900	L
C-C_3	0	-2.87069800	-5.51778300	-3.54789200	L
C-C_3	0	-4.67725100	3.45091300	-4.09622400	L
C-C_3	0	4.40070000	3.06300800	-3.93432500	L
C-C_3	0	-2.80775700	-3.34260400	-4.78045800	L
C-C_3	0	-2.30691800	3.53160900	-4.89016900	L
C-C_3	0	3.87009700	-3.88640300	-3.63632600	L
C-C_3	0	4.19897400	5.29716600	-2.83025700	L
C-C_3	0	5.90738300	-3.78159300	-2.18998100	L
C-C_3	0	-3.62103400	1.37787900	-4.98573500	L
C-C_3	0	2.26034100	4.39244600	-4.10456000	L
C-C_3	0	-0.68454000	-4.64170000	-4.35984700	L
C-C_R	0	2.00699500	2.11028900	-1.96449900	H
C-C_R	0	-1.66343800	1.41661400	-2.51783300	H

C-C_R	0	-0.97737400	-2.25618200	-2.33426000	H
C-C_R	0	2.64736400	-1.57568800	-1.71894100	H
C-C_3	0	2.89389400	3.35715200	-1.83715600	L
C-C_3	0	-2.91341000	2.28892900	-2.68853200	L
C-C_3	0	-1.85128700	-3.51461300	-2.36700700	L
C-C_3	0	3.85640300	-2.48054700	-1.44945700	L
C-C_R	0	4.76889900	1.88891300	-0.76920500	L
C-C_R	0	-3.61217600	3.81267500	-0.72518300	L
C-C_R	0	-3.65144500	-3.86881200	-0.54101600	L
C-C_R	0	2.25266100	-4.40090500	-0.71722100	L
C-C_R	0	5.44550500	1.88017900	0.52314500	L
C-C_R	0	-2.92666200	4.78104200	0.12457500	L
C-C_R	0	-4.77457600	-3.09185000	-0.02295000	L
C-C_R	0	1.99038200	-5.07075600	0.55196600	L
C-C_R	0	5.16424400	3.02239400	1.15069800	L
C-C_R	0	-1.76176100	5.08964900	-0.44597500	L
C-C_R	0	-4.97934600	-2.04689900	-0.82953700	L
C-C_R	0	2.95984800	-4.75137100	1.40938900	L
C-C_R	0	4.28143000	3.81944000	0.30051400	L
C-C_R	0	-1.63489200	4.32953200	-1.68656400	L
C-C_R	0	-3.99913200	-2.10074800	-1.91108600	L
C-C_R	0	3.87608900	-3.83026600	0.74201600	L
C-C_R	0	6.20106700	0.91928700	1.09594400	L
C-C_R	0	-3.29780000	5.29833700	1.31073000	L
C-C_R	0	-5.51424900	-3.29253200	1.08221400	L
C-C_R	0	0.97322800	-5.87414300	0.91027700	L
C-C_R	0	6.69866600	1.12886500	2.34198300	L
C-C_R	0	-2.42169400	6.20180200	1.95069300	L
C-C_R	0	-6.53466400	-2.36621400	1.36896500	L
C-C_R	0	0.96918600	-6.38093100	2.22837400	L
C-C_R	0	6.43256600	2.28723200	3.00177400	L
C-C_R	0	-1.23896400	6.51910400	1.36863000	L
C-C_R	0	-6.72842400	-1.26817800	0.50018700	L
C-C_R	0	1.97390500	-6.04630400	3.08140800	L
F-F_	0	6.45450800	-0.24280400	0.44294600	L
F-F_	0	-4.47960400	4.95861300	1.88303800	L
F-F_	0	-5.28573400	-4.35029900	1.89988100	L
F-F_	0	-0.01737700	-6.18843100	0.03841400	L
F-F_	0	7.45625600	0.17276600	2.93556400	L
F-F_	0	-2.76070500	6.74363000	3.14694600	L
F-F_	0	-7.31462200	-2.52375700	2.46639300	L
F-F_	0	-0.03418200	-7.19563800	2.63964100	L
F-F_	0	6.92115300	2.49014000	4.25060300	L
F-F_	0	-0.38612800	7.37853400	1.97980000	L

F-F_	0	-7.69669600	-0.35583000	0.76341600	L
F-F_	0	1.97158100	-6.52928600	4.34921800	L
F-F_	0	5.32386500	4.42568900	3.03450700	L
F-F_	0	0.29815600	6.22744200	-0.45848900	L
F-F_	0	-6.10280900	-0.06782500	-1.42308000	L
F-F_	0	3.97127300	-4.88048200	3.52869300	L
H-H_	0	-5.04906200	3.68128300	-5.11835000	L
H-H_	0	4.75859800	3.55482000	-4.86469100	L
H-H_	0	5.90625300	-2.11792700	-4.31456100	L
H-H_	0	-2.98299300	-6.07770300	-4.50179500	L
H-H_	0	-4.54346500	4.41674200	-3.56367300	L
H-H_	0	-3.89108900	-5.28875000	-3.17292500	L
H-H_	0	3.92200900	2.11048900	-4.23186800	L
H-H_	0	5.83159300	-1.09931700	-2.83868900	L
H-H_	0	-5.46158800	2.86751800	-3.56677900	L
H-H_	0	-2.37125900	-6.18520300	-2.81242500	L
H-H_	0	5.29493500	2.83059700	-3.31996800	L
H-H_	0	4.43302100	-1.17535400	-3.96958500	L
H-H_	0	-2.64451000	3.74524800	-5.92742800	L
H-H_	0	-2.89106500	-3.86866400	-5.75608400	L
H-H_	0	4.48651700	-4.18830800	-4.51062300	L
H-H_	0	4.53752800	5.81389200	-3.75462500	L
H-H_	0	-1.31874800	3.03775400	-4.96119700	L
H-H_	0	-2.29536200	-2.37965000	-4.96868500	L
H-H_	0	2.96565400	-3.38380600	-4.02977000	L
H-H_	0	5.10162200	5.08154200	-2.21969700	L
H-H_	0	-2.17680700	4.50845500	-4.38063900	L
H-H_	0	-3.84040700	-3.13067800	-4.43518600	L
H-H_	0	3.55926900	-4.81694400	-3.11833000	L
H-H_	0	3.55179300	5.99874200	-2.26043700	L
H-H_	0	-0.83033800	-5.24009300	-5.28526900	L
H-H_	0	-4.03068600	1.63927000	-5.98553200	L
H-H_	0	6.55052600	-4.10038200	-3.03898000	L
H-H_	0	2.63914600	4.92063100	-5.00635800	L
H-H_	0	-0.11452500	-5.26569300	-3.63906400	L
H-H_	0	-4.36425900	0.73012400	-4.47418500	L
H-H_	0	1.55962700	5.07694400	-3.58127500	L
H-H_	0	5.58621600	-4.69862300	-1.65122800	L
H-H_	0	-0.06185900	-3.76875500	-4.63655700	L
H-H_	0	6.52695500	-3.17032600	-1.49840900	L
H-H_	0	1.69088300	3.51231200	-4.46157100	L
H-H_	0	-2.70115200	0.78656300	-5.15998300	L
H-H_	0	2.23313800	4.12522100	-1.37568300	L
H-H_	0	-3.74186500	1.67153800	-2.27397800	L

H-H_	0	-1.30922600	-4.24997100	-1.73174400	L
H-H_	0	4.55080700	-1.84900100	-0.85056900	L
N-N_R	0	4.00127400	3.09249800	-0.90160200	L
N-N_R	0	-2.79093100	3.49635000	-1.85351200	L
N-N_R	0	-3.14501800	-3.23600300	-1.72071600	L
N-N_R	0	3.44597400	-3.61396300	-0.60482600	L
O-O_R	0	1.44591700	1.72039000	-0.87287000	H
O-O_R	0	-1.42571300	1.05212400	-1.30971900	H
O-O_R	0	-0.73886700	-1.80391300	-1.16039800	H
O-O_R	0	2.08602200	-1.09694600	-0.66362000	H
O-O_R	0	1.88841400	1.53395800	-3.09056000	H
O-O_R	0	-0.95496900	1.12599100	-3.53586900	H
O-O_R	0	-0.55624200	-1.74384700	-3.42417800	H
O-O_R	0	2.27137400	-1.36080900	-2.91527800	H
O-O_R	0	4.62006300	0.87860200	-1.50459000	L
O-O_R	0	-4.61255000	3.13157900	-0.37625000	L
O-O_R	0	-2.98999300	-4.69812300	0.13914800	L
O-O_R	0	1.40280800	-4.25550700	-1.63404300	L
O-O_R	0	3.57217000	4.77552000	0.71372900	L
O-O_R	0	-0.54051700	4.12197000	-2.27308500	L
O-O_R	0	-3.69694900	-1.11912500	-2.63901000	L
O-O_R	0	5.02650300	-3.53086700	1.15893100	L
Rh-	0	0.32042100	-0.04140500	-0.86607200	H
Rh-	0	0.66731600	-0.11027400	-3.27157200	H
N-N_3	0	-0.33662600	0.02038900	1.02761000	H
S-S_3+6	0	-0.46125200	-1.42235300	1.91785400	H
O-O_2	0	-0.40589600	-1.12344600	3.36279100	H
O-O_R	0	-2.10823000	-1.70582500	1.48942100	H
O-O_2	0	0.23746300	-2.59702100	1.37843000	H
C-C_R	0	-3.07621900	-0.98076400	2.18400400	H
C-C_R	0	-3.63406000	0.16449100	1.60047300	H
C-C_R	0	-3.54412800	-1.45053400	3.42217700	H
C-C_R	0	-4.66748300	0.83628300	2.26664500	H
H-H_	0	-3.24483600	0.51830200	0.64387400	H
C-C_R	0	-4.58381100	-0.76977300	4.06270400	H
H-H_	0	-3.10033400	-2.34682200	3.85906100	H
C-C_R	0	-5.16855800	0.39117600	3.50787600	H
H-H_	0	-5.09021500	1.72743300	1.80167600	H
H-H_	0	-4.94095400	-1.15529100	5.02172600	H
C-C_3	0	-6.28951500	1.13125000	4.26520400	H
C-C_3	0	-5.74170300	1.61273600	5.63532600	H
H-H_	0	-5.39927100	0.77113100	6.25852600	H
H-H_	0	-6.52755000	2.14807500	6.19569900	H
H-H_	0	-4.88924100	2.29885600	5.50030900	H

C-C_3	0	-6.80661600	2.36401400	3.49190100	H
H-H_	0	-7.61194900	2.85091600	4.06638500	H
H-H_	0	-7.22176800	2.08799200	2.50811800	H
H-H_	0	-6.01368200	3.11392200	3.33505300	H
C-C_3	0	-7.48747500	0.17435200	4.49965100	H
H-H_	0	-8.27045000	0.68156100	5.08922900	H
H-H_	0	-7.19057800	-0.73346100	5.04846500	H
H-H_	0	-7.93448100	-0.14048600	3.54299700	H
C-C_R	0	2.99972900	1.04369800	3.32462700	H
C-C_R	0	3.49170300	1.43142200	4.58752500	H
C-C_R	0	4.25319300	0.55190000	5.36923700	H
C-C_R	0	4.53011700	-0.73978200	4.89907800	H
C-C_R	0	4.04508800	-1.13846600	3.64451500	H
C-C_R	0	3.29350600	-0.25372000	2.85900100	H
H-H_	0	3.28719600	2.44296600	4.95415400	H
H-H_	0	4.63366600	0.87801300	6.34166900	H
H-H_	0	5.12381700	-1.43121000	5.50376400	H
H-H_	0	4.25470600	-2.14476200	3.27661600	H
H-H_	0	2.93905900	-0.57258400	1.87425000	H
C-C_3	0	2.19846300	2.01469100	2.48385400	H
H-H_	0	2.48619700	3.04828600	2.76751300	H
H-H_	0	2.45129800	1.90242600	1.41863600	H
C-C_3	0	0.66118600	1.96188100	2.60108100	H
H-H_	0	0.32255600	0.97769000	1.96494800	H
C-C_3	0	-0.02156000	3.08437100	1.82484500	H
H-H_	0	0.10602000	4.03809900	2.37113300	H
H-H_	0	-1.10196800	2.89407200	1.73032700	H
H-H_	0	0.40267300	3.19765800	0.81742300	H
C-C_3	0	0.10590800	1.78773800	4.00406400	H
H-H_	0	0.33958300	2.69444000	4.59722800	H
H-H_	0	0.54120300	0.92240600	4.52061700	H
H-H_	0	-0.98824600	1.67269800	3.98510200	H

^{1}INT2

Rh-	0	-0.47798900	-0.43721600	0.86823200	H
Rh-	0	-1.04075800	-1.06949900	3.14246800	H
O-O_R	0	-1.45252000	1.37656300	1.23187100	H
C-C_R	0	-2.08698200	1.52331700	2.33812100	H
F-F_	0	-6.78844900	0.02387700	-0.34522400	L
N-N_R	0	-3.99130100	2.81715200	1.48000200	L
O-O_R	0	-2.11164500	0.67847400	3.29346500	H
C-C_3	0	-2.89168400	2.82292300	2.45940000	L
F-F_	0	-7.69647600	0.91806900	-2.74573000	L
O-O_R	0	-4.83030300	0.60768200	1.72212200	L

C-C_R	0	-4.87666800	1.73462000	1.16325000	L
F-F_	0	-6.87280300	3.32435100	-3.70431800	L
O-O_R	0	-3.35255300	4.67030500	0.14339700	L
C-C_R	0	-5.10358700	3.17640900	-0.56949200	L
C-C_R	0	-4.16062500	3.73707300	0.39603200	L
C-C_R	0	-6.38149700	1.22632600	-0.82404000	L
C-C_R	0	-6.83444600	1.67610200	-2.02272000	L
C-C_R	0	-6.42404900	2.87933000	-2.50427300	L
C-C_R	0	-5.52051900	3.66233600	-1.75239100	L
C-C_3	0	-3.40856700	3.22289100	3.89336000	L
C-C_3	0	-4.47585700	2.24833500	4.44508000	L
C-C_3	0	-4.05030800	4.62904400	3.81472300	L
C-C_3	0	-2.23217300	3.31054900	4.89425000	L
C-C_R	0	-5.52291700	1.99362400	-0.11923200	L
F-F_	0	-5.08696700	4.86168500	-2.21375800	L
O-O_R	0	0.63259800	-0.07701300	3.80819400	H
C-C_3	0	2.72447300	1.10540100	3.40213600	L
F-F_	0	3.60933400	6.47637500	-1.52759700	L
O-O_R	0	4.83538300	2.28856300	1.62895600	L
C-C_R	0	3.76601800	2.90760900	1.87530400	L
F-F_	0	1.08583800	7.01114300	-0.65842100	L
O-O_R	0	0.51342300	3.14777500	3.02520100	L
C-C_R	0	3.23897100	4.01902800	1.08787700	L
C-C_R	0	1.99939000	4.27481600	1.50510800	L
C-C_R	0	1.67448000	3.34515100	2.58217900	L
C-C_R	0	3.81073900	4.71518700	0.08743000	L
C-C_R	0	3.06424100	5.74721400	-0.52220400	L
C-C_R	0	1.80431600	6.00891600	-0.09475200	L
C-C_R	0	1.24534800	5.24209900	0.94992800	L
C-C_3	0	3.00459400	1.16827300	4.94978500	L
C-C_3	0	4.36860700	1.86219300	5.18230800	L
C-C_3	0	1.92585600	1.97165600	5.71264600	L
C-C_3	0	3.10867800	-0.25487700	5.54957200	L
F-F_	0	-0.01688200	5.48261100	1.38529100	L
O-O_R	0	1.30460300	0.30710200	1.67268900	H
C-C_R	0	1.45065800	0.38150000	2.94539400	H
N-N_R	0	2.77519300	2.45190800	2.80336500	L
F-F_	0	5.07605800	4.44572900	-0.31790400	L
O-O_R	0	0.41956100	-2.28762600	0.71798200	H
C-C_R	0	0.53142600	-3.04406400	1.74662600	H
N-N_R	0	2.71108900	-3.96926400	1.10731800	L
F-F_	0	5.09825600	-3.89246400	-2.53251200	L
O-O_R	0	0.05580800	-2.79831600	2.90391800	H
C-C_3	0	1.33569900	-4.32283700	1.50000700	L

F-F_	0	7.20337500	-2.07585700	-2.31298400	L
O-O_R	0	2.66553100	-4.70075100	-1.14764400	L
F-F_	0	7.50719200	-0.63893500	0.05077700	L
C-C_R	0	3.29370300	-4.18877200	-0.18258500	L
O-O_R	0	3.23894300	-2.30879300	2.72018700	L
C-C_R	0	4.46896300	-3.33414200	-0.33341600	L
C-C_R	0	4.63384100	-2.63265600	0.79069000	L
C-C_R	0	3.57341200	-3.00267400	1.72396400	L
C-C_R	0	5.28686000	-3.18469700	-1.39094600	L
C-C_R	0	6.34708200	-2.26506600	-1.27907400	L
C-C_R	0	6.49867600	-1.53635200	-0.07748000	L
C-C_R	0	5.62757600	-1.73123300	0.94096600	L
C-C_3	0	1.32066400	-5.41968700	2.62965400	L
C-C_3	0	2.07769000	-6.66846200	2.11767500	L
C-C_3	0	2.00569200	-4.95236800	3.93490100	L
C-C_3	0	-0.13039400	-5.85761400	2.94324200	L
F-F_	0	5.75590300	-1.02902700	2.09493100	L
O-O_R	0	-2.29108300	-1.23699800	0.31321900	H
F-F_	0	-0.42942600	-5.81639600	-1.90259700	L
C-C_R	0	-2.97550800	-1.92123700	1.15919700	H
N-N_R	0	-3.82269400	-3.40578000	-0.60404500	L
O-O_R	0	-2.69479400	-2.05141300	2.39432300	H
F-F_	0	-0.10609700	-5.66238700	-4.66838200	L
C-C_3	0	-4.21444500	-2.60375300	0.56923900	L
O-O_R	0	-1.91812700	-4.53512700	0.25040200	L
F-F_	0	-1.83941700	-4.17302100	-6.14046000	L
C-C_R	0	-2.70065400	-4.29587400	-0.70650000	L
O-O_R	0	-4.86198200	-2.10224600	-2.29336600	L
F-F_	0	-3.87747900	-2.85089600	-4.92023000	L
C-C_R	0	-3.16262800	-3.71083600	-2.85134800	L
C-C_R	0	-4.13194400	-3.07518000	-1.96402300	L
C-C_R	0	-1.29398700	-5.09859600	-2.66263400	L
C-C_R	0	-1.13080000	-5.01229100	-4.06260500	L
C-C_R	0	-1.99855800	-4.26602600	-4.79635700	L
C-C_R	0	-3.01947400	-3.60668200	-4.18975000	L
C-C_3	0	-5.13060800	-3.43025500	1.54825500	L
C-C_3	0	-5.69106200	-2.52439800	2.67119100	L
C-C_3	0	-4.39616200	-4.63398400	2.18245600	L
C-C_3	0	-6.34285000	-3.97517700	0.75499700	L
C-C_R	0	-2.32463700	-4.43392000	-2.10963500	L
H-H_	0	-2.17690000	3.62473100	2.16632600	L
H-H_	0	-4.79550900	2.56333700	5.46203800	L
H-H_	0	-4.09801400	1.21161400	4.52862800	L
H-H_	0	-5.38238600	2.24485400	3.80563900	L

H-H_	0	-4.37985100	4.97163200	4.81977600	L
H-H_	0	-4.94197200	4.62584300	3.15197500	L
H-H_	0	-3.32515000	5.37243200	3.41817300	L
H-H_	0	-2.58676800	3.66822200	5.88522200	L
H-H_	0	-1.46397900	4.02617600	4.53233200	L
H-H_	0	-1.74829200	2.32723600	5.05677200	L
H-H_	0	3.55529800	0.50824300	2.96414300	L
H-H_	0	4.62921900	1.87401000	6.26302400	L
H-H_	0	4.34830300	2.91670800	4.83330700	L
H-H_	0	5.17841100	1.33004600	4.63744500	L
H-H_	0	2.15541700	1.98947200	6.80004700	L
H-H_	0	0.91606200	1.53271200	5.59607400	L
H-H_	0	1.89318200	3.02452700	5.36350400	L
H-H_	0	3.43154100	-0.20901200	6.61229400	L
H-H_	0	3.85523000	-0.86051300	4.99264900	L
H-H_	0	2.14005200	-0.79088200	5.53369600	L
H-H_	0	0.84286800	-4.79720400	0.62164300	L
H-H_	0	2.04876300	-7.48626300	2.87028200	L
H-H_	0	3.14504100	-6.43500200	1.91592400	L
H-H_	0	1.62149000	-7.04828400	1.17778800	L
H-H_	0	1.93150000	-5.74285600	4.71283900	L
H-H_	0	1.54008500	-4.03783700	4.34978300	L
H-H_	0	3.08547300	-4.75740000	3.77140600	L
H-H_	0	-0.13713400	-6.71059600	3.65604200	L
H-H_	0	-0.65015300	-6.18437300	2.01747800	L
H-H_	0	-0.72106600	-5.04352100	3.40748200	L
H-H_	0	-4.84529300	-1.76246300	0.20729400	L
H-H_	0	-6.42283500	-3.08189900	3.29528200	L
H-H_	0	-6.21548500	-1.64519200	2.24012900	L
H-H_	0	-4.89825400	-2.16167000	3.35367400	L
H-H_	0	-5.06104300	-5.15401300	2.90563400	L
H-H_	0	-3.48523400	-4.32642600	2.73187400	L
H-H_	0	-4.10978000	-5.37794300	1.41111700	L
H-H_	0	-7.04385500	-4.51880600	1.42515300	L
H-H_	0	-6.01949100	-4.68457000	-0.03657800	L
H-H_	0	-6.90106600	-3.14477700	0.27049900	L
N-N_3	0	0.08620100	0.13825100	-0.90548800	H
S-S_3+6	0	0.56309400	-0.89364600	-2.10350500	H
O-O_2	0	-0.01048000	-2.24906900	-2.12027600	H
O-O_R	0	2.19072200	-1.11522600	-1.44678700	H
O-O_2	0	0.70613000	-0.14011700	-3.37055600	H
C-C_R	0	3.22523000	-0.40642900	-2.00470100	H
C-C_R	0	3.65673400	-0.64671900	-3.32395300	H
C-C_R	0	3.91814900	0.50806400	-1.18701900	H

C-C_R	0	4.79396500	0.01175300	-3.80323800	H
H-H_	0	3.12115000	-1.36612400	-3.94483600	H
C-C_R	0	5.03926800	1.16257200	-1.69152600	H
H-H_	0	3.54432800	0.68562300	-0.17744700	H
C-C_R	0	5.51314000	0.92993200	-3.00844000	H
H-H_	0	5.12319300	-0.20672300	-4.82031600	H
H-H_	0	5.56246600	1.87308000	-1.04752200	H
C-C_3	0	6.74774800	1.69011700	-3.52635800	H
C-C_3	0	6.39875500	3.20099000	-3.61769200	H
H-H_	0	6.11417700	3.61405600	-2.63743500	H
H-H_	0	7.26917600	3.77149200	-3.98464800	H
H-H_	0	5.55967500	3.37124700	-4.31190300	H
C-C_3	0	7.19207900	1.20716300	-4.92426900	H
H-H_	0	8.09285800	1.76016800	-5.23731100	H
H-H_	0	7.44349100	0.13332500	-4.92559300	H
H-H_	0	6.41742500	1.38323100	-5.68874000	H
C-C_3	0	7.93881100	1.49977500	-2.55035200	H
H-H_	0	8.81383700	2.06719000	-2.91005100	H
H-H_	0	7.70795400	1.85753500	-1.53452800	H
H-H_	0	8.22839200	0.43911500	-2.47806300	H
C-C_R	0	-0.87857700	3.71638500	-2.91031500	H
C-C_R	0	-1.33530900	5.05086400	-2.86359100	H
C-C_R	0	-0.49663800	6.11726400	-3.21460200	H
C-C_R	0	0.82889800	5.86969900	-3.60538000	H
C-C_R	0	1.30464300	4.54855100	-3.63940700	H
C-C_R	0	0.46070600	3.48412800	-3.29896600	H
H-H_	0	-2.36555700	5.25128700	-2.55302100	H
H-H_	0	-0.87502700	7.14329900	-3.17636600	H
H-H_	0	1.48929200	6.70095200	-3.86964400	H
H-H_	0	2.34052600	4.34772100	-3.92882000	H
H-H_	0	0.83532300	2.45740000	-3.32733900	H
C-C_3	0	-1.79129400	2.56156400	-2.56637900	H
H-H_	0	-2.66292700	2.93827700	-2.00502000	H
H-H_	0	-1.25271800	1.86561600	-1.89084500	H
C-C_3	0	-2.30689500	1.73552400	-3.77933400	H
H-H_	0	-1.42209000	1.33228700	-4.30410300	H
C-C_3	0	-3.14990000	0.54721000	-3.28642500	H
H-H_	0	-4.05280900	0.89751800	-2.75652500	H
H-H_	0	-3.48062800	-0.08187800	-4.12995900	H
H-H_	0	-2.58616600	-0.09427800	-2.58959300	H
C-C_3	0	-3.10339800	2.60078300	-4.77187200	H
H-H_	0	-4.00066700	3.03114100	-4.29313300	H
H-H_	0	-2.49751900	3.43535000	-5.15953200	H
H-H_	0	-3.44330400	1.99815600	-5.63127700	H

^{1}TS2

C-C_R	0	2.24913500	-5.22536200	1.42273500	L
C-C_R	0	-5.02229800	4.10799000	1.15410300	L
C-C_R	0	5.12897700	3.91220300	2.22285200	L
C-C_R	0	-5.50851400	-2.06296200	-1.01596400	L
C-C_3	0	5.25995100	-2.09833400	-2.65791400	L
C-C_3	0	2.36056600	4.64030500	-3.14222600	L
C-C_3	0	-3.65056100	1.91411300	-4.24261200	L
C-C_3	0	-0.92470200	-4.72572200	-3.65890900	L
C-C_3	0	1.04582000	4.73808200	-3.95164600	L
C-C_3	0	6.62156300	-2.62589200	-2.14383400	L
C-C_3	0	-1.46631300	-6.15261000	-3.40396100	L
C-C_3	0	-2.75931100	3.05465000	-4.78714200	L
C-C_3	0	4.59696100	-3.22213000	-3.48783900	L
C-C_3	0	-1.75931200	-4.09968800	-4.80030400	L
C-C_3	0	3.47036300	4.04990200	-4.04343800	L
C-C_3	0	-5.11568800	2.41476900	-4.24251700	L
C-C_3	0	2.77756600	6.08116700	-2.76224400	L
C-C_3	0	0.54577300	-4.86586400	-4.12062500	L
C-C_3	0	-3.58651600	0.70404600	-5.20654100	L
C-C_3	0	5.55901100	-0.88897500	-3.57632500	L
C-C_R	0	-1.84218200	0.92222200	-2.56791400	H
C-C_R	0	-0.38422600	-2.50975400	-2.30092600	H
C-C_R	0	3.00697500	-1.05304700	-1.71148500	H
C-C_R	0	1.55348400	2.40588500	-1.95949300	H
C-C_3	0	-3.25365900	1.48872600	-2.77758700	L
C-C_3	0	-1.00621600	-3.90891800	-2.31529800	L
C-C_3	0	4.38443800	-1.66344600	-1.42286800	L
C-C_3	0	2.13694300	3.81562800	-1.81932300	L
C-C_R	0	-2.61231800	3.63416400	-1.42056000	L
C-C_R	0	-2.84692800	-4.38845100	-0.56489400	L
C-C_R	0	4.78144400	-2.77816400	0.86745600	L
C-C_R	0	4.43344300	2.81399500	-1.09667900	L
C-C_R	0	-3.14541500	4.28511100	-0.22552700	L
C-C_R	0	-4.09470000	-3.73296900	-0.17816900	L
C-C_R	0	4.04228600	-3.72684900	1.69369000	L
C-C_R	0	5.18978300	2.81584100	0.15143400	L
C-C_R	0	-4.30672200	3.70913100	0.08797600	L
C-C_R	0	-4.41704700	-2.84497200	-1.11862700	L
C-C_R	0	3.11971400	-4.31820000	0.93096400	L
C-C_R	0	4.65543000	3.71934200	0.97334300	L
C-C_R	0	-4.57600300	2.65623200	-0.88453700	L
C-C_R	0	-3.39712100	-2.88389700	-2.16190700	L

C-C_R	0	3.22196900	-3.77333600	-0.42117700	L
C-C_R	0	3.52985100	4.35223500	0.29063100	L
C-C_R	0	-2.62885500	5.29084900	0.51154100	L
C-C_R	0	-4.84269300	-3.89853500	0.92953400	L
C-C_R	0	4.16408200	-4.00029400	3.00487000	L
C-C_R	0	6.23313800	2.05497200	0.52866500	L
C-C_R	0	-3.31139400	5.72557800	1.60260900	L
C-C_R	0	-6.00199200	-3.10688600	1.08044600	L
C-C_R	0	3.28208400	-4.94461800	3.56449200	L
C-C_R	0	6.75102300	2.24683500	1.82831900	L
C-C_R	0	-4.49872800	5.15638200	1.94212200	L
C-C_R	0	-6.32939800	-2.20449000	0.12330600	L
C-C_R	0	2.31364900	-5.55469000	2.73489200	L
C-C_R	0	6.18485200	3.17137800	2.64878100	L
F-F_	0	-1.44435100	5.85916500	0.17159200	L
F-F_	0	-4.49065800	-4.79626200	1.88347900	L
F-F_	0	5.10290400	-3.38775300	3.76816600	L
F-F_	0	6.76848300	1.12869800	-0.30512100	L
F-F_	0	-2.80490300	6.73223200	2.35799800	L
F-F_	0	-6.77878200	-3.24483800	2.18359600	L
F-F_	0	3.35643800	-5.25851400	4.88152900	L
F-F_	0	7.80015100	1.50243900	2.25806400	L
F-F_	0	-5.17982700	5.59268400	3.03086800	L
F-F_	0	-7.43768500	-1.43510400	0.25949700	L
F-F_	0	1.44808000	-6.46058100	3.25427600	L
F-F_	0	6.67228100	3.34967800	3.90217700	L
F-F_	0	-6.21370600	3.53557800	1.45735500	L
F-F_	0	-5.81880500	-1.16338200	-1.98235600	L
F-F_	0	1.31463300	-5.79331400	0.61908000	L
F-F_	0	4.56055300	4.83011500	3.04420600	L
H-H_	0	-1.38473900	-6.77746500	-4.31990900	L
H-H_	0	-3.07349300	3.33123000	-5.81682700	L
H-H_	0	1.17803800	5.39784900	-4.83644800	L
H-H_	0	7.29217200	-2.88676300	-2.99133700	L
H-H_	0	-2.53689200	-6.12706100	-3.10780400	L
H-H_	0	6.49223600	-3.54144400	-1.52796600	L
H-H_	0	-1.69193300	2.76543900	-4.83619900	L
H-H_	0	0.23292200	5.16621500	-3.32773700	L
H-H_	0	-0.89362100	-6.65307300	-2.59321400	L
H-H_	0	7.13168500	-1.85761200	-1.52302400	L
H-H_	0	-2.84724800	3.96418300	-4.15790100	L
H-H_	0	0.71621600	3.74957200	-4.32827200	L
H-H_	0	-1.63599500	-4.68868700	-5.73489300	L
H-H_	0	5.23463300	-3.48531200	-4.35937300	L

H-H_	0	3.57447700	4.65356100	-4.97100000	L
H-H_	0	-5.45394600	2.65536300	-5.27388000	L
H-H_	0	-1.45208800	-3.06021100	-5.02443400	L
H-H_	0	3.60627200	-2.92333400	-3.88189200	L
H-H_	0	3.25250600	3.01006400	-4.35334100	L
H-H_	0	-5.22619000	3.33842300	-3.63540700	L
H-H_	0	-2.84084500	-4.10508700	-4.55332500	L
H-H_	0	4.47117300	-4.14309500	-2.88207300	L
H-H_	0	4.45390000	4.06896600	-3.53048400	L
H-H_	0	-5.79582700	1.63929100	-3.82802300	L
H-H_	0	6.26948700	-1.17574700	-4.38177300	L
H-H_	0	0.61677100	-5.53344600	-5.00661800	L
H-H_	0	2.90170100	6.71077600	-3.67012400	L
H-H_	0	-3.98789800	0.97841300	-6.20622800	L
H-H_	0	6.02092400	-0.06222600	-2.99588800	L
H-H_	0	1.17011600	-5.30480700	-3.31350000	L
H-H_	0	-4.19466900	-0.13967200	-4.81609500	L
H-H_	0	3.74384900	6.08610400	-2.21385600	L
H-H_	0	4.64952700	-0.50313600	-4.07580100	L
H-H_	0	2.00881100	6.55730400	-2.11569400	L
H-H_	0	-2.55213100	0.34534900	-5.36903600	L
H-H_	0	0.98505100	-3.89140700	-4.41224300	L
H-H_	0	-3.93326700	0.65230900	-2.50478400	L
H-H_	0	-0.40203900	-4.48990900	-1.58229800	L
H-H_	0	4.94852700	-0.84566300	-0.92162000	L
H-H_	0	1.36716700	4.38052000	-1.24573400	L
N-N_R	0	-3.50254200	2.58609100	-1.82736500	L
N-N_R	0	-2.38708700	-3.83848900	-1.80561800	L
N-N_R	0	4.24743000	-2.77048700	-0.45970700	L
N-N_R	0	3.36237300	3.76437000	-1.00408000	L
O-O_R	0	-1.55936600	0.59179600	-1.36021100	H
O-O_R	0	-0.25231100	-2.00615200	-1.13048800	H
O-O_R	0	2.34969100	-0.68481300	-0.66750100	H
O-O_R	0	1.09057800	1.90292200	-0.87093700	H
O-O_R	0	-1.06009800	0.80711300	-3.56688600	H
O-O_R	0	-0.06833100	-1.93907500	-3.39739300	H
O-O_R	0	2.60073700	-0.94234800	-2.91128500	H
O-O_R	0	1.58811000	1.82155300	-3.08965600	H
O-O_R	0	-1.41654400	3.72311900	-1.80369400	L
O-O_R	0	-2.09705700	-5.00544300	0.23829500	L
O-O_R	0	5.58046500	-1.90875100	1.30527700	L
O-O_R	0	4.46163500	1.87663200	-1.93693600	L
O-O_R	0	-5.48603800	1.79047900	-0.78697500	L
O-O_R	0	-3.22049200	-1.96857500	-3.00827600	L

O-O_R	0	2.32592200	-3.88694100	-1.29810600	L
O-O_R	0	2.65021100	5.05335800	0.85757300	L
Rh-	0	0.39439300	-0.06156300	-0.88268400	H
Rh-	0	0.77306500	-0.06279700	-3.28287000	H
N-N_3	0	-0.15279100	0.01082200	1.02143300	H
S-S_3+6	0	-0.25781100	-1.38518900	1.95655900	H
O-O_2	0	0.55950100	-2.54622600	1.57329000	H
O-O_R	0	-1.85136300	-1.78765500	1.39057400	H
O-O_2	0	-0.36587300	-0.99311700	3.38019200	H
C-C_R	0	-2.92680700	-1.28287500	2.10666500	H
C-C_R	0	-3.29780400	-1.85990000	3.33107000	H
C-C_R	0	-3.69032500	-0.24568500	1.54652500	H
C-C_R	0	-4.44678200	-1.40083500	3.98788600	H
H-H_	0	-2.69949500	-2.67275900	3.74589400	H
C-C_R	0	-4.82167700	0.20572100	2.22768000	H
H-H_	0	-3.36917500	0.19354200	0.60007700	H
C-C_R	0	-5.23234900	-0.35639700	3.46035900	H
H-H_	0	-4.72400000	-1.87441900	4.93132300	H
H-H_	0	-5.40022400	1.02241000	1.79187000	H
C-C_3	0	-6.46891300	0.20777700	4.18662800	H
C-C_3	0	-6.15189100	1.65573000	4.64979700	H
H-H_	0	-5.88951900	2.30673500	3.80113400	H
H-H_	0	-7.02604300	2.09738900	5.15882400	H
H-H_	0	-5.30299000	1.66653300	5.35310400	H
C-C_3	0	-6.85275100	-0.62751900	5.42764400	H
H-H_	0	-7.75761600	-0.20432900	5.89437800	H
H-H_	0	-7.07267600	-1.67527700	5.16246100	H
H-H_	0	-6.05757900	-0.62339600	6.19140100	H
C-C_3	0	-7.68879500	0.22919700	3.22916400	H
H-H_	0	-8.56571900	0.65873000	3.74296800	H
H-H_	0	-7.50361100	0.83588700	2.32865300	H
H-H_	0	-7.95219700	-0.78964200	2.90174000	H
C-C_R	0	2.45493400	0.87309500	3.42707200	H
C-C_R	0	3.75844500	0.56547700	2.96635500	H
C-C_R	0	4.68126900	-0.08742700	3.78713900	H
C-C_R	0	4.30995700	-0.48656100	5.08203900	H
C-C_R	0	3.00793300	-0.22999200	5.54252900	H
C-C_R	0	2.09084800	0.43920700	4.72706200	H
H-H_	0	4.03480600	0.85011300	1.94851800	H
H-H_	0	5.69335700	-0.28520400	3.42461300	H
H-H_	0	5.02829500	-1.00453800	5.72367200	H
H-H_	0	2.70708600	-0.55514600	6.54226000	H
H-H_	0	1.07973900	0.62261600	5.09308200	H
C-C_3	0	1.52613200	1.63980800	2.54913200	H

H-H_	0	0.85373500	0.87923500	1.93349100	H
C-C_3	0	0.51936200	2.60403900	3.20598900	H
C-C_3	0	1.25440600	3.70314800	4.00647900	H
H-H_	0	1.87830000	4.32354200	3.34175500	H
H-H_	0	0.52363100	4.36942800	4.49528800	H
H-H_	0	1.90840900	3.27799500	4.78376900	H
C-C_3	0	-0.42112800	3.22839700	2.16187800	H
H-H_	0	0.14381400	3.81787200	1.41989200	H
H-H_	0	-0.98568000	2.45600100	1.61944900	H
H-H_	0	-1.13912700	3.90371300	2.65611000	H
H-H_	0	-0.10271900	2.02153500	3.90894200	H
H-H_	0	2.07808000	2.13564600	1.73222200	H

^{3}INT3

C-C_R	0	5.82210200	-1.39413200	0.49133300	L
C-C_R	0	-5.64370700	3.60547100	-1.83309000	L
C-C_R	0	-3.01043600	-4.50021900	-3.34537700	L
C-C_R	0	1.10262800	5.81537100	0.69414800	L
C-C_3	0	1.90786200	-4.93561700	3.12815700	L
C-C_3	0	-4.81009000	-3.36666500	2.39170800	L
C-C_3	0	-3.32067000	3.46962000	3.69922100	L
C-C_3	0	3.39673100	1.85865400	4.40839500	L
C-C_3	0	-5.31254600	-2.29016800	3.38402700	L
C-C_3	0	2.70404700	-6.19841300	2.71998000	L
C-C_3	0	4.73377500	2.63804500	4.42949500	L
C-C_3	0	-4.32203100	2.47371100	4.33042500	L
C-C_3	0	2.67985000	-4.23844300	4.27277900	L
C-C_3	0	2.36933100	2.65894000	5.24313100	L
C-C_3	0	-4.00526600	-4.43784900	3.16334100	L
C-C_3	0	-4.04547400	4.82746400	3.53471300	L
C-C_3	0	-6.05968400	-4.05269500	1.78901000	L
C-C_3	0	3.65552800	0.49213700	5.08716900	L
C-C_3	0	-2.13991900	3.69847600	4.67297700	L
C-C_3	0	0.53270500	-5.41147700	3.65537300	L
C-C_R	0	-1.94919900	1.72052700	2.24921700	H
C-C_R	0	1.67974100	0.83653800	2.65693800	H
C-C_R	0	0.87105500	-2.75983400	2.01429400	H
C-C_R	0	-2.74586600	-1.88769500	1.60556400	H
C-C_3	0	-2.80124600	2.99581100	2.28898700	L
C-C_3	0	2.94656800	1.66194900	2.91110000	L
C-C_3	0	1.72904300	-4.01899800	1.85960600	L
C-C_3	0	-3.98452600	-2.70636000	1.22496800	L
C-C_R	0	-4.69678900	1.72229200	1.02428500	L
C-C_R	0	3.67745100	3.39707100	1.14545100	L

C-C_R	0	3.51536400	-4.07310500	-0.01281000	L
C-C_R	0	-2.44995800	-4.52647900	0.17422600	L
C-C_R	0	-5.41034700	1.92286300	-0.23161100	L
C-C_R	0	3.07210000	4.55972200	0.50072800	L
C-C_R	0	4.63780000	-3.22167100	-0.40031500	L
C-C_R	0	-2.14050900	-4.89027100	-1.20424700	L
C-C_R	0	-5.12463100	3.14519700	-0.68096800	L
C-C_R	0	1.90216800	4.80778600	1.09131600	L
C-C_R	0	4.86125300	-2.33877200	0.57660300	L
C-C_R	0	-3.06787100	-4.36455700	-2.00325700	L
C-C_R	0	-4.20261700	3.78465600	0.25647600	L
C-C_R	0	1.71209900	3.83922200	2.16640100	L
C-C_R	0	3.89527400	-2.57986600	1.64497400	L
C-C_R	0	-4.02805600	-3.63766200	-1.17741800	L
C-C_R	0	-6.21816000	1.08308800	-0.91266100	L
C-C_R	0	3.52352500	5.30608400	-0.52449000	L
C-C_R	0	5.36108100	-3.22471900	-1.53445600	L
C-C_R	0	-1.10000200	-5.58452400	-1.69916900	L
C-C_R	0	-6.76671400	1.50238700	-2.08210800	L
C-C_R	0	2.72295200	6.37778800	-0.97633000	L
C-C_R	0	6.38452500	-2.26667400	-1.66581900	L
C-C_R	0	-1.02366300	-5.74594400	-3.09989900	L
C-C_R	0	-6.50301100	2.74889500	-2.55638100	L
C-C_R	0	1.53300600	6.62864600	-0.37669600	L
C-C_R	0	6.59815300	-1.34316200	-0.61749500	L
C-C_R	0	-1.98236900	-5.19867000	-3.89344800	L
F-F_	0	-6.47997900	-0.16117700	-0.43915900	L
F-F_	0	4.71307700	5.03498300	-1.11606700	L
F-F_	0	5.11319500	-4.11732700	-2.52513200	L
F-F_	0	-0.14877500	-6.10545900	-0.88446300	L
F-F_	0	-7.58002700	0.67217800	-2.78186800	L
F-F_	0	3.14228200	7.14922900	-2.01002600	L
F-F_	0	7.14595800	-2.22523600	-2.78668500	L
F-F_	0	0.00670700	-6.43625300	-3.64892500	L
F-F_	0	-7.05254800	3.16626400	-3.72400200	L
F-F_	0	0.75430100	7.65244700	-0.80674500	L
F-F_	0	7.56884500	-0.40244200	-0.72779700	L
F-F_	0	-1.90847900	-5.34540500	-5.24015000	L
F-F_	0	-5.35010100	4.84773100	-2.29152600	L
F-F_	0	-0.08588700	6.04801600	1.30562400	L
F-F_	0	6.00809600	-0.50387100	1.49842800	L
F-F_	0	-3.96170500	-3.94566600	-4.13813800	L
H-H_	0	5.11381100	2.74641800	5.46867000	L
H-H_	0	-4.66738000	2.84868800	5.31819100	L

H-H_	0	-5.99503800	-2.74002300	4.13741200	L
H-H_	0	2.80541500	-6.89819800	3.57793500	L
H-H_	0	4.60984100	3.65980300	4.01125200	L
H-H_	0	3.72828800	-5.93428600	2.38008300	L
H-H_	0	-3.87478600	1.47531100	4.49825200	L
H-H_	0	-5.87438200	-1.49519200	2.84872600	L
H-H_	0	5.50774200	2.10839000	3.83266100	L
H-H_	0	2.19174500	-6.73733200	1.89359600	L
H-H_	0	-5.22081300	2.35371900	3.69079800	L
H-H_	0	-4.48309600	-1.81666100	3.94456000	L
H-H_	0	2.71455200	2.74824400	6.29592000	L
H-H_	0	2.74350000	-4.90802500	5.15773800	L
H-H_	0	-4.61095300	-4.84529500	4.00157700	L
H-H_	0	-4.36905700	5.22688200	4.52053000	L
H-H_	0	1.37424200	2.17459200	5.26379900	L
H-H_	0	2.18892000	-3.30295100	4.60294700	L
H-H_	0	-3.07249700	-4.02905700	3.59786800	L
H-H_	0	-4.95354900	4.72440000	2.90327800	L
H-H_	0	2.25141600	3.68941200	4.84970700	L
H-H_	0	3.72013300	-4.00325700	3.96787900	L
H-H_	0	-3.74386800	-5.29121300	2.50430000	L
H-H_	0	-3.37485400	5.57766400	3.06228600	L
H-H_	0	0.66186900	-6.14143100	4.48375600	L
H-H_	0	4.06885900	0.63477800	6.10920900	L
H-H_	0	-6.69706000	-4.48951700	2.58837300	L
H-H_	0	-2.50294800	4.11801800	5.63625500	L
H-H_	0	-0.04374900	-5.91272700	2.84901800	L
H-H_	0	4.39214000	-0.10048300	4.50418800	L
H-H_	0	-1.41313700	4.41941500	4.24227600	L
H-H_	0	-5.77283300	-4.87695100	1.10144900	L
H-H_	0	-0.07526600	-4.57549800	4.05325800	L
H-H_	0	-6.67320600	-3.32193800	1.21852400	L
H-H_	0	-1.60338600	2.75971700	4.91220700	L
H-H_	0	2.72998900	-0.10610200	5.19510200	L
H-H_	0	-2.12167900	3.80049900	1.92808400	L
H-H_	0	3.76253600	1.08421300	2.42062800	L
H-H_	0	1.18107600	-4.64178000	1.11777700	L
H-H_	0	-4.67106800	-1.96658200	0.75673300	L
N-N_R	0	-3.91446700	2.88293200	1.33111700	L
N-N_R	0	2.83790800	2.95389000	2.21459900	L
N-N_R	0	3.02974100	-3.65916400	1.26888500	L
N-N_R	0	-3.62245600	-3.69797700	0.19594900	L
O-O_R	0	-1.38745900	1.46895200	1.12086600	H
O-O_R	0	1.42016800	0.61638300	1.41728500	H

O-O_R	0	0.62079500	-2.15040700	0.91503400	H
O-O_R	0	-2.18060800	-1.28994400	0.61909300	H
O-O_R	0	-1.85628300	0.98979600	3.28916900	H
O-O_R	0	0.97287500	0.44100500	3.63929500	H
O-O_R	0	0.47812500	-2.38885800	3.17001600	H
O-O_R	0	-2.35138500	-1.84979400	2.81693400	H
O-O_R	0	-4.62382600	0.63367800	1.65165800	L
O-O_R	0	4.88466800	3.05763000	1.02628100	L
O-O_R	0	2.83527800	-4.75820900	-0.82274100	L
O-O_R	0	-1.61275200	-4.56789700	1.11342900	L
O-O_R	0	-3.50407100	4.79949100	-0.00788200	L
O-O_R	0	0.60636500	3.59379500	2.71521600	L
O-O_R	0	3.60872900	-1.74013600	2.53789600	L
O-O_R	0	-4.85042200	-2.79301900	-1.62215800	L
Rh-	0	-0.36449900	-0.33045200	0.90187600	H
Rh-	0	-0.69675800	-0.70532100	3.26473700	H
N-N_3	0	-0.08050900	-0.20452200	-1.03266600	H
S-S_3+6	0	0.60796400	-1.21933000	-2.12255000	H
O-O_2	0	0.61964900	-0.55884400	-3.44209000	H
O-O_R	0	2.23846000	-1.26387400	-1.53612900	H
O-O_2	0	0.13876700	-2.60836400	-1.97810700	H
C-C_R	0	3.19054200	-0.48004100	-2.16973300	H
C-C_R	0	3.77589600	0.57211500	-1.44896100	H
C-C_R	0	3.64163400	-0.79464500	-3.46534500	H
C-C_R	0	4.82656400	1.29605000	-2.02358700	H
H-H_	0	3.39862300	0.80114600	-0.45067400	H
C-C_R	0	4.69320000	-0.06090000	-4.01596400	H
H-H_	0	3.18240600	-1.61884800	-4.01320400	H
C-C_R	0	5.31662300	0.99737900	-3.31347800	H
H-H_	0	5.27306100	2.10854300	-1.44953500	H
H-H_	0	5.04045400	-0.32733300	-5.01770700	H
C-C_3	0	6.47677700	1.77415100	-3.96432000	H
C-C_3	0	5.96657800	2.45411700	-5.26321100	H
H-H_	0	5.58821400	1.71837900	-5.99074700	H
H-H_	0	6.78491500	3.01464900	-5.74682300	H
H-H_	0	5.14927600	3.16068400	-5.04415300	H
C-C_3	0	7.04746000	2.86800800	-3.03607800	H
H-H_	0	7.88438000	3.38002200	-3.53877600	H
H-H_	0	7.43525900	2.44697300	-2.09332800	H
H-H_	0	6.29350700	3.63363200	-2.79096300	H
C-C_3	0	7.62682200	0.79266400	-4.31450100	H
H-H_	0	8.44794900	1.33403500	-4.81467000	H
H-H_	0	7.29482400	-0.01020400	-4.99165700	H
H-H_	0	8.03458200	0.32064700	-3.40588400	H

C-C_R	0	-3.04350600	2.09568400	-3.49654300	H
C-C_R	0	-3.76470000	2.56637000	-4.61272000	H
C-C_R	0	-4.56976600	1.70427300	-5.37147800	H
C-C_R	0	-4.67042200	0.34837500	-5.02497500	H
C-C_R	0	-3.96067000	-0.13456200	-3.91515100	H
C-C_R	0	-3.16117700	0.73145600	-3.15732600	H
H-H_	0	-3.70467500	3.62655800	-4.87965500	H
H-H_	0	-5.12554400	2.09453500	-6.22956200	H
H-H_	0	-5.30155200	-0.32556000	-5.61164000	H
H-H_	0	-4.03071700	-1.18932700	-3.63721400	H
H-H_	0	-2.62177200	0.34933300	-2.28535800	H
C-C_3	0	-2.16665400	3.01771000	-2.67321000	H
H-H_	0	-2.57306900	4.04660600	-2.70604900	H
H-H_	0	-2.20164600	2.69228300	-1.61859100	H
C-C_3	0	-0.67109600	3.06648000	-3.09578700	H
H-H_	0	-0.29680100	2.02766200	-3.09715800	H
C-C_3	0	0.13177100	3.86464500	-2.05450200	H
H-H_	0	-0.22470500	4.90940000	-2.00148000	H
H-H_	0	1.20450600	3.89002200	-2.31084800	H
H-H_	0	0.03472000	3.42205400	-1.04906300	H
C-C_3	0	-0.47703900	3.64764100	-4.50544300	H
H-H_	0	-0.82978100	4.69439200	-4.55541900	H
H-H_	0	-1.02812500	3.06757600	-5.26269100	H
H-H_	0	0.58946400	3.64177800	-4.78771300	H

^{3}TS3

C-C_R	0	-5.74095900	-1.48056500	-0.57352300	L
C-C_R	0	5.26573400	3.67276000	2.41886600	L
C-C_R	0	3.25684300	-4.86715100	2.84633300	L
C-C_R	0	-1.33110600	5.87653600	-0.16677300	L
C-C_3	0	-1.70707100	-4.52350400	-3.63944000	L
C-C_3	0	4.96946800	-2.75561400	-2.60083700	L
C-C_3	0	3.21599800	4.08102300	-3.23613300	L
C-C_3	0	-3.44924400	2.19559900	-4.29306400	L
C-C_3	0	5.44088700	-1.53849700	-3.43253900	L
C-C_3	0	-2.46056200	-5.85257300	-3.39153300	L
C-C_3	0	-4.82962700	2.89593900	-4.28069800	L
C-C_3	0	4.27093400	3.19465700	-3.93934300	L
C-C_3	0	-2.49809400	-3.72029600	-4.69827200	L
C-C_3	0	-2.46037800	3.11217200	-5.05127900	L
C-C_3	0	4.24043400	-3.75746300	-3.52606600	L
C-C_3	0	3.88054900	5.44170200	-2.91481000	L
C-C_3	0	6.23750300	-3.45249100	-2.05134400	L
C-C_3	0	-3.61835500	0.86894500	-5.07183900	L

C-C_3	0	2.04880500	4.36350400	-4.21206800	L
C-C_3	0	-0.31415800	-4.88731900	-4.20819600	L
C-C_R	0	1.89288300	2.13489300	-2.00327100	H
C-C_R	0	-1.70226700	1.14544200	-2.58764300	H
C-C_R	0	-0.76942700	-2.44907000	-2.27052000	H
C-C_R	0	2.81080400	-1.49241200	-1.70286700	H
C-C_3	0	2.68534900	3.44574800	-1.89533300	L
C-C_3	0	-3.00794600	1.91959000	-2.80574500	L
C-C_3	0	-1.56711800	-3.75782000	-2.26985000	L
C-C_3	0	4.08046500	-2.29152600	-1.38689300	L
C-C_R	0	4.62547500	2.14997900	-0.72643200	L
C-C_R	0	-3.85895500	3.49514100	-0.94299500	L
C-C_R	0	-3.34701200	-4.12595400	-0.42770000	L
C-C_R	0	2.61100000	-4.29992600	-0.61324000	L
C-C_R	0	5.25233000	2.22315600	0.58873600	L
C-C_R	0	-3.27613500	4.56985400	-0.14600700	L
C-C_R	0	-4.49422900	-3.36985400	0.06937400	L
C-C_R	0	2.34806200	-4.91198800	0.68565200	L
C-C_R	0	4.86474900	3.35621800	1.17465400	L
C-C_R	0	-2.11752500	4.92437500	-0.70345000	L
C-C_R	0	-4.75271800	-2.37591200	-0.78424000	L
C-C_R	0	3.26002400	-4.47629400	1.55444800	L
C-C_R	0	3.96048800	4.06536600	0.27129200	L
C-C_R	0	-1.89418600	4.09218200	-1.88286800	L
C-C_R	0	-3.78796200	-2.44615400	-1.87794300	L
C-C_R	0	4.13690000	-3.53821500	0.86089800	L
C-C_R	0	6.05858100	1.33780600	1.21187600	L
C-C_R	0	-3.72576100	5.14161300	0.98660600	L
C-C_R	0	-5.20891100	-3.54311400	1.19567100	L
C-C_R	0	1.37239800	-5.75904400	1.05904400	L
C-C_R	0	6.49637600	1.61655400	2.46681300	L
C-C_R	0	-2.94040900	6.15115800	1.58442700	L
C-C_R	0	-6.26038800	-2.64299000	1.45375100	L
C-C_R	0	1.34943600	-6.18232600	2.40618000	L
C-C_R	0	6.12058700	2.76727400	3.08541000	L
C-C_R	0	-1.76365800	6.51352100	1.01658400	L
C-C_R	0	-6.50957700	-1.59791500	0.53533900	L
C-C_R	0	2.29494800	-5.72723700	3.27109600	L
F-F_	0	6.42206600	0.18281900	0.59948900	L
F-F_	0	-4.90050800	4.75793000	1.54507400	L
F-F_	0	-4.92593800	-4.54811700	2.06144400	L
F-F_	0	0.43861600	-6.19198500	0.17543400	L
F-F_	0	7.30436300	0.73712600	3.11038200	L
F-F_	0	-3.36014800	6.75027200	2.72653500	L

F-F_	0	-7.01525300	-2.77251100	2.57230500	L
F-F_	0	0.38573100	-7.03570800	2.83354800	L
F-F_	0	6.55027700	3.03903900	4.34277600	L
F-F_	0	-0.99756900	7.47576200	1.58814600	L
F-F_	0	-7.50794800	-0.71047800	0.76965200	L
F-F_	0	2.27320900	-6.12846700	4.56691600	L
F-F_	0	4.85494400	4.81608900	3.02181800	L
F-F_	0	-0.14672900	6.20983400	-0.73841900	L
F-F_	0	-5.96166600	-0.47358500	-1.45595700	L
F-F_	0	4.19109300	-4.40082000	3.71268500	L
H-H_	0	-5.20375300	3.05444100	-5.31559100	L
H-H_	0	4.61924400	3.68057000	-4.87642500	L
H-H_	0	6.15767600	-1.85675600	-4.22036300	L
H-H_	0	-2.53033400	-6.44978200	-4.32669600	L
H-H_	0	-4.77083900	3.89128100	-3.79066200	L
H-H_	0	-3.49646300	-5.66666800	-3.03562900	L
H-H_	0	3.86997900	2.20084800	-4.21611100	L
H-H_	0	5.95595500	-0.79760500	-2.78465800	L
H-H_	0	-5.57773500	2.28226700	-3.73333600	L
H-H_	0	-1.93530900	-6.46646800	-2.62795100	L
H-H_	0	5.16159800	3.04753400	-3.29421400	L
H-H_	0	4.60197300	-1.03117900	-3.94745300	L
H-H_	0	-2.80018200	3.26194200	-6.09896600	L
H-H_	0	-2.53829400	-4.28189200	-5.65666400	L
H-H_	0	4.89205500	-4.03535100	-4.38261900	L
H-H_	0	4.21103200	5.95195700	-3.84567500	L
H-H_	0	-1.43977200	2.68598700	-5.09517900	L
H-H_	0	-2.03633900	-2.73711100	-4.91176600	L
H-H_	0	3.30569600	-3.33792000	-3.94555700	L
H-H_	0	4.77645100	5.30950200	-2.27140100	L
H-H_	0	-2.40363800	4.11468800	-4.57970600	L
H-H_	0	-3.54571100	-3.55478100	-4.37319300	L
H-H_	0	3.99460100	-4.69382500	-2.98406400	L
H-H_	0	3.16860000	6.11326300	-2.38763900	L
H-H_	0	-0.41510600	-5.52274200	-5.11472400	L
H-H_	0	-4.01847400	1.06184400	-6.09087800	L
H-H_	0	6.91761500	-3.74942600	-2.87918300	L
H-H_	0	2.41584100	4.89309600	-5.11790200	L
H-H_	0	0.27796700	-5.45611500	-3.46007600	L
H-H_	0	-4.33305800	0.19787100	-4.55009800	L
H-H_	0	1.28442900	5.00873900	-3.72949400	L
H-H_	0	5.97700200	-4.37276500	-1.48598300	L
H-H_	0	0.26496100	-3.99178200	-4.50640200	L
H-H_	0	6.79676800	-2.77326200	-1.37187100	L

H-H_	0	1.55590000	3.43470000	-4.55942600	L
H-H_	0	-2.65930100	0.33016200	-5.19889400	L
H-H_	0	1.95811600	4.17877300	-1.47892200	L
H-H_	0	-3.79707100	1.26449700	-2.37327200	L
H-H_	0	-0.98927900	-4.43738400	-1.60448500	L
H-H_	0	4.71312600	-1.58836200	-0.79955300	L
N-N_R	0	3.77924100	3.29108000	-0.92020900	L
N-N_R	0	-2.98043800	3.16631500	-2.02276500	L
N-N_R	0	-2.88304600	-3.53365200	-1.64603900	L
N-N_R	0	3.74371500	-3.42513200	-0.50975300	L
O-O_R	0	1.33603300	1.74158000	-0.91190700	H
O-O_R	0	-1.45287300	0.83127200	-1.36612800	H
O-O_R	0	-0.59458700	-1.93663000	-1.10943000	H
O-O_R	0	2.18803400	-1.04372300	-0.67316400	H
O-O_R	0	1.84398900	1.52237000	-3.11713300	H
O-O_R	0	-0.95538200	0.87614600	-3.58251200	H
O-O_R	0	-0.34922700	-1.95626000	-3.37007700	H
O-O_R	0	2.44495100	-1.32768500	-2.91223500	H
O-O_R	0	4.58167400	1.11493700	-1.44118400	L
O-O_R	0	-4.83057300	2.77703500	-0.58698600	L
O-O_R	0	-2.63988200	-4.88728500	0.28527200	L
O-O_R	0	1.77778500	-4.25381800	-1.55567400	L
O-O_R	0	3.17328000	4.98168500	0.62986500	L
O-O_R	0	-0.77136000	3.93240900	-2.42910700	L
O-O_R	0	-3.53993000	-1.49241500	-2.66082600	L
O-O_R	0	5.22186300	-3.08739100	1.31545400	L
Rh-	0	0.34966200	-0.11378400	-0.89362100	H
Rh-	0	0.75379600	-0.22040500	-3.28040700	H
N-N_3	0	0.03215900	-0.09863400	1.10899400	H
S-S_3+6	0	-0.37927000	-1.45083800	2.01071800	H
O-O_2	0	-0.37680800	-1.11719600	3.44709100	H
O-O_R	0	-2.00775200	-1.70205300	1.53484700	H
O-O_2	0	0.30920600	-2.64731900	1.51044800	H
C-C_R	0	-2.99895700	-0.99673200	2.21963500	H
C-C_R	0	-3.58303000	0.12396900	1.61544300	H
C-C_R	0	-3.46920100	-1.46535600	3.45662500	H
C-C_R	0	-4.64665300	0.77294900	2.26014000	H
H-H_	0	-3.20764700	0.46646700	0.64850000	H
C-C_R	0	-4.53433000	-0.80637000	4.07723500	H
H-H_	0	-3.00526600	-2.34273200	3.91032300	H
C-C_R	0	-5.15026400	0.32683800	3.49912200	H
H-H_	0	-5.09265400	1.64306200	1.77720300	H
H-H_	0	-4.89166500	-1.19077800	5.03660100	H
C-C_3	0	-6.31548500	1.02755400	4.22669800	H

C-C_3	0	-5.81775500	1.54571700	5.60230800	H
H-H_	0	-5.45054500	0.72613500	6.24056300	H
H-H_	0	-6.63787800	2.05004200	6.14191000	H
H-H_	0	-4.99446100	2.26845800	5.47762000	H
C-C_3	0	-6.86833500	2.22885100	3.42944600	H
H-H_	0	-7.70594600	2.68649100	3.98132200	H
H-H_	0	-7.24971700	1.92585600	2.43991200	H
H-H_	0	-6.10464000	3.01030600	3.28241700	H
C-C_3	0	-7.47539200	0.02134500	4.44683400	H
H-H_	0	-8.29711300	0.50272800	5.00442400	H
H-H_	0	-7.15317800	-0.86126200	5.02191100	H
H-H_	0	-7.87975100	-0.33271400	3.48483000	H
C-C_R	0	2.70358900	1.43424800	3.35211700	H
C-C_R	0	3.12926900	1.86529500	4.62423300	H
C-C_R	0	3.95102100	1.05848900	5.42416300	H
C-C_R	0	4.35948200	-0.20086300	4.96201600	H
C-C_R	0	3.94391100	-0.64104300	3.69666200	H
C-C_R	0	3.12939400	0.17078100	2.89501500	H
H-H_	0	2.82358000	2.85294100	4.98561300	H
H-H_	0	4.27632400	1.41728400	6.40514500	H
H-H_	0	5.00160900	-0.83380300	5.58113700	H
H-H_	0	4.25728300	-1.62127200	3.33087800	H
H-H_	0	2.82811500	-0.17727800	1.90265900	H
C-C_3	0	1.82965300	2.32154000	2.48832600	H
H-H_	0	2.01241700	3.38181300	2.76447900	H
H-H_	0	2.11802100	2.22483100	1.42937700	H
C-C_3	0	0.30896200	2.10853500	2.57054100	H
H-H_	0	0.15465700	0.97212400	1.91215100	H
C-C_3	0	-0.47226700	3.07461900	1.69313700	H
H-H_	0	-0.43264300	4.08912100	2.13685100	H
H-H_	0	-1.53302400	2.78575800	1.62651800	H
H-H_	0	-0.05518300	3.12979200	0.67686000	H
C-C_3	0	-0.27882900	1.91500500	3.95330900	H
H-H_	0	-0.19549500	2.86603400	4.51917700	H
H-H_	0	0.24439500	1.13607000	4.52338600	H
H-H_	0	-1.34699500	1.65459100	3.90193400	H

^{3}INT4

C-C_R	0	1.21467100	-5.54027000	1.38848700	L
C-C_R	0	-4.26788100	4.68284000	1.23804200	L
C-C_R	0	6.14137300	3.58206700	1.71730700	L
C-C_R	0	-5.86953800	-1.21286000	-1.01707500	L
C-C_3	0	4.82488800	-2.92726900	-2.51839200	L
C-C_3	0	2.92338900	4.15295500	-3.26331600	L

C-C_3	0	-3.37608300	2.40383200	-4.26984300	L
C-C_3	0	-1.74209200	-4.59859300	-3.56746300	L
C-C_3	0	1.61961500	4.41644700	-4.05276700	L
C-C_3	0	6.07026500	-3.64347500	-1.94220400	L
C-C_3	0	-2.51545500	-5.90941000	-3.28766500	L
C-C_3	0	-2.30119100	3.36751100	-4.82311500	L
C-C_3	0	4.03078900	-3.95560400	-3.35712500	L
C-C_3	0	-2.45696600	-3.86582300	-4.72659300	L
C-C_3	0	3.91735900	3.38413600	-4.16625000	L
C-C_3	0	-4.73408600	3.14735500	-4.28221900	L
C-C_3	0	3.55128600	5.52947000	-2.93852400	L
C-C_3	0	-0.31492700	-4.99310300	-4.01716600	L
C-C_3	0	-3.51716400	1.18688900	-5.21645400	L
C-C_3	0	5.33703600	-1.79906600	-3.44540400	L
C-C_R	0	-1.76672900	1.13838800	-2.57935900	H
C-C_R	0	-0.85154400	-2.47025600	-2.25405300	H
C-C_R	0	2.73202900	-1.52081100	-1.67199000	H
C-C_R	0	1.81552600	2.09925900	-2.00617200	H
C-C_3	0	-3.05865700	1.94044600	-2.79839300	L
C-C_3	0	-1.68986500	-3.75140100	-2.24094600	L
C-C_3	0	3.98508200	-2.33317000	-1.32494200	L
C-C_3	0	2.61165500	3.40514900	-1.91090000	L
C-C_R	0	-2.01390400	3.87174300	-1.38421300	L
C-C_R	0	-3.59702900	-3.90859600	-0.50089400	L
C-C_R	0	4.05655600	-3.41176300	1.02092400	L
C-C_R	0	4.77469200	2.10600500	-1.22617700	L
C-C_R	0	-2.38704900	4.52727900	-0.13566400	L
C-C_R	0	-4.73741800	-3.06801300	-0.14111900	L
C-C_R	0	3.14400700	-4.25450300	1.78923600	L
C-C_R	0	5.72359400	2.18543400	-0.11995100	L
C-C_R	0	-3.65343200	4.21557700	0.13685800	L
C-C_R	0	-4.90695900	-2.15137800	-1.09429400	L
C-C_R	0	2.22364100	-4.75187200	0.96007900	L
C-C_R	0	5.42520200	3.25460000	0.62075600	L
C-C_R	0	-4.15086000	3.33285400	-0.91488400	L
C-C_R	0	-3.88907600	-2.35595500	-2.12014800	L
C-C_R	0	2.49658100	-4.25359800	-0.38455400	L
C-C_R	0	4.26041900	3.91079200	0.02988100	L
C-C_R	0	-1.65322000	5.31008600	0.68305300	L
C-C_R	0	-5.52055400	-3.10821500	0.95382500	L
C-C_R	0	3.12557200	-4.51376400	3.10897600	L
C-C_R	0	6.75050300	1.37689900	0.19708000	L
C-C_R	0	-2.22567100	5.79778500	1.81413300	L
C-C_R	0	-6.55031100	-2.15019900	1.07737400	L

C-C_R	0	2.09649700	-5.33713900	3.60458200	L
C-C_R	0	7.51539300	1.70259700	1.33901500	L
C-C_R	0	-3.51981000	5.50367700	2.11065300	L
C-C_R	0	-6.72176900	-1.21824900	0.10794100	L
C-C_R	0	1.13316600	-5.84711300	2.70495600	L
C-C_R	0	7.19353600	2.80028600	2.07453300	L
F-F_	0	-0.36361500	5.60720200	0.38289800	L
F-F_	0	-5.32397500	-4.03918900	1.92051600	L
F-F_	0	4.06841000	-4.00472500	3.93942800	L
F-F_	0	7.04529400	0.28512700	-0.55199500	L
F-F_	0	-1.50326600	6.58455500	2.65061800	L
F-F_	0	-7.35788200	-2.15903100	2.16692900	L
F-F_	0	2.02769000	-5.63101500	4.92653400	L
F-F_	0	8.56090300	0.91877500	1.70232100	L
F-F_	0	-4.09226400	5.99584300	3.23747600	L
F-F_	0	-7.70345700	-0.28933000	0.21868300	L
F-F_	0	0.12760200	-6.63477600	3.16106300	L
F-F_	0	7.92362300	3.11320700	3.17428900	L
F-F_	0	-5.56750100	4.39430500	1.49548300	L
F-F_	0	-6.02346700	-0.28560700	-1.99487000	L
F-F_	0	0.28822100	-6.01286300	0.51668300	L
F-F_	0	5.82278900	4.67848800	2.44992300	L
H-H_	0	-2.53336300	-6.55965100	-4.18929400	L
H-H_	0	-2.57260300	3.70151800	-5.84791200	L
H-H_	0	1.82715300	5.01783300	-4.96431500	L
H-H_	0	6.71989800	-4.02834300	-2.75824600	L
H-H_	0	-3.56876900	-5.69973000	-3.00301000	L
H-H_	0	5.77946100	-4.50952800	-1.30994500	L
H-H_	0	-1.30541200	2.88842700	-4.88755400	L
H-H_	0	0.89367300	4.98242900	-3.43168200	L
H-H_	0	-2.03923000	-6.47964800	-2.46073300	L
H-H_	0	6.67264700	-2.94599300	-1.32043900	L
H-H_	0	-2.21428900	4.27315200	-4.18746300	L
H-H_	0	1.14012900	3.47481000	-4.38555200	L
H-H_	0	-2.43185000	-4.48683800	-5.64816300	L
H-H_	0	4.64423500	-4.31087500	-4.21325900	L
H-H_	0	4.09096900	3.94405100	-5.11070900	L
H-H_	0	-5.02503000	3.42712500	-5.31811600	L
H-H_	0	-1.97941100	-2.89752800	-4.97026500	L
H-H_	0	3.09889400	-3.52689800	-3.77384000	L
H-H_	0	3.54453600	2.38089100	-4.44685500	L
H-H_	0	-4.68591500	4.08497000	-3.68848800	L
H-H_	0	-3.52473000	-3.68469600	-4.48641000	L
H-H_	0	3.76981500	-4.84790500	-2.75202600	L

H-H_	0	4.90305600	3.26998900	-3.67033300	L
H-H_	0	-5.53720500	2.50539200	-3.85956800	L
H-H_	0	6.01130400	-2.21084500	-4.22744500	L
H-H_	0	-0.35460600	-5.67354900	-4.89530300	L
H-H_	0	3.73157000	6.11251000	-3.86784900	L
H-H_	0	-3.86493700	1.51121200	-6.22124100	L
H-H_	0	5.91325100	-1.04765400	-2.86525100	L
H-H_	0	0.22012500	-5.52375600	-3.20117800	L
H-H_	0	-4.25965400	0.46400600	-4.81583700	L
H-H_	0	4.52778800	5.41197300	-2.42228400	L
H-H_	0	4.51237000	-1.28105900	-3.97221100	L
H-H_	0	2.87978700	6.12573000	-2.28341400	L
H-H_	0	-2.55829900	0.65577000	-5.37001700	L
H-H_	0	0.28654600	-4.11188800	-4.31572700	L
H-H_	0	-3.87206200	1.23848800	-2.51214200	L
H-H_	0	-1.18752200	-4.40587400	-1.49325000	L
H-H_	0	4.65671200	-1.60473400	-0.81719200	L
H-H_	0	1.95844600	4.09590100	-1.33060900	L
N-N_R	0	-3.11112700	3.08517700	-1.86943900	L
N-N_R	0	-3.04178000	-3.44889000	-1.73900000	L
N-N_R	0	3.63444600	-3.37991400	-0.34769300	L
N-N_R	0	3.83592200	3.18293400	-1.12606800	L
O-O_R	0	-1.54665800	0.78226600	-1.36393800	H
O-O_R	0	-0.65317600	-1.95705300	-1.09652100	H
O-O_R	0	2.12598500	-1.02262500	-0.65554600	H
O-O_R	0	1.25241400	1.71656200	-0.91744200	H
O-O_R	0	-1.00814400	0.87732400	-3.56840700	H
O-O_R	0	-0.43344700	-1.98860400	-3.35900100	H
O-O_R	0	2.36512400	-1.39324700	-2.88650200	H
O-O_R	0	1.77585200	1.46971500	-3.11424600	H
O-O_R	0	-0.83981300	3.79324900	-1.82973200	L
O-O_R	0	-2.96132600	-4.62360400	0.31929500	L
O-O_R	0	4.81677900	-2.54619500	1.53142800	L
O-O_R	0	4.60998000	1.09714200	-1.96052600	L
O-O_R	0	-5.20382800	2.64548400	-0.83481200	L
O-O_R	0	-3.56579200	-1.49054700	-2.97573800	L
O-O_R	0	1.64532800	-4.24294400	-1.31197700	L
O-O_R	0	3.57652200	4.81252500	0.58303500	L
Rh-	0	0.27562200	-0.12154900	-0.89394200	H
Rh-	0	0.68313200	-0.26184200	-3.27007400	H
N-N_3	0	-0.04194700	-0.16956200	1.03838200	H
S-S_3+6	0	-0.65779900	-1.32432800	2.02895000	H
O-O_2	0	-0.08618600	-2.65724900	1.77294000	H
O-O_R	0	-2.27865600	-1.44595900	1.44396100	H

O-O_2	0	-0.71542400	-0.77616300	3.40021500	H
C-C_R	0	-3.27433400	-0.78677300	2.15333400	H
C-C_R	0	-3.73644400	-1.29668000	3.37628800	H
C-C_R	0	-3.87945000	0.34123400	1.57626700	H
C-C_R	0	-4.81512500	-0.67529700	4.01612500	H
H-H_	0	-3.26330500	-2.18184200	3.80495600	H
C-C_R	0	-4.94844300	0.94902600	2.23721800	H
H-H_	0	-3.49969100	0.71825900	0.62461600	H
C-C_R	0	-5.44515400	0.46203800	3.46956100	H
H-H_	0	-5.16534500	-1.09676900	4.95983000	H
H-H_	0	-5.41116200	1.82474000	1.77935300	H
C-C_3	0	-6.60001500	1.19475900	4.17936300	H
C-C_3	0	-6.07942600	2.57337200	4.66961300	H
H-H_	0	-5.70784100	3.18827600	3.83517700	H
H-H_	0	-6.88751000	3.13343700	5.17113900	H
H-H_	0	-5.25120300	2.44903100	5.38635900	H
C-C_3	0	-7.12886700	0.41116300	5.40096500	H
H-H_	0	-7.97492900	0.95536500	5.85212800	H
H-H_	0	-7.48948000	-0.59205500	5.11801400	H
H-H_	0	-6.36074200	0.29546000	6.18328100	H
C-C_3	0	-7.78418900	1.40893700	3.20122300	H
H-H_	0	-8.59915000	1.95230000	3.70889600	H
H-H_	0	-7.49671300	1.99885500	2.31659600	H
H-H_	0	-8.18643200	0.44508200	2.84928800	H
C-C_R	0	3.22753800	0.45861500	3.59003400	H
C-C_R	0	4.61208300	0.33738900	3.35537700	H
C-C_R	0	5.41733500	-0.49808400	4.13997100	H
C-C_R	0	4.84313900	-1.24441300	5.18036700	H
C-C_R	0	3.46049000	-1.15449200	5.41520500	H
C-C_R	0	2.66170200	-0.31465200	4.62959300	H
H-H_	0	5.05393200	0.91000000	2.53822100	H
H-H_	0	6.49027300	-0.56796400	3.93684300	H
H-H_	0	5.46406700	-1.90285800	5.79479200	H
H-H_	0	3.00167500	-1.75055800	6.20965300	H
H-H_	0	1.58350900	-0.27047600	4.80570700	H
C-C_3	0	2.37714200	1.38452100	2.75100900	H
H-H_	0	1.55680500	0.80188200	2.28005200	H
C-C_3	0	1.72851900	2.56799700	3.52089800	H
C-C_3	0	2.79252900	3.52536100	4.08636200	H
H-H_	0	3.35974100	4.00483500	3.26980000	H
H-H_	0	2.32554300	4.32822800	4.68183900	H
H-H_	0	3.51280900	2.99615700	4.73089100	H
C-C_3	0	0.73105500	3.31490300	2.61857700	H
H-H_	0	1.23760900	3.72969300	1.72985400	H

H-H_	0	-0.06740200	2.64461100	2.26157300	H
H-H_	0	0.25938100	4.15385700	3.15844800	H
H-H_	0	1.16373700	2.14156000	4.37124900	H
H-H_	0	2.97942800	1.78715400	1.91622400	H

[3]TS4

C-C_R	0	2.48815700	-5.16466200	1.47719500	L
C-C_R	0	-5.10592300	4.03760200	1.04391400	L
C-C_R	0	4.81785300	4.15795400	2.20163900	L
C-C_R	0	-5.43446300	-2.23147800	-0.95105100	L
C-C_3	0	5.35210700	-1.96490300	-2.64020200	L
C-C_3	0	2.20986500	4.60923600	-3.27989100	L
C-C_3	0	-3.71860700	1.65343900	-4.26397900	L
C-C_3	0	-0.78884400	-4.87520500	-3.50638800	L
C-C_3	0	0.91514000	4.63155900	-4.12683800	L
C-C_3	0	6.73297300	-2.41817700	-2.10759700	L
C-C_3	0	-1.29838200	-6.30525100	-3.20677600	L
C-C_3	0	-2.85788600	2.79376500	-4.85634800	L
C-C_3	0	4.74309900	-3.13873000	-3.44174600	L
C-C_3	0	-1.63439400	-4.30559800	-4.66909100	L
C-C_3	0	3.36348800	4.02619900	-4.12923800	L
C-C_3	0	-5.19504400	2.11953100	-4.27243700	L
C-C_3	0	2.56638800	6.07676200	-2.94272100	L
C-C_3	0	0.68557900	-4.99663800	-3.96045600	L
C-C_3	0	-3.63195500	0.41072100	-5.18329900	L
C-C_3	0	5.60107600	-0.76784500	-3.58922900	L
C-C_R	0	-1.87877800	0.76312700	-2.56818300	H
C-C_R	0	-0.30649200	-2.60318300	-2.21957800	H
C-C_R	0	3.05473500	-0.98649200	-1.73192700	H
C-C_R	0	1.45313900	2.38989600	-2.03414400	H
C-C_3	0	-3.30302800	1.29164700	-2.78699100	L
C-C_3	0	-0.89261700	-4.01772400	-2.18983200	L
C-C_3	0	4.45258300	-1.53561000	-1.42098700	L
C-C_3	0	1.97748700	3.82584700	-1.93347100	L
C-C_R	0	-2.69418100	3.49721000	-1.51457100	L
C-C_R	0	-2.72232200	-4.48129700	-0.42343400	L
C-C_R	0	4.85392200	-2.55776800	0.90340500	L
C-C_R	0	4.29566600	2.95740200	-1.11451900	L
C-C_R	0	-3.23338400	4.18678000	-0.34462600	L
C-C_R	0	-3.98366600	-3.83966100	-0.05747100	L
C-C_R	0	4.19080700	-3.56085700	1.73143700	L
C-C_R	0	5.00790400	3.02597900	0.15744600	L
C-C_R	0	-4.38513600	3.60589500	-0.00581500	L
C-C_R	0	-4.32639800	-2.99285400	-1.02809000	L

C-C_R	0	3.29116800	-4.20020500	0.98015200	L
C-C_R	0	4.39997600	3.91716600	0.94065800	L
C-C_R	0	-4.63883100	2.50975300	-0.93455000	L
C-C_R	0	-3.30768700	-3.04742300	-2.07173000	L
C-C_R	0	3.35896600	-3.65977100	-0.37465700	L
C-C_R	0	3.26838600	4.47817600	0.20706800	L
C-C_R	0	-2.73058300	5.22862600	0.35069200	L
C-C_R	0	-4.72710300	-3.98247300	1.05652000	L
C-C_R	0	4.36076000	-3.84578900	3.03479500	L
C-C_R	0	6.07483800	2.32786000	0.58646500	L
C-C_R	0	-3.41809600	5.69646000	1.42476400	L
C-C_R	0	-5.90222200	-3.20970800	1.18233800	L
C-C_R	0	3.54838400	-4.84739400	3.60052900	L
C-C_R	0	6.53731700	2.57340400	1.89793300	L
C-C_R	0	-4.59688200	5.12432300	1.78842000	L
C-C_R	0	-6.24963200	-2.34863300	0.19483000	L
C-C_R	0	2.59902700	-5.50340000	2.78385300	L
C-C_R	0	5.89512200	3.48223600	2.67896600	L
F-F_	0	-1.55457400	5.79967400	-0.01338600	L
F-F_	0	-4.35566200	-4.83947900	2.04011000	L
F-F_	0	5.27655500	-3.18607200	3.78669400	L
F-F_	0	6.68353000	1.41163900	-0.20728300	L
F-F_	0	-2.92538100	6.73950400	2.13874000	L
F-F_	0	-6.67472900	-3.32456700	2.29119900	L
F-F_	0	3.67090100	-5.17292100	4.91114300	L
F-F_	0	7.60812300	1.89398100	2.37884200	L
F-F_	0	-5.28333900	5.59416600	2.85969400	L
F-F_	0	-7.37415700	-1.59903800	0.30539300	L
F-F_	0	1.80209300	-6.46723600	3.30879000	L
F-F_	0	6.32796800	3.70993900	3.94434300	L
F-F_	0	-6.28942000	3.46179500	1.37083100	L
F-F_	0	-5.76810700	-1.37698200	-1.94990300	L
F-F_	0	1.57947300	-5.78532700	0.68294300	L
F-F_	0	4.17302300	5.05947600	2.98384500	L
H-H_	0	-1.19917600	-6.95738400	-4.10173600	L
H-H_	0	-3.18423000	3.02409100	-5.89361000	L
H-H_	0	1.04754500	5.26727700	-5.02905700	L
H-H_	0	7.41646300	-2.67683600	-2.94543700	L
H-H_	0	-2.37031300	-6.29495100	-2.91460300	L
H-H_	0	6.64002900	-3.31867600	-1.46357800	L
H-H_	0	-1.78400600	2.52894500	-4.90141900	L
H-H_	0	0.06968400	5.04826700	-3.53940500	L
H-H_	0	-0.71723200	-6.76580900	-2.37857600	L
H-H_	0	7.20876100	-1.60991500	-1.51080100	L

H-H_	0	-2.96459300	3.72395700	-4.26111800	L
H-H_	0	0.63238000	3.62015100	-4.47993000	L
H-H_	0	-1.49489800	-4.92127100	-5.58401000	L
H-H_	0	5.39592900	-3.39686500	-4.30353000	L
H-H_	0	3.46687800	4.59409000	-5.07919800	L
H-H_	0	-5.54502600	2.31327200	-5.30974200	L
H-H_	0	-1.35061500	-3.26704000	-4.92594000	L
H-H_	0	3.74213600	-2.89482900	-3.84740500	L
H-H_	0	3.19403700	2.96622900	-4.39866400	L
H-H_	0	-5.32352200	3.06248600	-3.69952200	L
H-H_	0	-2.71621900	-4.32829600	-4.42444200	L
H-H_	0	4.65493400	-4.04757400	-2.81139500	L
H-H_	0	4.33272800	4.10649400	-3.59551100	L
H-H_	0	-5.85434400	1.34458700	-3.82462500	L
H-H_	0	6.33299700	-1.04101800	-4.38007800	L
H-H_	0	0.77337600	-5.68686500	-4.82739600	L
H-H_	0	2.69518200	6.67684100	-3.86975100	L
H-H_	0	-4.03824500	0.64123300	-6.19203700	L
H-H_	0	6.01474500	0.09685200	-3.02771400	L
H-H_	0	1.31630000	-5.40025200	-3.13997500	L
H-H_	0	-4.22507200	-0.42893800	-4.76233600	L
H-H_	0	3.51568600	6.13569300	-2.36839400	L
H-H_	0	4.67865200	-0.44164500	-4.10730900	L
H-H_	0	1.76348600	6.54873800	-2.33581700	L
H-H_	0	-2.59108800	0.06521100	-5.33251800	L
H-H_	0	1.10521200	-4.02147500	-4.27757100	L
H-H_	0	-3.96285100	0.45277300	-2.47708100	L
H-H_	0	-0.27480700	-4.55914000	-1.43799600	L
H-H_	0	4.98231500	-0.68696500	-0.93188400	L
H-H_	0	1.16872000	4.38126900	-1.40647500	L
N-N_R	0	-3.56715200	2.41947300	-1.87759500	L
N-N_R	0	-2.27586100	-3.96572100	-1.68379200	L
N-N_R	0	4.34804000	-2.62252600	-0.43247700	L
N-N_R	0	3.17622800	3.85450200	-1.07856900	L
O-O_R	0	-1.57834500	0.47225100	-1.35213400	H
O-O_R	0	-0.19583500	-2.05728800	-1.06559300	H
O-O_R	0	2.38392000	-0.61338900	-0.70246000	H
O-O_R	0	0.99243300	1.90789300	-0.93497500	H
O-O_R	0	-1.09517700	0.63911300	-3.56346400	H
O-O_R	0	0.00126800	-2.06132500	-3.33338000	H
O-O_R	0	2.64358400	-0.93659200	-2.93668300	H
O-O_R	0	1.52254100	1.77309400	-3.14564800	H
O-O_R	0	-1.50841900	3.60559600	-1.92221500	L
O-O_R	0	-1.95827600	-5.05308500	0.39960900	L

O-O_R	0	5.92285100	-1.96640600	1.21012300	L
O-O_R	0	4.39017300	1.99597500	-1.92229800	L
O-O_R	0	-5.53998800	1.63877300	-0.80580000	L
O-O_R	0	-3.15296900	-2.15987600	-2.95116700	L
O-O_R	0	2.47649900	-3.83491400	-1.25521100	L
O-O_R	0	2.32658200	5.12744400	0.73474300	L
Rh-	0	0.38171500	-0.08554000	-0.90037800	H
Rh-	0	0.77801600	-0.14932900	-3.28661900	H
N-N_3	0	0.17144100	-0.10797600	1.09661000	H
S-S_3+6	0	-0.22082300	-1.40489900	2.06721300	H
O-O_2	0	0.59465700	-2.59064100	1.76105000	H
O-O_R	0	-1.77876000	-1.82256600	1.49203900	H
O-O_2	0	-0.36167200	-0.92291900	3.45736200	H
C-C_R	0	-2.87672300	-1.29864900	2.17720300	H
C-C_R	0	-3.29206600	-1.87289400	3.38549200	H
C-C_R	0	-3.60899200	-0.25399000	1.59488100	H
C-C_R	0	-4.44964100	-1.39505900	4.01376000	H
H-H_	0	-2.71609000	-2.69292200	3.81804900	H
C-C_R	0	-4.75580800	0.21205700	2.24446400	H
H-H_	0	-3.26997000	0.17165300	0.64816900	H
C-C_R	0	-5.20605800	-0.34024100	3.46490300	H
H-H_	0	-4.75780000	-1.86359200	4.95018000	H
H-H_	0	-5.31463900	1.03163100	1.78920900	H
C-C_3	0	-6.45845500	0.23793900	4.15402700	H
C-C_3	0	-6.15381100	1.69369300	4.59900400	H
H-H_	0	-5.87084400	2.33024700	3.74597500	H
H-H_	0	-7.03973300	2.14432900	5.07910200	H
H-H_	0	-5.32203800	1.71731200	5.32230000	H
C-C_3	0	-6.87001000	-0.57491700	5.40093900	H
H-H_	0	-7.78187500	-0.14041700	5.84321000	H
H-H_	0	-7.08973300	-1.62602300	5.14915200	H
H-H_	0	-6.08916800	-0.56167900	6.17932300	H
C-C_3	0	-7.65592800	0.23895000	3.16869300	H
H-H_	0	-8.54456800	0.68151800	3.65053400	H
H-H_	0	-7.44735500	0.82274800	2.25814300	H
H-H_	0	-7.91256100	-0.78749000	2.85990500	H
C-C_R	0	2.43393300	1.07266400	3.38229700	H
C-C_R	0	3.68051600	0.69359200	2.81734000	H
C-C_R	0	4.66512300	0.07379000	3.58822600	H
C-C_R	0	4.42252600	-0.21501200	4.94206700	H
C-C_R	0	3.17952300	0.11234700	5.51018800	H
C-C_R	0	2.19608800	0.74085600	4.74251800	H
H-H_	0	3.85632700	0.89084800	1.75699200	H
H-H_	0	5.62882700	-0.18205700	3.14125900	H

H-H_	0	5.19243700	-0.70289700	5.54618200	H
H-H_	0	2.97715500	-0.12727500	6.55781000	H
H-H_	0	1.23142800	0.97697300	5.19479300	H
C-C_3	0	1.43842300	1.76272200	2.54048900	H
H-H_	0	0.82390200	0.83673200	1.89159600	H
C-C_3	0	0.33410100	2.61415100	3.17271300	H
C-C_3	0	0.93766700	3.83259800	3.91301400	H
H-H_	0	1.45331900	4.50479400	3.20739600	H
H-H_	0	0.13905100	4.41335400	4.40517700	H
H-H_	0	1.66596400	3.52574400	4.67988300	H
C-C_3	0	-0.68980400	3.07882700	2.12194600	H
H-H_	0	-0.20425600	3.68268400	1.33661600	H
H-H_	0	-1.18297200	2.22599400	1.63145900	H
H-H_	0	-1.46918800	3.69762900	2.59632600	H
H-H_	0	-0.20299700	1.99274800	3.91151400	H
H-H_	0	1.90076900	2.25234400	1.66651300	H

[3]INT5

C-C_R	0	-5.76942600	-1.46916600	-0.47223400	L
C-C_R	0	5.46598300	3.64475400	2.13643600	L
C-C_R	0	3.06605300	-4.67125800	3.07319800	L
C-C_R	0	-1.18537600	5.92869700	-0.56500400	L
C-C_3	0	-1.82540900	-4.76535200	-3.36956800	L
C-C_3	0	4.88755800	-3.08405800	-2.54998000	L
C-C_3	0	3.26960400	3.75304700	-3.45646600	L
C-C_3	0	-3.46490200	1.99300600	-4.28744000	L
C-C_3	0	5.36786800	-1.92319500	-3.45433100	L
C-C_3	0	-2.60376800	-6.06175700	-3.03962600	L
C-C_3	0	-4.81789400	2.74424200	-4.26519000	L
C-C_3	0	4.28934800	2.80278100	-4.12765600	L
C-C_3	0	-2.61185300	-4.00587000	-4.46356700	L
C-C_3	0	-2.47019600	2.83572900	-5.12032600	L
C-C_3	0	4.11894000	-4.11588100	-3.40729400	L
C-C_3	0	3.97694900	5.10853200	-3.21345400	L
C-C_3	0	6.15085400	-3.77907400	-1.98751800	L
C-C_3	0	-3.70703000	0.63926800	-4.99674300	L
C-C_3	0	2.09735900	4.02104400	-4.43035900	L
C-C_3	0	-0.44761800	-5.18986600	-3.93259800	L
C-C_R	0	1.91509500	1.90486500	-2.11066700	H
C-C_R	0	-1.70059400	0.95752700	-2.59147900	H
C-C_R	0	-0.82124700	-2.64414600	-2.12424800	H
C-C_R	0	2.77626500	-1.72597300	-1.67714800	H
C-C_3	0	2.74166500	3.19951800	-2.07819500	L
C-C_3	0	-2.98505100	1.76696100	-2.80323900	L

C-C_3	0	-1.65132100	-3.92940500	-2.04566200	L
C-C_3	0	4.03456500	-2.53542800	-1.34577500	L
C-C_R	0	4.64897900	1.90644800	-0.85098700	L
C-C_R	0	-3.70823100	3.45018600	-0.98224000	L
C-C_R	0	-3.40805100	-4.12874000	-0.15570700	L
C-C_R	0	2.54616000	-4.47159000	-0.44723500	L
C-C_R	0	5.32331300	2.05342200	0.43399200	L
C-C_R	0	-3.11933100	4.62337300	-0.34146800	L
C-C_R	0	-4.53704600	-3.31982500	0.29795600	L
C-C_R	0	2.23362800	-4.94019100	0.89869000	L
C-C_R	0	5.00195900	3.24466800	0.93917500	L
C-C_R	0	-1.97132100	4.90878400	-0.95803300	L
C-C_R	0	-4.79356800	-2.38946700	-0.62504100	L
C-C_R	0	3.13662700	-4.44524400	1.74397100	L
C-C_R	0	4.09652200	3.91783700	0.00956800	L
C-C_R	0	-1.78071200	3.95647700	-2.04733900	L
C-C_R	0	-3.84336300	-2.55332500	-1.72147300	L
C-C_R	0	4.08391300	-3.63588300	0.98291900	L
C-C_R	0	6.11977200	1.18881600	1.09720600	L
C-C_R	0	-3.56184700	5.34363800	0.70603100	L
C-C_R	0	-5.23884300	-3.39721200	1.44296100	L
C-C_R	0	1.20584300	-5.69237100	1.33188200	L
C-C_R	0	6.61681200	1.54914600	2.30861300	L
C-C_R	0	-2.77538500	6.42736900	1.15398600	L
C-C_R	0	-6.27641600	-2.46676400	1.64395900	L
C-C_R	0	1.11698100	-5.94941100	2.71748800	L
C-C_R	0	6.31102500	2.76100900	2.84355900	L
C-C_R	0	-1.60676000	6.71483400	0.52927300	L
C-C_R	0	-6.52637600	-1.49309300	0.65041600	L
C-C_R	0	2.05074600	-5.43160600	3.55907000	L
F-F_	0	6.41723400	-0.02409200	0.56679000	L
F-F_	0	-4.72788200	5.03405400	1.32478900	L
F-F_	0	-4.95704300	-4.33720600	2.37924800	L
F-F_	0	0.27844100	-6.18151400	0.47136300	L
F-F_	0	7.41622700	0.69215200	2.99190800	L
F-F_	0	-3.18635200	7.17327500	2.20956700	L
F-F_	0	-7.01756300	-2.49975500	2.77864900	L
F-F_	0	0.09875000	-6.70125100	3.20489800	L
F-F_	0	6.80279100	3.11633600	4.05656100	L
F-F_	0	-0.84128500	7.75001900	0.95578800	L
F-F_	0	-7.51337900	-0.57989600	0.82628700	L
F-F_	0	1.96387000	-5.66927600	4.89194500	L
F-F_	0	5.12716700	4.85087300	2.65605100	L
F-F_	0	-0.01756600	6.19730300	-1.20116700	L

F-F_	0	-5.98978800	-0.52978900	-1.42641700	L
F-F_	0	3.99049300	-4.14368000	3.91462700	L
H-H_	0	-5.21849700	2.87098900	-5.29452300	L
H-H_	0	4.64485000	3.23535600	-5.08789000	L
H-H_	0	6.06824200	-2.29695200	-4.23249100	L
H-H_	0	-2.69985500	-6.70701600	-3.93988800	L
H-H_	0	-4.70744400	3.75718600	-3.82241300	L
H-H_	0	-3.62994100	-5.83330100	-2.68025100	L
H-H_	0	3.85452400	1.81133700	-4.35769800	L
H-H_	0	5.90358600	-1.15573400	-2.85584700	L
H-H_	0	-5.57014000	2.18349400	-3.66898400	L
H-H_	0	-2.08109200	-6.64509800	-2.25073900	L
H-H_	0	5.18010800	2.65498800	-3.48271200	L
H-H_	0	4.53012300	-1.43141600	-3.98604500	L
H-H_	0	-2.83551400	2.94126300	-6.16480900	L
H-H_	0	-2.66961300	-4.61676500	-5.39039200	L
H-H_	0	4.74208700	-4.44253100	-4.26781600	L
H-H_	0	4.30257700	5.56447400	-4.17375500	L
H-H_	0	-1.46496800	2.37528100	-5.16961500	L
H-H_	0	-2.13570100	-3.04355400	-4.73282500	L
H-H_	0	3.17838900	-3.70114300	-3.81883200	L
H-H_	0	4.88205900	4.98248200	-2.58192500	L
H-H_	0	-2.36935300	3.85977900	-4.70622400	L
H-H_	0	-3.65415500	-3.80637700	-4.14074700	L
H-H_	0	3.87658900	-5.02314200	-2.81650700	L
H-H_	0	3.29397200	5.82412600	-2.70629700	L
H-H_	0	-0.57283800	-5.86594200	-4.80608900	L
H-H_	0	-4.12777100	0.79959100	-6.01312100	L
H-H_	0	6.80657500	-4.13734800	-2.81075200	L
H-H_	0	2.46767100	4.48817200	-5.36864600	L
H-H_	0	0.13824900	-5.73623800	-3.16322600	L
H-H_	0	-4.43270400	0.02307600	-4.42483100	L
H-H_	0	1.36276100	4.71749900	-3.97354400	L
H-H_	0	5.88154600	-4.65997700	-1.36630500	L
H-H_	0	0.14921900	-4.32335000	-4.27827500	L
H-H_	0	6.73912700	-3.07665500	-1.35791300	L
H-H_	0	1.56841300	3.09244800	-4.72012300	L
H-H_	0	-2.77307600	0.05736800	-5.12151300	L
H-H_	0	2.04030200	3.97081400	-1.68732100	L
H-H_	0	-3.78311000	1.16386700	-2.31369500	L
H-H_	0	-1.08321400	-4.58695400	-1.35070700	L
H-H_	0	4.69585200	-1.81301400	-0.81787200	L
N-N_R	0	3.84702200	3.06491900	-1.11348700	L
N-N_R	0	-2.88354200	3.04225100	-2.07647500	L

N-N_R	0	-2.95300400	-3.63610100	-1.42085800	L
N-N_R	0	3.69362500	-3.60992200	-0.39584200	L
O-O_R	0	1.36926700	1.57283800	-0.99340600	H
O-O_R	0	-1.42651500	0.68673800	-1.36597600	H
O-O_R	0	-0.60367800	-2.08703800	-0.99128000	H
O-O_R	0	2.18124100	-1.21885900	-0.65937900	H
O-O_R	0	1.83163200	1.24561900	-3.19642400	H
O-O_R	0	-0.98802700	0.62262900	-3.59254000	H
O-O_R	0	-0.41996700	-2.20851000	-3.25530600	H
O-O_R	0	2.39085300	-1.60464000	-2.88667000	H
O-O_R	0	4.58572600	0.83945000	-1.51537600	L
O-O_R	0	-4.90129600	3.07388600	-0.83476700	L
O-O_R	0	-2.70099500	-4.84374200	0.60374800	L
O-O_R	0	1.71745800	-4.46560300	-1.39484900	L
O-O_R	0	3.37655600	4.90891000	0.30484600	L
O-O_R	0	-0.67685100	3.73436100	-2.60954600	L
O-O_R	0	-3.58078000	-1.65480200	-2.56280800	L
O-O_R	0	4.87647700	-2.80155400	1.49580400	L
Rh-	0	0.36493000	-0.27028500	-0.89619800	H
Rh-	0	0.71232100	-0.48624200	-3.27672100	H
N-N_3	0	0.06621000	-0.10476700	1.12022000	H
S-S_3+6	0	-0.41146800	-1.32893400	2.14726900	H
O-O_2	0	-0.40062700	-0.83440900	3.53717000	H
O-O_R	0	-2.04339300	-1.56717600	1.68305000	H
O-O_2	0	0.22124600	-2.59194000	1.76340000	H
C-C_R	0	-3.02183900	-0.77269100	2.28108500	H
C-C_R	0	-3.58978700	0.27724900	1.54809400	H
C-C_R	0	-3.50294500	-1.08850900	3.56182700	H
C-C_R	0	-4.65336600	1.00407200	2.10284800	H
H-H_	0	-3.20280200	0.50357400	0.55215200	H
C-C_R	0	-4.56528900	-0.35208200	4.09351700	H
H-H_	0	-3.05081300	-1.91137900	4.11793600	H
C-C_R	0	-5.17158900	0.70688700	3.38031400	H
H-H_	0	-5.08957900	1.81297200	1.51545200	H
H-H_	0	-4.93123500	-0.61776000	5.08911600	H
C-C_3	0	-6.34049400	1.49110000	4.01013600	H
C-C_3	0	-5.83783600	2.20363500	5.29402100	H
H-H_	0	-5.45401300	1.48538600	6.03627300	H
H-H_	0	-6.65938800	2.76948800	5.76633600	H
H-H_	0	-5.02499500	2.91098500	5.06004800	H
C-C_3	0	-6.91298200	2.56027500	3.05459100	H
H-H_	0	-7.75800300	3.07683100	3.53904300	H
H-H_	0	-7.28835000	2.11563400	2.11772200	H
H-H_	0	-6.16305900	3.32620900	2.79848900	H

C-C_3	0	-7.48875900	0.51626500	4.38112700	H
H-H_	0	-8.31322400	1.06688300	4.86578700	H
H-H_	0	-7.15707000	-0.26898200	5.07871600	H
H-H_	0	-7.89294800	0.02040200	3.48393100	H
C-C_R	0	2.81423500	1.82095400	3.43723300	H
C-C_R	0	3.33717000	2.33887900	4.63817800	H
C-C_R	0	4.15231200	1.55597400	5.46813600	H
C-C_R	0	4.45804600	0.23554500	5.10701700	H
C-C_R	0	3.94508100	-0.29136600	3.91277400	H
C-C_R	0	3.13439900	0.49580200	3.08243700	H
H-H_	0	3.11288000	3.37357200	4.91866600	H
H-H_	0	4.55432800	1.98074900	6.39274300	H
H-H_	0	5.09592200	-0.37782400	5.74980400	H
H-H_	0	4.17698200	-1.31968300	3.62536800	H
H-H_	0	2.75416200	0.07870700	2.14527300	H
C-C_3	0	1.94466500	2.67971800	2.53600600	H
H-H_	0	2.27199400	3.74034600	2.63949800	H
H-H_	0	2.13519300	2.42328100	1.47774400	H
C-C_3	0	0.44696900	2.63750700	2.76603100	H
C-C_3	0	-0.38842900	3.40684700	1.78116500	H
H-H_	0	-0.38517000	4.49123600	2.03077200	H
H-H_	0	-1.44336700	3.08562700	1.79552200	H
H-H_	0	-0.00486500	3.31479100	0.75150700	H
C-C_3	0	-0.11101400	2.40825300	4.13750600	H
H-H_	0	-0.03962300	3.33446600	4.75162200	H
H-H_	0	0.43039300	1.61921700	4.67882900	H
H-H_	0	-1.17744800	2.13501300	4.09774500	H
H-H_	0	0.11764000	0.80308200	1.63709600	H

^{3}TS5

C-C_R	0	-6.18347800	-0.91612900	-0.56288400	L
C-C_R	0	5.96185800	2.95052300	2.02149800	L
C-C_R	0	2.73640500	-5.28475000	2.74067900	L
C-C_R	0	-0.25712800	6.04276200	0.05744100	L
C-C_3	0	-2.42543100	-4.24692100	-3.62409400	L
C-C_3	0	4.31154800	-3.36674400	-2.81914300	L
C-C_3	0	3.58808500	3.60653100	-3.49587000	L
C-C_3	0	-3.38380600	2.84874300	-3.80402600	L
C-C_3	0	4.89450000	-2.21889200	-3.67820500	L
C-C_3	0	-3.30324400	-5.50222600	-3.40375100	L
C-C_3	0	-4.60816300	3.78364500	-3.65414800	L
C-C_3	0	4.42052700	2.53717300	-4.24149400	L
C-C_3	0	-3.13983200	-3.34517500	-4.65794000	L
C-C_3	0	-2.32403500	3.59024200	-4.65246700	L

C-C_3	0	3.37058900	-4.23218600	-3.68969600	L
C-C_3	0	4.49122200	4.84499100	-3.28095300	L
C-C_3	0	5.50033000	-4.25502700	-2.37871200	L
C-C_3	0	-3.86111900	1.59081500	-4.56738800	L
C-C_3	0	2.40502700	4.05226300	-4.38859400	L
C-C_3	0	-1.07694900	-4.72756600	-4.21266700	L
C-C_R	0	2.06324100	1.95165100	-2.08729600	H
C-C_R	0	-1.70388600	1.46126600	-2.27739100	H
C-C_R	0	-1.26294200	-2.32856600	-2.20168200	H
C-C_R	0	2.43978400	-1.81595000	-1.73851700	H
C-C_3	0	3.07254200	3.10669400	-2.09450000	L
C-C_3	0	-2.86150100	2.46398100	-2.36811300	L
C-C_3	0	-2.20194500	-3.53731600	-2.23541800	L
C-C_3	0	3.60024700	-2.79933000	-1.53301600	L
C-C_R	0	4.85314100	1.52205200	-1.03650500	L
C-C_R	0	-3.19157900	4.07443500	-0.38308500	L
C-C_R	0	-3.98879700	-3.72496400	-0.37409800	L
C-C_R	0	1.91387200	-4.57927400	-0.65411500	L
C-C_R	0	5.57173900	1.50208800	0.23274000	L
C-C_R	0	-2.36529000	5.05301800	0.31853200	L
C-C_R	0	-5.07308400	-2.87715600	0.11318900	L
C-C_R	0	1.66300600	-5.15881500	0.66074200	L
C-C_R	0	5.41964000	2.68959800	0.81858300	L
C-C_R	0	-1.23553400	5.22242400	-0.36947300	L
C-C_R	0	-5.25555100	-1.87782900	-0.75389600	L
C-C_R	0	2.70701400	-4.89853000	1.44726200	L
C-C_R	0	4.58264400	3.52970400	-0.03566700	L
C-C_R	0	-1.29123000	4.38571300	-1.56371400	L
C-C_R	0	-4.29053700	-2.02453300	-1.84055800	L
C-C_R	0	3.68325200	-4.13348000	0.67528400	L
C-C_R	0	6.26447100	0.50188400	0.81767800	L
C-C_R	0	-2.59492300	5.69579200	1.47887700	L
C-C_R	0	-5.80032300	-2.98269600	1.23974100	L
C-C_R	0	0.58251600	-5.81331900	1.12240400	L
C-C_R	0	6.83625700	0.71886200	2.03016100	L
C-C_R	0	-1.60083200	6.57164700	1.96714200	L
C-C_R	0	-6.78912300	-2.00956900	1.47889500	L
C-C_R	0	0.59295700	-6.22991000	2.47150300	L
C-C_R	0	6.70633300	1.92453100	2.64459500	L
C-C_R	0	-0.45126800	6.74198000	1.26843500	L
C-C_R	0	-6.96695600	-0.96764200	0.54048100	L
C-C_R	0	1.67232600	-5.95684800	3.25177500	L
F-F_	0	6.38814500	-0.70352200	0.20714400	L
F-F_	0	-3.74272300	5.49650800	2.17378300	L

F-F_	0	-5.59245600	-3.99355900	2.11985800	L
F-F_	0	-0.48633500	-6.05995800	0.32445200	L
F-F_	0	7.53532000	-0.27466200	2.63407500	L
F-F_	0	-1.79372400	7.23318100	3.13535800	L
F-F_	0	-7.55766100	-2.07208100	2.59395300	L
F-F_	0	-0.47214100	-6.89395000	2.98556900	L
F-F_	0	7.27414800	2.13816600	3.85767900	L
F-F_	0	0.51267600	7.57553100	1.73267900	L
F-F_	0	-7.91461000	-0.02031200	0.74767800	L
F-F_	0	1.68429900	-6.35252200	4.54943800	L
F-F_	0	5.79442100	4.15433900	2.62347700	L
F-F_	0	0.88921500	6.19325700	-0.65230900	L
F-F_	0	-6.33160000	0.08990700	-1.46147300	L
F-F_	0	3.81048600	-5.00784200	3.52166500	L
H-H_	0	-5.04104400	4.03302000	-4.64738300	L
H-H_	0	4.75809300	2.92909500	-5.22536300	L
H-H_	0	5.48253000	-2.62679800	-4.52884400	L
H-H_	0	-3.44203600	-6.06127900	-4.35468000	L
H-H_	0	-4.32573300	4.73909300	-3.16246100	L
H-H_	0	-4.31109300	-5.22533800	-3.02692100	L
H-H_	0	3.84179100	1.61418400	-4.43745000	L
H-H_	0	5.57385400	-1.58294400	-3.07175900	L
H-H_	0	-5.40134100	3.29955800	-3.04393200	L
H-H_	0	-2.83274500	-6.18773900	-2.66585500	L
H-H_	0	5.32942500	2.26675000	-3.66525900	L
H-H_	0	4.10531700	-1.57520200	-4.11281200	L
H-H_	0	-2.72689100	3.81096400	-5.66466300	L
H-H_	0	-3.22818500	-3.87253900	-5.63243600	L
H-H_	0	3.90607900	-4.59269300	-4.59456500	L
H-H_	0	4.82962800	5.26331300	-4.25386300	L
H-H_	0	-1.40128400	2.99463900	-4.78959700	L
H-H_	0	-2.59221200	-2.40142400	-4.84344000	L
H-H_	0	2.48193100	-3.67011600	-4.03581800	L
H-H_	0	5.39885800	4.58339600	-2.69616800	L
H-H_	0	-2.05246900	4.56210200	-4.19180100	L
H-H_	0	-4.16992800	-3.09796100	-4.32926600	L
H-H_	0	3.02611800	-5.12860700	-3.13410300	L
H-H_	0	3.94261100	5.64271800	-2.73463500	L
H-H_	0	-1.24237100	-5.32074900	-5.13813800	L
H-H_	0	-4.30807500	1.87230200	-5.54552100	L
H-H_	0	6.06436300	-4.62950800	-3.26059200	L
H-H_	0	2.77643000	4.50776000	-5.33214600	L
H-H_	0	-0.54081900	-5.37518400	-3.48676400	L
H-H_	0	-4.63788100	1.05263500	-3.98390400	L

```
H-H_      0   1.78486900   4.81120200  -3.86553900 L
H-H_      0   5.15017300  -5.14015500  -1.80576500 L
H-H_      0  -0.41431400  -3.88251800  -4.48421700 L
H-H_      0   6.20384000  -3.68135500  -1.73697200 L
H-H_      0   1.75285000   3.20442100  -4.67564500 L
H-H_      0  -3.03085900   0.88931900  -4.78022800 L
H-H_      0   2.51768000   3.96446200  -1.65224300 L
H-H_      0  -3.71356200   1.95395600  -1.86237000 L
H-H_      0  -1.70362000  -4.29660300  -1.59244200 L
H-H_      0   4.37451200  -2.20616600  -0.99756600 L
N-N_R     0   4.19886300   2.78780900  -1.19950900 L
N-N_R     0  -2.53017300   3.66427800  -1.58292300 L
N-N_R     0  -3.48034200  -3.18207400  -1.59702400 L
N-N_R     0   3.17278700  -3.89164500  -0.64095400 L
O-O_R     0   1.53497500   1.67972500  -0.95750200 H
O-O_R     0  -1.42263600   1.07470600  -1.08315400 H
O-O_R     0  -0.97086400  -1.89837600  -1.04171900 H
O-O_R     0   1.96974900  -1.30169300  -0.66167600 H
O-O_R     0   1.81748000   1.31818900  -3.16824600 H
O-O_R     0  -1.09653700   1.10108500  -3.33622700 H
O-O_R     0  -0.84129600  -1.81196500  -3.29613200 H
O-O_R     0   2.01514300  -1.58314800  -2.92080900 H
O-O_R     0   4.57721400   0.49900700  -1.71550100 L
O-O_R     0  -4.42285300   3.90388600  -0.18046900 L
O-O_R     0  -3.34429500  -4.53343600   0.34619700 L
O-O_R     0   1.02385700  -4.38627300  -1.52261600 L
O-O_R     0   3.97888100   4.56305500   0.35882800 L
O-O_R     0  -0.28746500   4.06763100  -2.25369600 L
O-O_R     0  -3.93021700  -1.07602100  -2.58600000 L
O-O_R     0   4.65295900  -3.50190300   1.17302500 L
Rh-       0   0.25236000  -0.13582800  -0.77330700 H
Rh-       0   0.47756700  -0.24301700  -3.19651700 H
N-N_3     0  -0.09144300   0.05028600   1.35920500 H
S-S_3+6   0  -0.41485800  -1.35218700   2.25259900 H
O-O_2     0  -0.44184900  -1.06081500   3.70122800 H
O-O_R     0  -2.03190100  -1.74907900   1.80638300 H
O-O_2     0   0.38495200  -2.46097900   1.73135700 H
C-C_R     0  -3.02121200  -0.88763200   2.28707400 H
C-C_R     0  -3.52626400   0.11089800   1.44264500 H
C-C_R     0  -3.54383900  -1.04777400   3.58057900 H
C-C_R     0  -4.53769400   0.96486800   1.90888800 H
H-H_      0  -3.13732100   0.20456400   0.42651000 H
C-C_R     0  -4.55182000  -0.18555000   4.02379400 H
H-H_      0  -3.14786400  -1.83459200   4.22450900 H
```

C-C_R	0	-5.06577300	0.85010400	3.20908900	H
H-H_	0	-4.92466400	1.72263000	1.22677900	H
H-H_	0	-4.93913300	-0.32224700	5.03749700	H
C-C_3	0	-6.13285300	1.81628600	3.76318300	H
C-C_3	0	-5.52563500	2.59545000	4.96088700	H
H-H_	0	-5.20838000	1.91845500	5.77032900	H
H-H_	0	-6.26778700	3.29699600	5.37958100	H
H-H_	0	-4.64423000	3.17843500	4.64520600	H
C-C_3	0	-6.59823000	2.84448300	2.70898300	H
H-H_	0	-7.36690800	3.50274300	3.14651900	H
H-H_	0	-7.04273400	2.35779300	1.82524100	H
H-H_	0	-5.76970100	3.48734300	2.36833700	H
C-C_3	0	-7.37398700	1.02283300	4.24672100	H
H-H_	0	-8.11754800	1.70941200	4.68664300	H
H-H_	0	-7.11495900	0.27624800	5.01412500	H
H-H_	0	-7.85621400	0.49200200	3.41075900	H
C-C_R	0	3.20117300	0.87723200	3.21258800	H
C-C_R	0	3.84223500	1.40842800	4.34922400	H
C-C_R	0	4.61379100	0.59311200	5.18844000	H
C-C_R	0	4.75260500	-0.77223800	4.89949800	H
C-C_R	0	4.11816200	-1.31019800	3.77054100	H
C-C_R	0	3.35083800	-0.49331400	2.92711000	H
H-H_	0	3.75147200	2.47798700	4.56928900	H
H-H_	0	5.11207100	1.02607200	6.06069900	H
H-H_	0	5.35475100	-1.41359900	5.54926600	H
H-H_	0	4.21812500	-2.37408200	3.54672600	H
H-H_	0	2.86492500	-0.92272900	2.04685300	H
C-C_3	0	2.37983200	1.77570300	2.31032000	H
H-H_	0	2.83642300	2.79121500	2.30389900	H
H-H_	0	2.42470000	1.45197600	1.25524300	H
C-C_3	0	0.93652300	2.00235300	2.66601600	H
C-C_3	0	0.20097800	2.97874300	1.80572600	H
H-H_	0	0.50153100	4.00397500	2.11039200	H
H-H_	0	-0.89063300	2.92115400	1.93891200	H
H-H_	0	0.46191200	2.86828000	0.74222800	H
C-C_3	0	0.44382300	1.75989900	4.04760100	H
H-H_	0	0.82861200	2.57603100	4.69874400	H
H-H_	0	0.81297000	0.81435800	4.46478400	H
H-H_	0	-0.65369200	1.78274800	4.10935000	H
H-H_	0	-0.94203500	0.63403300	1.44543300	H

^{3}INT6

C-C_R	0	2.62758400	-5.09163100	1.79788600	L
C-C_R	0	-5.23838400	3.94444700	0.58331400	L

C-C_R	0	4.53631400	4.57496700	1.85455600	L
C-C_R	0	-5.30973400	-2.49374000	-0.61104900	L
C-C_3	0	5.42023100	-2.08529900	-2.51888900	L
C-C_3	0	2.11552800	4.25434000	-3.70347500	L
C-C_3	0	-3.77329400	1.02749500	-4.41805600	L
C-C_3	0	-0.68876100	-5.27034700	-3.07330800	L
C-C_3	0	0.85089700	4.15422800	-4.58991400	L
C-C_3	0	6.81843700	-2.44130900	-1.95851400	L
C-C_3	0	-1.16507800	-6.67881300	-2.64456900	L
C-C_3	0	-2.95840600	2.12873900	-5.13624900	L
C-C_3	0	4.86520300	-3.33495400	-3.24090000	L
C-C_3	0	-1.56874200	-4.81357900	-4.25983600	L
C-C_3	0	3.31206800	3.61333600	-4.44491900	L
C-C_3	0	-5.26596300	1.43600500	-4.46298500	L
C-C_3	0	2.42701000	5.75854700	-3.51511100	L
C-C_3	0	0.77767600	-5.40512600	-3.54911300	L
C-C_3	0	-3.64451600	-0.30163800	-5.20159900	L
C-C_3	0	5.61760400	-0.94324700	-3.54486600	L
C-C_R	0	-1.89725700	0.38535900	-2.65156900	H
C-C_R	0	-0.23468700	-2.88614300	-1.99231600	H
C-C_R	0	3.08393700	-1.14281700	-1.67590300	H
C-C_R	0	1.38405400	2.15398700	-2.25279900	H
C-C_3	0	-3.33901900	0.83758800	-2.91437300	L
C-C_3	0	-0.78008800	-4.30797000	-1.83035000	L
C-C_3	0	4.50056900	-1.61662400	-1.32986000	L
C-C_3	0	1.85630500	3.61203800	-2.28897800	L
C-C_R	0	-2.82534500	3.20687900	-1.92578500	L
C-C_R	0	-2.53428600	-4.63073500	0.03826100	L
C-C_R	0	4.91642200	-2.45730400	1.05990500	L
C-C_R	0	4.14923000	2.90063000	-1.26845200	L
C-C_R	0	-3.39367500	4.01471700	-0.84841800	L
C-C_R	0	-3.79094800	-3.97143300	0.38791700	L
C-C_R	0	4.27048800	-3.41470300	1.95259100	L
C-C_R	0	4.80993100	3.15725400	0.00739000	L
C-C_R	0	-4.50438600	3.41823100	-0.41261700	L
C-C_R	0	-4.19119400	-3.24239900	-0.65330200	L
C-C_R	0	3.40995700	-4.14424500	1.23885100	L
C-C_R	0	4.16963900	4.14885800	0.62713000	L
C-C_R	0	-4.70326900	2.19488200	-1.18178400	L
C-C_R	0	-3.21616700	-3.39421000	-1.72857900	L
C-C_R	0	3.48458500	-3.70869600	-0.15279500	L
C-C_R	0	3.06454400	4.58451300	-0.22285400	L
C-C_R	0	-2.94928500	5.17005400	-0.31044300	L
C-C_R	0	-4.48214300	-3.99953700	1.54371000	L

C-C_R	0	4.41650400	-3.58214100	3.27897100	L
C-C_R	0	5.85693200	2.53435700	0.57766900	L
C-C_R	0	-3.65313700	5.73825900	0.70295700	L
C-C_R	0	-5.66517900	-3.23404200	1.63658400	L
C-C_R	0	3.62602100	-4.56274400	3.90902300	L
C-C_R	0	6.26472800	2.97122700	1.85727800	L
C-C_R	0	-4.78952200	5.15087100	1.16408800	L
C-C_R	0	-6.07203800	-2.49415000	0.57632500	L
C-C_R	0	2.71972900	-5.31806200	3.13004700	L
C-C_R	0	5.59227100	3.98194800	2.46947200	L
F-F_	0	-1.81529400	5.75597700	-0.77139500	L
F-F_	0	-4.05145500	-4.73566500	2.59845800	L
F-F_	0	5.28218400	-2.82076900	3.99381500	L
F-F_	0	6.49560500	1.51359100	-0.04692700	L
F-F_	0	-3.21817100	6.89644900	1.25963800	L
F-F_	0	-6.38603800	-3.23449000	2.78548600	L
F-F_	0	3.72592800	-4.77218000	5.24498800	L
F-F_	0	7.31166000	2.37242600	2.47762700	L
F-F_	0	-5.49138300	5.71970400	2.17571200	L
F-F_	0	-7.20551400	-1.75400700	0.65345300	L
F-F_	0	1.94293700	-6.26243200	3.71688100	L
F-F_	0	5.97148900	4.39345700	3.70526800	L
F-F_	0	-6.37968100	3.34783900	1.00828400	L
F-F_	0	-5.70362900	-1.76198500	-1.68269700	L
F-F_	0	1.75716300	-5.80549100	1.03998200	L
F-F_	0	3.85904300	5.57634100	2.46994600	L
H-H_	0	-1.07243200	-7.40158100	-3.48426900	L
H-H_	0	-3.28718000	2.22333300	-6.19380800	L
H-H_	0	0.99895400	4.70022100	-5.54680300	L
H-H_	0	7.51384800	-2.72640300	-2.77775000	L
H-H_	0	-2.23066700	-6.66250600	-2.33027400	L
H-H_	0	6.76314300	-3.29908600	-1.25461600	L
H-H_	0	-1.87349600	1.90898100	-5.14776700	L
H-H_	0	-0.02483700	4.60304800	-4.07485400	L
H-H_	0	-0.55819900	-7.05851000	-1.79409200	L
H-H_	0	7.25792200	-1.57469400	-1.41849000	L
H-H_	0	-3.11078000	3.11624000	-4.65439400	L
H-H_	0	0.60499300	3.10600900	-4.85036100	L
H-H_	0	-1.43140200	-5.49626200	-5.12624000	L
H-H_	0	5.52977700	-3.61926400	-4.08529000	L
H-H_	0	3.44586100	4.08725100	-5.44145200	L
H-H_	0	-5.62699200	1.50835600	-5.51200900	L
H-H_	0	-1.31472000	-3.79212300	-4.60247300	L
H-H_	0	3.85465500	-3.16304400	-3.65953100	L

H-H_	0	3.17049800	2.52886000	-4.61470600	L
H-H_	0	-5.42687100	2.42773000	-3.98913900	L
H-H_	0	-2.64505000	-4.84179300	-3.99261300	L
H-H_	0	4.81735700	-4.20373000	-2.55234500	L
H-H_	0	4.25647500	3.76258900	-3.88204700	L
H-H_	0	-5.89428700	0.68825600	-3.93207800	L
H-H_	0	6.36509300	-1.23353100	-4.31476900	L
H-H_	0	0.85939600	-6.16341300	-4.35784200	L
H-H_	0	2.56775300	6.26076300	-4.49695800	L
H-H_	0	-4.06148100	-0.19375400	-6.22642500	L
H-H_	0	5.98731900	-0.02451700	-3.04141700	L
H-H_	0	1.43275800	-5.72955500	-2.71275800	L
H-H_	0	-4.20606900	-1.11265900	-4.69088200	L
H-H_	0	3.35830100	5.90422800	-2.92704800	L
H-H_	0	4.68316700	-0.69590300	-4.08475300	L
H-H_	0	1.59590300	6.26988500	-2.98263200	L
H-H_	0	-2.59209900	-0.62366200	-5.31952200	L
H-H_	0	1.17327600	-4.45330500	-3.95524700	L
H-H_	0	-3.96810400	0.01342100	-2.51470500	L
H-H_	0	-0.12714500	-4.76849600	-1.05483900	L
H-H_	0	4.99546500	-0.71847300	-0.89574800	L
H-H_	0	1.01065500	4.18925900	-1.85114500	L
N-N_R	0	-3.63786100	2.04265600	-2.12352100	L
N-N_R	0	-2.14983500	-4.24794000	-1.28855000	L
N-N_R	0	4.43588400	-2.64168200	-0.27423600	L
N-N_R	0	3.02303500	3.77885400	-1.40558000	L
O-O_R	0	-1.58270800	0.22502800	-1.41605700	H
O-O_R	0	-0.14596000	-2.23296400	-0.89343600	H
O-O_R	0	2.39742900	-0.71579100	-0.67915000	H
O-O_R	0	0.94297900	1.75591000	-1.11173300	H
O-O_R	0	-1.10892000	0.19090700	-3.63261300	H
O-O_R	0	0.06442100	-2.44006100	-3.15082600	H
O-O_R	0	2.66823700	-1.19918700	-2.87975400	H
O-O_R	0	1.47032300	1.44321800	-3.30571400	H
O-O_R	0	-1.64372800	3.31323700	-2.34650600	L
O-O_R	0	-1.72574300	-5.09764000	0.88470100	L
O-O_R	0	5.97329600	-1.82777100	1.33099300	L
O-O_R	0	4.30166600	1.85037100	-1.94614600	L
O-O_R	0	-5.53828100	1.29146100	-0.91010800	L
O-O_R	0	-3.10986000	-2.59518400	-2.69565700	L
O-O_R	0	2.61873700	-3.97535800	-1.02694900	L
O-O_R	0	2.10578200	5.30452600	0.16306200	L
Rh-	0	0.38949100	-0.24828100	-0.92172900	H
Rh-	0	0.78394100	-0.50615300	-3.28655000	H

N-N_3	0	0.14401200	-0.04841300	1.08829600	H
S-S_3+6	0	-0.03847600	-1.26272000	2.22638900	H
O-O_2	0	0.83621500	-2.40033400	1.92746500	H
O-O_R	0	-1.57944400	-1.85461100	1.83202300	H
O-O_2	0	-0.09482400	-0.64476000	3.56387600	H
C-C_R	0	-2.67770000	-1.22157900	2.42827200	H
C-C_R	0	-3.09602100	-1.61826000	3.70321500	H
C-C_R	0	-3.39690600	-0.25970300	1.70541800	H
C-C_R	0	-4.24671100	-1.04379400	4.26055100	H
H-H_	0	-2.52566600	-2.37536300	4.24449100	H
C-C_R	0	-4.53516400	0.30852200	2.28717700	H
H-H_	0	-3.06059600	0.02555800	0.70625800	H
C-C_R	0	-4.98992000	-0.06479100	3.57224400	H
H-H_	0	-4.55734900	-1.37334500	5.25361700	H
H-H_	0	-5.08235600	1.06722400	1.72411500	H
C-C_3	0	-6.23534700	0.61686300	4.17388600	H
C-C_3	0	-5.92523400	2.12239400	4.39011000	H
H-H_	0	-5.63857900	2.62026500	3.45064700	H
H-H_	0	-6.80946100	2.64483400	4.79447400	H
H-H_	0	-5.09387700	2.25299600	5.10241800	H
C-C_3	0	-6.64025000	0.00620500	5.53336800	H
H-H_	0	-7.54497400	0.51022200	5.91193300	H
H-H_	0	-6.86981100	-1.06908800	5.44655500	H
H-H_	0	-5.85164500	0.13133000	6.29379200	H
C-C_3	0	-7.43854100	0.46783500	3.20695500	H
H-H_	0	-8.32295000	0.98583400	3.61585900	H
H-H_	0	-7.23175700	0.89670200	2.21369000	H
H-H_	0	-7.70049100	-0.59372800	3.06741000	H
C-C_R	0	2.28875000	2.06844500	3.34450000	H
C-C_R	0	3.29659800	1.28763000	2.69053300	H
C-C_R	0	4.28069400	0.62355600	3.41704200	H
C-C_R	0	4.30768100	0.70182100	4.82221900	H
C-C_R	0	3.32103500	1.45032400	5.49111500	H
C-C_R	0	2.32854800	2.11684500	4.77520300	H
H-H_	0	3.27656400	1.20819900	1.59977200	H
H-H_	0	5.04199200	0.04357500	2.89157400	H
H-H_	0	5.08356400	0.17958500	5.38820300	H
H-H_	0	3.32951100	1.50929800	6.58348800	H
H-H_	0	1.57115300	2.68948700	5.31541700	H
C-C_R	0	1.30999200	2.75730700	2.57291500	H
C-C_3	0	0.18211900	3.59371300	3.11879700	H
C-C_3	0	0.65414000	5.03264400	3.46274200	H
H-H_	0	0.96887100	5.57318400	2.55541300	H
H-H_	0	-0.16697200	5.60511700	3.92774600	H

H-H_	0	1.50627700	5.02008100	4.16046600	H
C-C_3	0	-1.00205900	3.63528500	2.13295500	H
H-H_	0	-0.68204400	4.04680100	1.16024200	H
H-H_	0	-1.41876100	2.63137100	1.95459100	H
H-H_	0	-1.81258400	4.27300800	2.52248500	H
H-H_	0	-0.17581800	3.13279800	4.05857000	H
H-H_	0	1.44854800	2.76518700	1.48372700	H
H-H_	0	0.40884800	0.83432800	1.56443800	H

^{3}TS6

C-C_R	0	2.94708200	-4.76636500	1.69378300	L
C-C_R	0	-5.31585100	3.81075400	1.05465800	L
C-C_R	0	4.50663600	4.29460200	2.25862600	L
C-C_R	0	-5.39452900	-2.50335700	-1.01845900	L
C-C_3	0	5.39128200	-1.82473000	-2.93313200	L
C-C_3	0	2.02754400	4.65330200	-3.31454600	L
C-C_3	0	-3.81744000	1.46547300	-4.24950400	L
C-C_3	0	-0.53134600	-4.96603800	-3.30443200	L
C-C_3	0	0.73909500	4.61681300	-4.17098700	L
C-C_3	0	6.83608400	-2.23058700	-2.55405200	L
C-C_3	0	-0.96825100	-6.41308500	-2.97391300	L
C-C_3	0	-2.99262700	2.63575800	-4.83423600	L
C-C_3	0	4.72234900	-3.03677400	-3.62178500	L
C-C_3	0	-1.38086500	-4.47874300	-4.50150900	L
C-C_3	0	3.20467400	4.08557700	-4.14109700	L
C-C_3	0	-5.31007900	1.87464900	-4.27961700	L
C-C_3	0	2.33689600	6.13807800	-3.00802600	L
C-C_3	0	0.95642800	-5.01643000	-3.72537900	L
C-C_3	0	-3.66943200	0.22387600	-5.16251800	L
C-C_3	0	5.49559600	-0.65066800	-3.93703200	L
C-C_R	0	-1.97424500	0.64353100	-2.52115100	H
C-C_R	0	-0.17152300	-2.64733700	-2.06258800	H
C-C_R	0	3.19427300	-0.85888400	-1.79727000	H
C-C_R	0	1.33200300	2.44037600	-2.02113600	H
C-C_3	0	-3.41078900	1.12240900	-2.76613900	L
C-C_3	0	-0.70973800	-4.08005600	-2.01444700	L
C-C_3	0	4.62796700	-1.38200600	-1.62971700	L
C-C_3	0	1.80740700	3.89440000	-1.95282100	L
C-C_R	0	-2.90019800	3.36390500	-1.51687600	L
C-C_R	0	-2.58646500	-4.57736100	-0.30499800	L
C-C_R	0	5.35366400	-2.36166100	0.63018700	L
C-C_R	0	4.13832300	3.11537100	-1.08544000	L
C-C_R	0	-3.46253900	4.03741200	-0.34868800	L
C-C_R	0	-3.89805000	-4.00109400	-0.01471100	L

C-C_R	0	4.76118100	-3.27164000	1.60602200	L
C-C_R	0	4.80068300	3.18838700	0.21298100	L
C-C_R	0	-4.57187000	3.39323100	0.01585300	L
C-C_R	0	-4.23923800	-3.19493800	-1.02022500	L
C-C_R	0	3.72764400	-3.88804200	1.02978800	L
C-C_R	0	4.14172000	4.05563700	0.98082800	L
C-C_R	0	-4.75649400	2.25223600	-0.87392400	L
C-C_R	0	-3.16789200	-3.21081700	-2.01098000	L
C-C_R	0	3.63420900	-3.42314200	-0.35087300	L
C-C_R	0	3.02430400	4.59536000	0.21074600	L
C-C_R	0	-3.02417800	5.12930600	0.31252300	L
C-C_R	0	-4.68954000	-4.16837400	1.06205600	L
C-C_R	0	5.09867600	-3.49543200	2.88848400	L
C-C_R	0	5.86779100	2.51235800	0.67560900	L
C-C_R	0	-3.73482900	5.58326200	1.37751500	L
C-C_R	0	-5.91484900	-3.46830000	1.10889600	L
C-C_R	0	4.31651600	-4.40608400	3.62481000	L
C-C_R	0	6.27479900	2.75595900	2.00565700	L
C-C_R	0	-4.87394300	4.94906500	1.76416900	L
C-C_R	0	-6.26166600	-2.65057200	0.08478300	L
C-C_R	0	3.22258500	-5.03948400	2.99152700	L
C-C_R	0	5.58122600	3.64034500	2.77028800	L
F-F_	0	-1.89118900	5.76570400	-0.07850900	L
F-F_	0	-4.32113900	-4.98460000	2.08084300	L
F-F_	0	6.15036300	-2.85904000	3.46170000	L
F-F_	0	6.52896600	1.62000200	-0.10296200	L
F-F_	0	-3.30681500	6.67643200	2.05740100	L
F-F_	0	-6.73807300	-3.61211100	2.17710000	L
F-F_	0	4.60445400	-4.66576400	4.92414100	L
F-F_	0	7.34307200	2.09767900	2.52046500	L
F-F_	0	-5.58456300	5.40587700	2.82526000	L
F-F_	0	-7.43728800	-1.97576800	0.11570700	L
F-F_	0	2.45181300	-5.91556400	3.68289100	L
F-F_	0	5.96087900	3.86626000	4.05293900	L
F-F_	0	-6.45654700	3.16790000	1.40700300	L
F-F_	0	-5.72750500	-1.69267400	-2.05340800	L
F-F_	0	1.89794800	-5.36480100	1.07494500	L
F-F_	0	3.81363200	5.17513100	3.02314100	L
H-H_	0	-0.81808500	-7.08245300	-3.84882900	L
H-H_	0	-3.29942900	2.83697400	-5.88344500	L
H-H_	0	0.85328100	5.24821400	-5.07870900	L
H-H_	0	7.42413000	-2.49833000	-3.45876600	L
H-H_	0	-2.04414000	-6.45232300	-2.69919200	L
H-H_	0	6.84231100	-3.11423900	-1.88079900	L

H-H_	0	-1.90691500	2.41995600	-4.84445700	L
H-H_	0	-0.12703600	5.00448300	-3.59345500	L
H-H_	0	-0.37807100	-6.81977800	-2.12421500	L
H-H_	0	7.35698400	-1.39429900	-2.03931500	L
H-H_	0	-3.15928900	3.56842300	-4.25704400	L
H-H_	0	0.49912400	3.59191900	-4.51671900	L
H-H_	0	-1.18852500	-5.11368700	-5.39340600	L
H-H_	0	5.29340900	-3.32818800	-4.52975000	L
H-H_	0	3.29458200	4.62936100	-5.10639900	L
H-H_	0	-5.64914500	2.06492700	-5.32117500	L
H-H_	0	-1.14737600	-3.43531500	-4.78686200	L
H-H_	0	3.68573700	-2.81639100	-3.94185200	L
H-H_	0	3.07185300	3.01296000	-4.37993100	L
H-H_	0	-5.48431500	2.80578500	-3.69933700	L
H-H_	0	-2.46509500	-4.55353600	-4.27912700	L
H-H_	0	4.70028000	-3.91590300	-2.94458300	L
H-H_	0	4.16733200	4.21369500	-3.60468500	L
H-H_	0	-5.94666100	1.07044100	-3.85100200	L
H-H_	0	6.16458000	-0.91733700	-4.78383200	L
H-H_	0	1.10034100	-5.71740700	-4.57602100	L
H-H_	0	2.45881100	6.72011400	-3.94740000	L
H-H_	0	-4.08183800	0.43020700	-6.17401000	L
H-H_	0	5.91308300	0.25224800	-3.44160200	L
H-H_	0	1.58991300	-5.36929200	-2.88404200	L
H-H_	0	-4.22333900	-0.64130400	-4.73938100	L
H-H_	0	3.27725700	6.23830800	-2.42474800	L
H-H_	0	4.51599900	-0.38571200	-4.37963500	L
H-H_	0	1.51342600	6.59979400	-2.42112300	L
H-H_	0	-2.61248500	-0.07212800	-5.30590400	L
H-H_	0	1.32722800	-4.02475700	-4.05258400	L
H-H_	0	-4.04698400	0.26282100	-2.46229900	L
H-H_	0	-0.09835500	-4.58145600	-1.22991800	L
H-H_	0	5.19263800	-0.51441600	-1.22012600	L
H-H_	0	0.97330900	4.43369500	-1.44902300	L
N-N_R	0	-3.72182300	2.24002600	-1.86048700	L
N-N_R	0	-2.10900000	-4.06391600	-1.55421900	L
N-N_R	0	4.65635500	-2.45143200	-0.61565300	L
N-N_R	0	2.99381500	3.98264300	-1.08377000	L
O-O_R	0	-1.67722100	0.37010400	-1.31087700	H
O-O_R	0	-0.02557200	-2.09438000	-0.91965800	H
O-O_R	0	2.61853900	-0.45845600	-0.73413400	H
O-O_R	0	0.90816800	1.96208700	-0.90725700	H
O-O_R	0	-1.16701100	0.54627200	-3.50643500	H
O-O_R	0	0.06066500	-2.09705700	-3.19522200	H

O-O_R	0	2.65145700	-0.86199400	-2.95666800	H
O-O_R	0	1.40567500	1.80480600	-3.12247100	H
O-O_R	0	-1.72538500	3.52848900	-1.93860500	L
O-O_R	0	-1.83493000	-5.09639600	0.56318600	L
O-O_R	0	6.45416700	-1.76483400	0.77169500	L
O-O_R	0	4.29096800	2.16596200	-1.89905100	L
O-O_R	0	-5.79113100	1.53567600	-0.93301500	L
O-O_R	0	-3.02083200	-2.33634300	-2.90486900	L
O-O_R	0	2.66736200	-3.65542000	-1.12247900	L
O-O_R	0	2.04495800	5.20893600	0.71214600	L
Rh-	0	0.43522500	-0.07521600	-0.80711400	H
Rh-	0	0.74296700	-0.15570100	-3.21036400	H
N-N_3	0	0.01426700	0.09478800	1.26600500	H
S-S_3+6	0	-0.16583000	-1.23808800	2.28219600	H
O-O_2	0	0.90210000	-2.19820600	2.00269800	H
O-O_R	0	-1.59850400	-2.00860300	1.71823800	H
O-O_2	0	-0.44672500	-0.76593400	3.65613500	H
C-C_R	0	-2.79645100	-1.54514500	2.26899600	H
C-C_R	0	-3.26467600	-2.08782200	3.47180300	H
C-C_R	0	-3.55400100	-0.58281000	1.58278900	H
C-C_R	0	-4.48665700	-1.65092200	3.99898300	H
H-H_	0	-2.66828200	-2.84486900	3.98436400	H
C-C_R	0	-4.76469900	-0.14854900	2.13701400	H
H-H_	0	-3.19924300	-0.20018600	0.62161600	H
C-C_R	0	-5.25892900	-0.66124600	3.35710200	H
H-H_	0	-4.82973900	-2.09306200	4.93617100	H
H-H_	0	-5.33744400	0.61029200	1.59960100	H
C-C_3	0	-6.55907200	-0.09934500	3.96907800	H
C-C_3	0	-6.27344800	1.34035900	4.47567100	H
H-H_	0	-5.91483400	1.99169900	3.66336800	H
H-H_	0	-7.18742400	1.79263600	4.89851100	H
H-H_	0	-5.49924700	1.33247100	5.26076800	H
C-C_3	0	-7.05813800	-0.94425800	5.16256200	H
H-H_	0	-8.00992600	-0.53209600	5.53706300	H
H-H_	0	-7.23810700	-1.99289100	4.87203100	H
H-H_	0	-6.34595100	-0.93610400	6.00405400	H
C-C_3	0	-7.69289600	-0.06070800	2.91342300	H
H-H_	0	-8.60250600	0.38428300	3.35161300	H
H-H_	0	-7.42719200	0.53869000	2.02865200	H
H-H_	0	-7.94541500	-1.07678300	2.57014100	H
C-C_R	0	2.23553300	1.14701600	3.19068100	H
C-C_R	0	3.38815300	0.71694800	2.46674900	H
C-C_R	0	4.45362200	0.10833500	3.12554300	H
C-C_R	0	4.39474300	-0.11931200	4.51157500	H

C-C_R	0	3.24766800	0.25309800	5.23723000	H
C-C_R	0	2.18046100	0.87242400	4.59146100	H
H-H_	0	3.41559400	0.85808800	1.38425900	H
H-H_	0	5.34200500	-0.18669700	2.56495000	H
H-H_	0	5.23412100	-0.59787500	5.02334600	H
H-H_	0	3.19022900	0.05137200	6.31012500	H
H-H_	0	1.28876700	1.13926800	5.16041900	H
C-C_R	0	1.21041600	1.86633100	2.50077900	H
C-C_3	0	0.11474800	2.63694500	3.18928400	H
C-C_3	0	0.75081800	3.87961900	3.88687500	H
H-H_	0	1.15025000	4.58628100	3.14279800	H
H-H_	0	-0.02272000	4.40593900	4.47033400	H
H-H_	0	1.56947600	3.59665700	4.56469700	H
C-C_3	0	-1.00348700	3.11179600	2.24027600	H
H-H_	0	-0.58674000	3.56597900	1.32650000	H
H-H_	0	-1.68145500	2.29843200	1.93679000	H
H-H_	0	-1.62894200	3.86640100	2.74370900	H
H-H_	0	-0.33009000	2.00416600	3.97718800	H
H-H_	0	1.41802300	2.13324600	1.45995200	H
H-H_	0	-0.84174900	0.66174400	1.37238500	H
